# Heat-related urban mortality attributable to human-induced climate change in Europe from 2000 to 2025

**DOI:** 10.1038/s44220-026-00674-w

**Published:** 2026-06-30

**Authors:** Clair Barnes, Pierre Masselot, Di Xi, Ben Clarke, Archie Rayner, Xinyi Chen, Ruohan Wang, Emmanuel Raju, Emily Theokritoff, Malcolm Mistry, Antonio Gasparrini, Friederike Otto, Ana Maria Vicedo-Cabrera, Garyfallos Konstantinoudis

**Affiliations:** 1 Centre for Environmental Policy, Imperial College London, London, UK; 2 Environment and Health Modelling Lab, London School of Hygiene and Tropical Medicine, London, UK; 3 School of Public Health, Imperial College London, London, UK; 4 Grantham Institute for Climate Change and the Environment, Imperial College London, London, UK; 5 Department of Public Health, University of Copenhagen, Copenhagen, Denmark; 6 Department of Economics, Ca’ Foscari University of Venice, Venice, Italy; 7 Institute of Social and Preventive Medicine, Universität Bern, Bern, Switzerland; 8 Oeschger Center for Climate Change Research, Universität Bern, Bern, Switzerland

## Abstract

Anthropogenic climate change is increasing the frequency and intensity of extreme heat, with major implications for population health. Although previous studies have attributed heat-related mortality to climate change during individual events, the longer-term contribution of human-induced warming to urban mortality burdens across Europe remains poorly quantified. Here, we combine probabilistic climate attribution with city- and age-specific temperature–mortality relationships to estimate heat-related mortality in 854 European cities during the summers of 2000–2025 under factual and counterfactual climate conditions. We estimate 430,365 heat-related deaths over the study period, with just over half of the modelled burden attributable to human-induced warming. The proportion of estimated heat deaths attributable to human-induced climate change shows a marked rise over time, increasing from 39% in 2000 to 68% in 2025. Although summer 2003 remains the second deadliest summer in absolute terms, summers with similarly large expected mortality burdens have become more frequent in recent years. These findings show that anthropogenic climate change has already greatly increased expected heat-related mortality across European cities, underscoring the need for both rapid emissions reductions and targeted local adaptation.

By 2025, human activities had warmed the planet by approximately 1.3 °C relative to the preindustrial baseline^1^. In 2024, global mean temperature exceeded 1.5 °C above preindustrial levels for the first time, with an estimated 1.36 °C of this attributable to anthropogenic greenhouse gas emissions, primarily from fossil fuel combustion^2^. One of the clearest and most immediate consequences of this warming is the increasing frequency, intensity and duration of heatwaves across most inhabited regions^3,4^. Extreme heat poses a substantial threat to human health, particularly in urban areas, where high population density and the urban heat island effect can amplify exposure and social and infrastructural vulnerabilities and therefore risk^5–14^. As temperatures continue to rise, a central question for climate science and public health is how much of the current burden of heat-related mortality is attributable to human-induced climate change^15,16^. Clear evidence on these trends is needed to understand how risks may develop under future warming.

Previous attribution studies have shown that anthropogenic climate change has substantially increased heat-related mortality. Analyses of major heat events, including the 2003 European heatwave, summer 2022 in Europe, summer 2023 globally and the summer of 2025 in Europe, consistently indicate that human-induced warming has contributed substantially to heat-related deaths^16–22^. However, most of this evidence is derived from individual extreme events or short time periods. As a result, much less is known about how the mortality burden attributable to anthropogenic climate change has evolved over time across a large and diverse set of locations. The only study to date to systematically quantify this contribution over multiple decades and regions analysed 732 locations worldwide from 1991 to 2018 and estimated that 37% of warm-season heat-related deaths were attributable to anthropogenic climate change across all countries^15^. However, given the rapidly increasing rate of observed warming, this figure likely underestimates the current risk.

In this study, we assess the impact of human-induced climate change on heat-related mortality in 854 European cities during the summers of 2000–2025. Using an established probabilistic attribution framework^23^, we quantify temporal trends in heat-related deaths and in the proportion of these deaths attributable to anthropogenic climate change. We present detailed results for 2025, the most recent summer at the time of writing, and a comparison to 2003, the hottest summer in the study period, and examine how the impacts of anthropogenic warming differ across European cities. We focus on Europe because it is the fastest-warming and fastest-ageing continent and because it offers highquality epidemiological evidence across a large and climatically diverse set of cities^24^. A city-scale perspective is especially important because urban populations are highly exposed and often particularly vulnerable to heat, while evidence remains uneven across Europe, especially outside the largest metropolitan areas and in parts of Eastern Europe.

## Results

We implemented a two-step analysis. First, we obtained factual daily temperature time series for 854 cities across Europe from 2000 to 2025 and used probabilistic attribution methods to simulate counterfactual temperature series under a world without anthropogenic climate change. We then estimated daily heat-related mortality under both factual and counterfactual conditions by combining these temperature series with publicly available, location-specific exposure–response functions and with population and mortality data for the same 854 cities^24^. We estimated heat-related mortality attributable to anthropogenic climate change as the difference between factual and counterfactual mortality burdens and as the proportion of factual estimated heat-related deaths attributable to anthropogenic climate change. Because the study focused on heat exposure, analyses were restricted to the summer months of June, July and August. To isolate the contribution of temperature changes due to anthropogenic climate change, the only difference between the factual and counterfactual scenarios is the attributable difference in daily temperatures; exposure–response functions, baseline mortality rates and population values were held constant. Further details are provided in the Methods. We note that the attributable deaths reported here do not represent observed counts but modelled estimates under the framework defined above.

### Attributable trends in temperatures

**Fig. 1:**
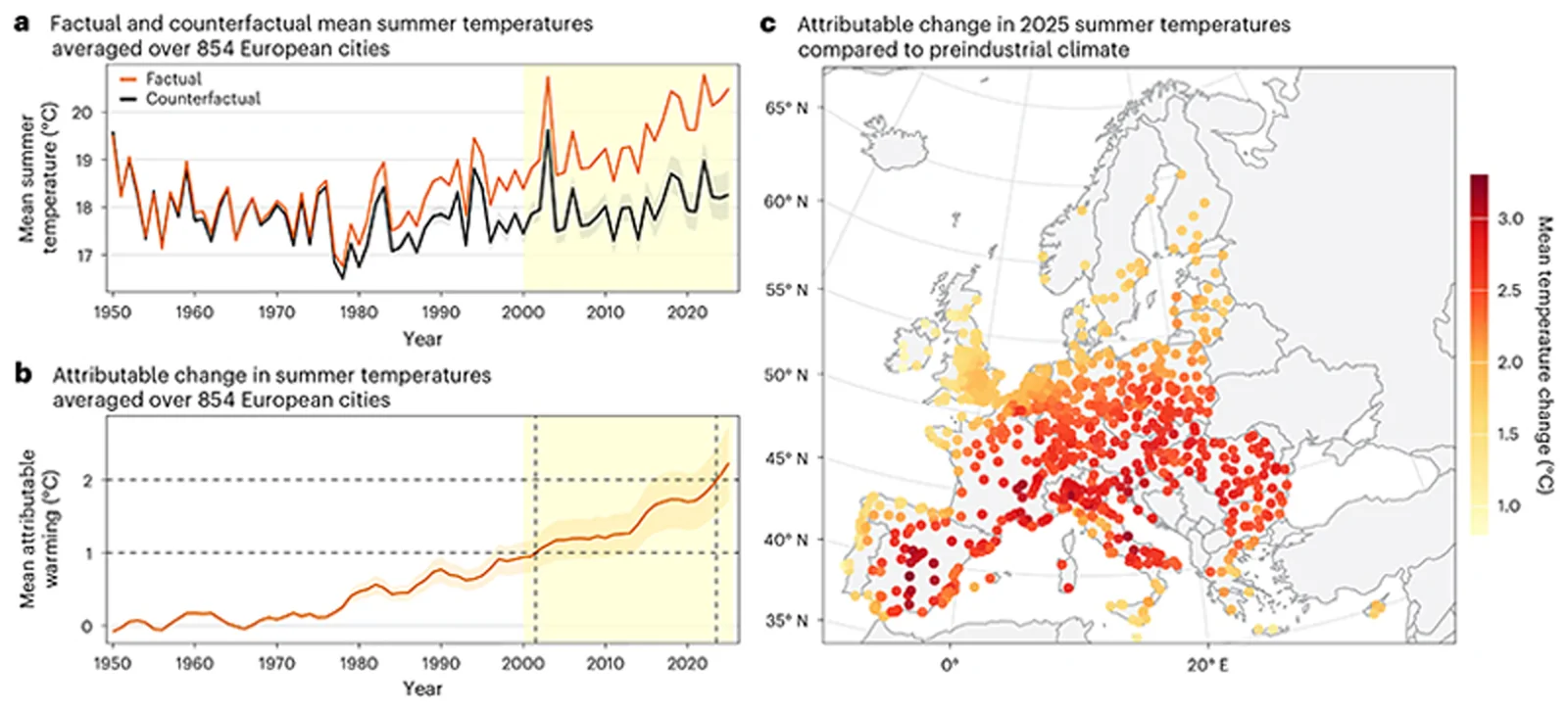
Local warming in 854 European cities attributable to human-induced climate change from 2000 to 2025. **a**, Annual mean summer (June–August) temperatures from 1950 to 2025, averaged over the 854 cities in the study in the factual (red) and counterfactual (black) climates; the grey shaded region represents the 95% CI obtained by block bootstrapping, and the yellow shaded region indicates the study period (2000–2025). **b**, Mean attributable change in summer temperatures over all 854 cities, with the dashed lines showing when 1 °C and 2 °C were exceeded. The orange shaded region represents the 95% CI obtained by block bootstrapping, and the yellow shaded region indicates the study period (2000–2025). **c**, Change in mean summer temperatures in 2025 attributable to human activity in each of the 854 cities. Map data in **c** from Natural Earth (https://www.naturalearthdata.com).

Figure 1a shows the mean summer temperatures averaged over the 854 cities in the study region since 1950, under both the factual and counterfactual scenarios. Mean attributable summer warming across the 854 cities exceeded 1 °C in 2002 and exceeded 2 °C in 2024 (Fig. 1b). Figure 1c shows the change in mean summer temperature in 2025 attributable to human activity (factual versus counterfactual temperatures) across the 854 cities. The highest levels of human-induced warming are observed in northern Italy, central Spain, southern France and parts of Eastern Europe, with increases of more than 2.5 °C attributable to human activities.

### Heat-related mortality burden

**Fig. 2:**
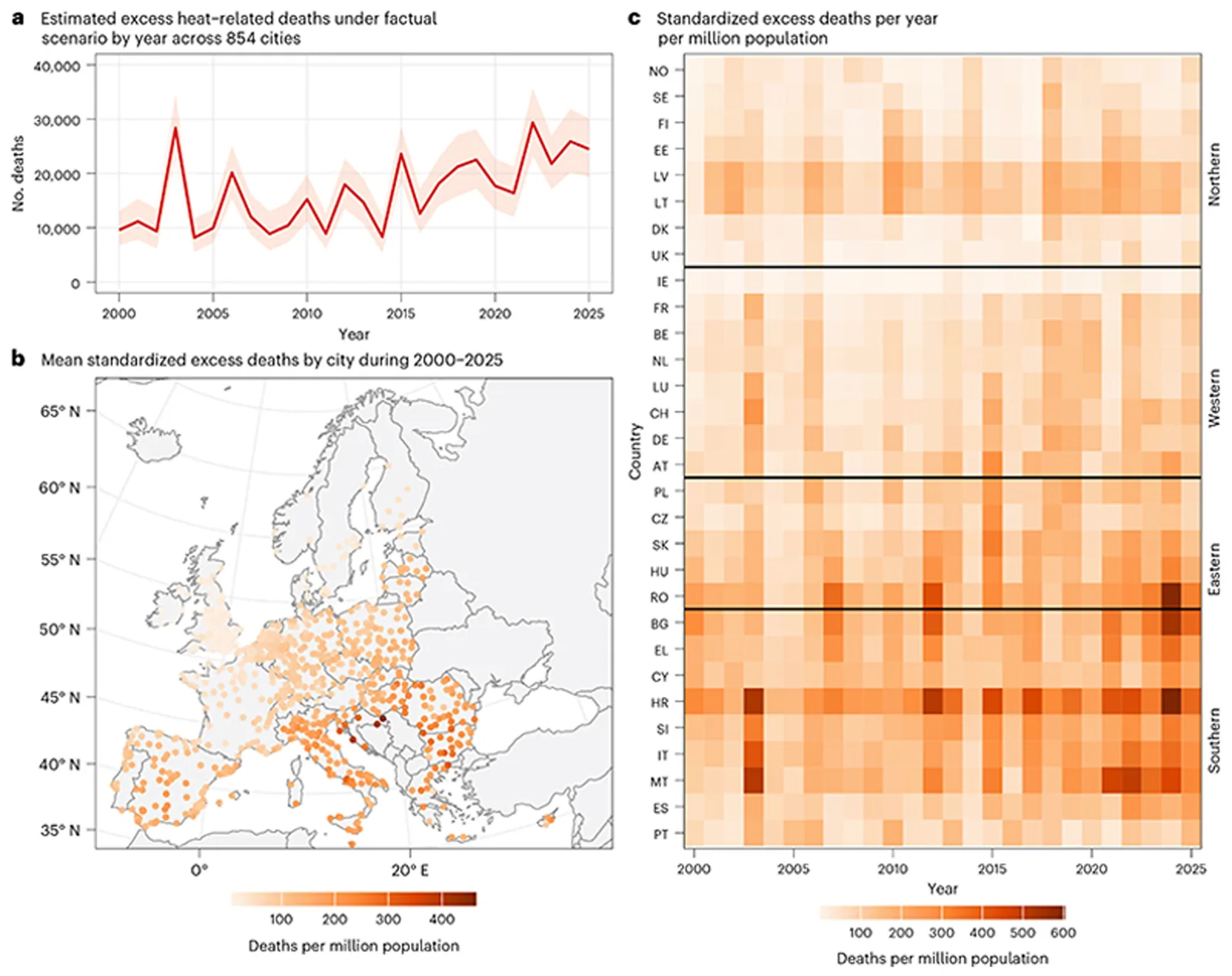
Modelled heat-related excess deaths in 854 European cities from 2000 to 2025. **a**, Estimated total heat-related excess deaths across the 854 cities under the factual scenario from 2000 to 2025. The shaded region represents the 95% CI. **b**, Mean standardized excess deaths per 1,000,000 population during 2000–2025 in the 854 cities. **c**, Mean standardized excess deaths per 1,000,000 population by year and country from 2000 to 2025. Country abbreviations: AT, Austria; BE, Belgium; BG, Bulgaria; CH, Switzerland; CY, Cyprus; CZ, Czech Republic; DE, Germany; DK, Denmark; EE, Estonia; EL, Greece; ES, Spain; FI, Finland; FR, France; HR, Croatia; HU, Hungary; IE, Ireland; IT, Italy; LT, Lithuania; LU, Luxembourg; LV, Latvia; MT, Malta; NL, Netherlands; NO, Norway; PL, Poland; PT, Portugal; RO, Romania; SE, Sweden; SI, Slovenia; SK, Slovakia; UK, United Kingdom. Map data in **b** from Natural Earth (https://www.naturalearthdata.com).

Across the 854 European cities included in this study, with a combined population of almost 160 million residents, we estimated a factual 430,365 (95% empirical confidence interval (eCI) 376,535–481,458) heat-related deaths during the summer months over 2000–2025 (Table 1). The highest burden was observed among individuals aged ≥85 years, with 165,992 (95% eCI 147,573–185,609) estimated heat-related deaths. The annual estimated heat-related mortality burden increased over the study period, from 9,720 (95% eCI 8,162–11,206) deaths in 2000 to 24,572 (95% eCI 22,064–26,867) in 2025 across the 854 cities (Fig. 2a and Extended Data Table 1). The summers of 2022 and 2003 had the highest annual estimated heat-related death counts, at 29,480 (95% eCI 26,643–32,006) and 28,462 (95% eCI 25,709–31,199), respectively.

Among the 30 countries analysed, Croatia, Malta and Romania exhibited the highest mean standardized excess mortality per 1,000,000 population over 2000–2025 (Table 2): 327 estimated heat deaths per 1,000,000 population (95% eCI 245–402) in Croatia, 226 (95% eCI 163–288) in Malta and 223 (95% eCI 150–305) in Romania. Among the 30 capital cities, Zagreb, Bucharest and Rome showed the highest mean standardized excess mortality per 1,000,000 population, with average annual estimated heat-related death counts of 314 (95% eCI 222–400), 268 (95% eCI 200–360) and 241 (95% eCI 197–283), respectively (Extended Data Table 2). Over the study period, the highest standardized excess per 1,000,000 population was consistently estimated in southern and southeastern Europe (Fig. 2b), with the summers of 2003, 2022 and 2024 standing out as particularly dangerous in southern Europe (Fig. 2c).

### Heat-related mortality attributable to anthropogenic climate change

**Fig. 3:**
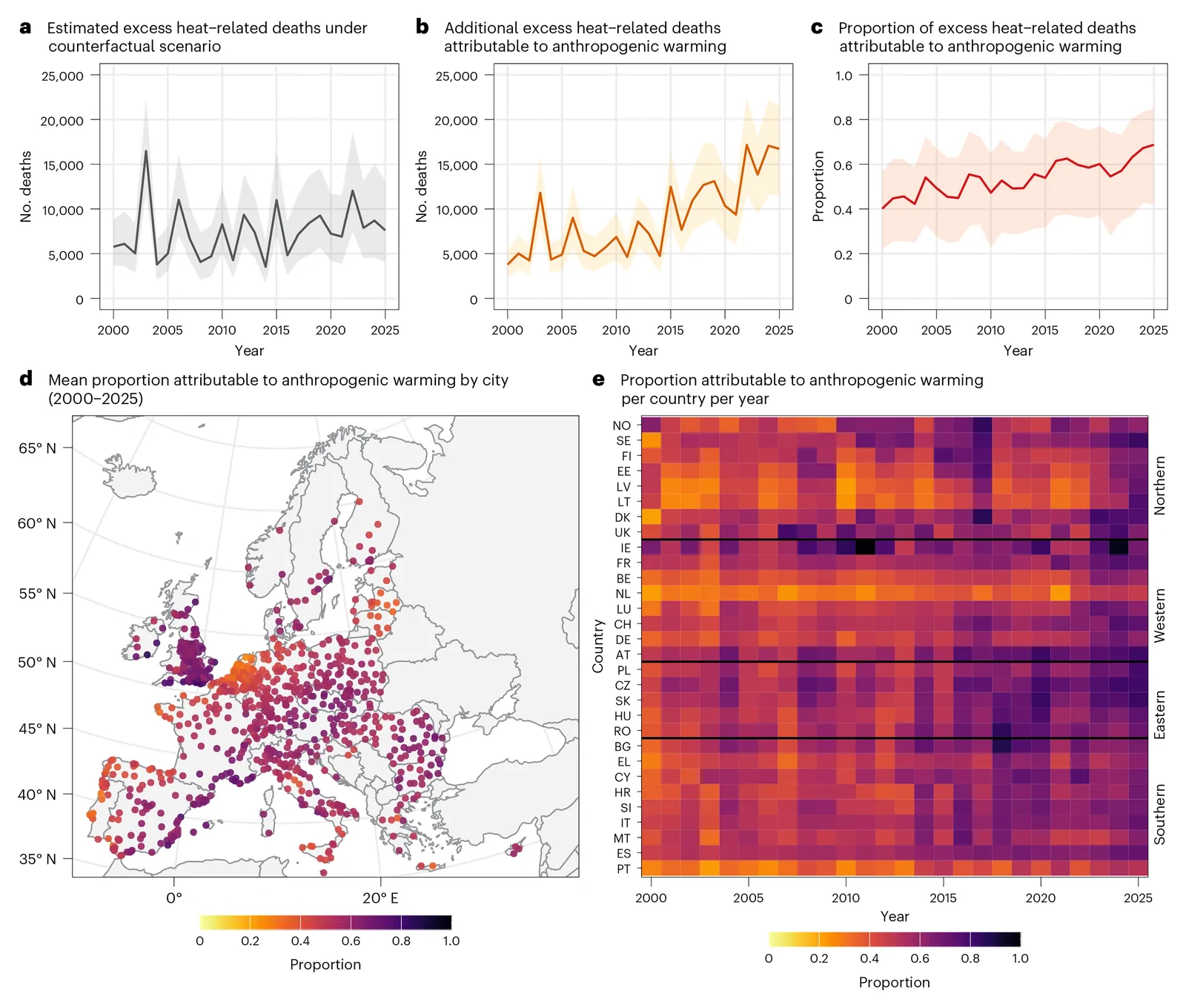
Proportion of modelled heat-related excess deaths attributed to human-caused warming in 854 European cities from 2000 to 2025. **a**, Estimated total heat-related excess deaths across the 854 cities under the counterfactual scenario from 2000 to 2025. **b**, Difference between total heat-related excess deaths in factual and counterfactual scenarios. **c**, Proportion of heat-related excess deaths attributable to climate change. In all panels, the line indicates the median estimate, and the shaded region represents the 95% CI. **d**, Mean proportion attributed to human-induced climate change in 854 cities from 2000 to 2025. **e**, Mean proportion attributed to human-induced climate change per country and year from 2000 to 2025. Map data in **d** from Natural Earth (https://www.naturalearthdata.com).

Compared with the counterfactual temperature scenario, we estimated 233,626 additional heat-related deaths (95% eCI 184,565–274,584) attributable to anthropogenic climate change over the study period, suggesting that more than half of the total heat-related mortality burden estimated between 2000 and 2025 can be linked to human-driven climate change (Table 1). The proportion of heat-related deaths attributable to anthropogenic climate change was highest among individuals aged ≥85 years (0.58; 95% eCI 0.47–0.66; Table 1). The estimated number of heat-related deaths under the counterfactual scenario remained relatively constant across the study period (Fig. 3a), whereas the additional excess and the proportion of excess heat deaths attributable to anthropogenic warming increased substantially (Fig. 3b,c). The proportion of heat-related deaths attributable to anthropogenic climate change rose from 0.39 (95% eCI 0.30–0.47) in 2000 to 0.68 (95% eCI 0.56–0.77) in 2025 (Extended Data Table 1).

The highest attributable proportions were estimated for Ireland (0.67; 95% eCI 0.19–0.90), Austria (0.64; 95% eCI 0.45–0.78) and Czechia (0.63; 95% eCI 0.46–0.76; Table 2). Coastal cities, particularly those along the Atlantic and North Sea coastlines, tended to show lower proportions of heat-related mortality attributable to anthropogenic warming (Fig. 3d). The proportion of heat-related deaths attributable to anthropogenic warming varied considerably across both time and geography among the 30 countries studied (Fig. 3e) but increased throughout the study period in all countries.

### Comparison of summer 2003 and summer 2025

**Extended Data Fig. 1:**
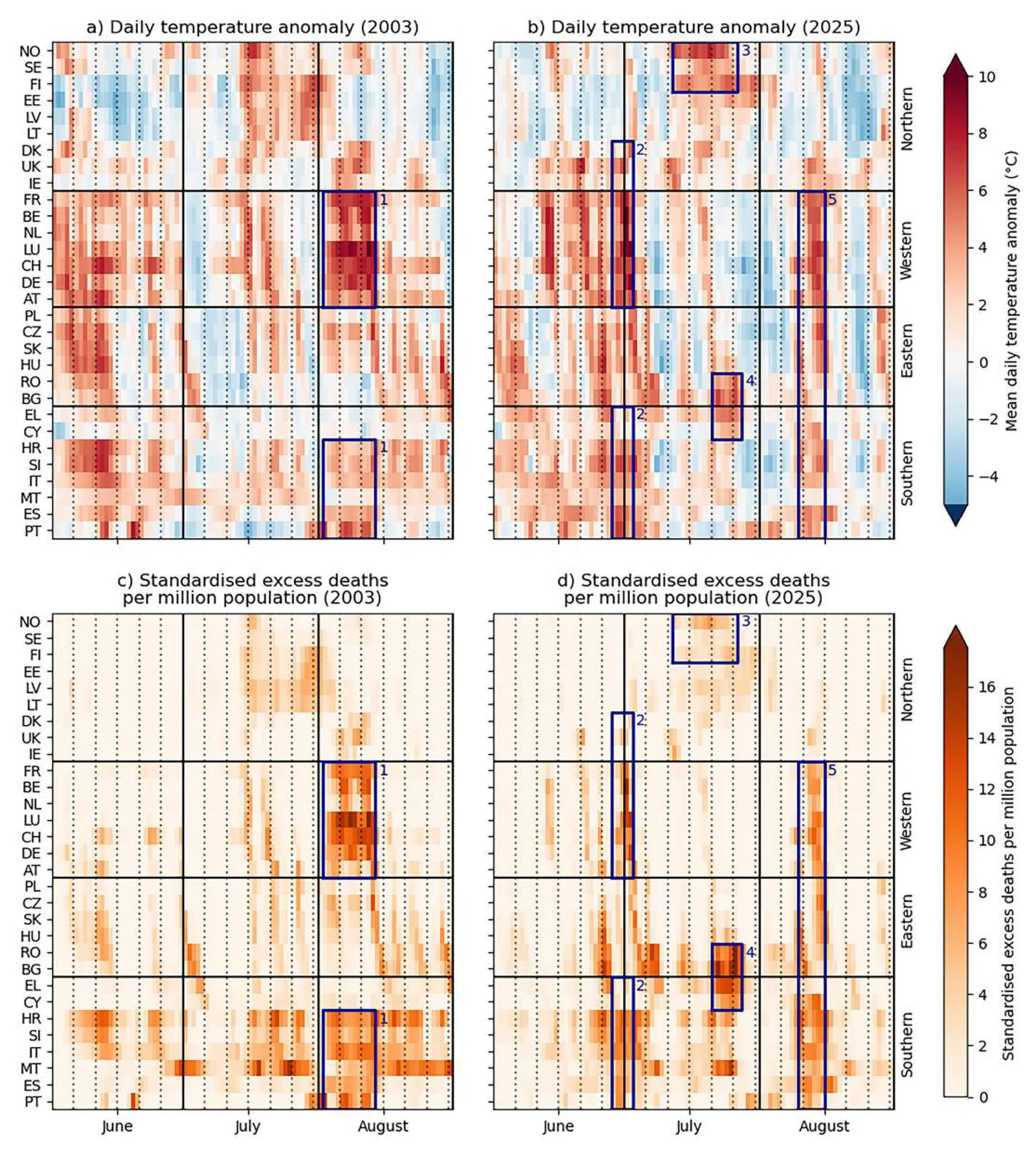
Heatmaps of daily mean temperature anomaly and standardised excess death rate per country in 2003 and 2025. Daily mean temperature anomaly (°C) relative to the 1990-2020 daily mean over a two-week moving window centred on each date in (**a**) 2003 and (**b**) 2025; and standardized excess deaths per million population for the summers of 2003 and 2025 across 30 European countries in (**c**) 2003 and (**d**) 2025. Numbered boxes indicate periods of particularly dangerous heat that have previously been the subject of attribution studies. (1) The 2003 heatwave; (2) early spring heatwave triggering heat health warnings across Europe; (3) 2025 Fennoscandia heatwave; (4) record-breaking heatwaves linked to wildfires in the Balkans; (5) record-breaking heatwaves linked to wildfires in the Iberian peninsula.

**Fig. 4:**
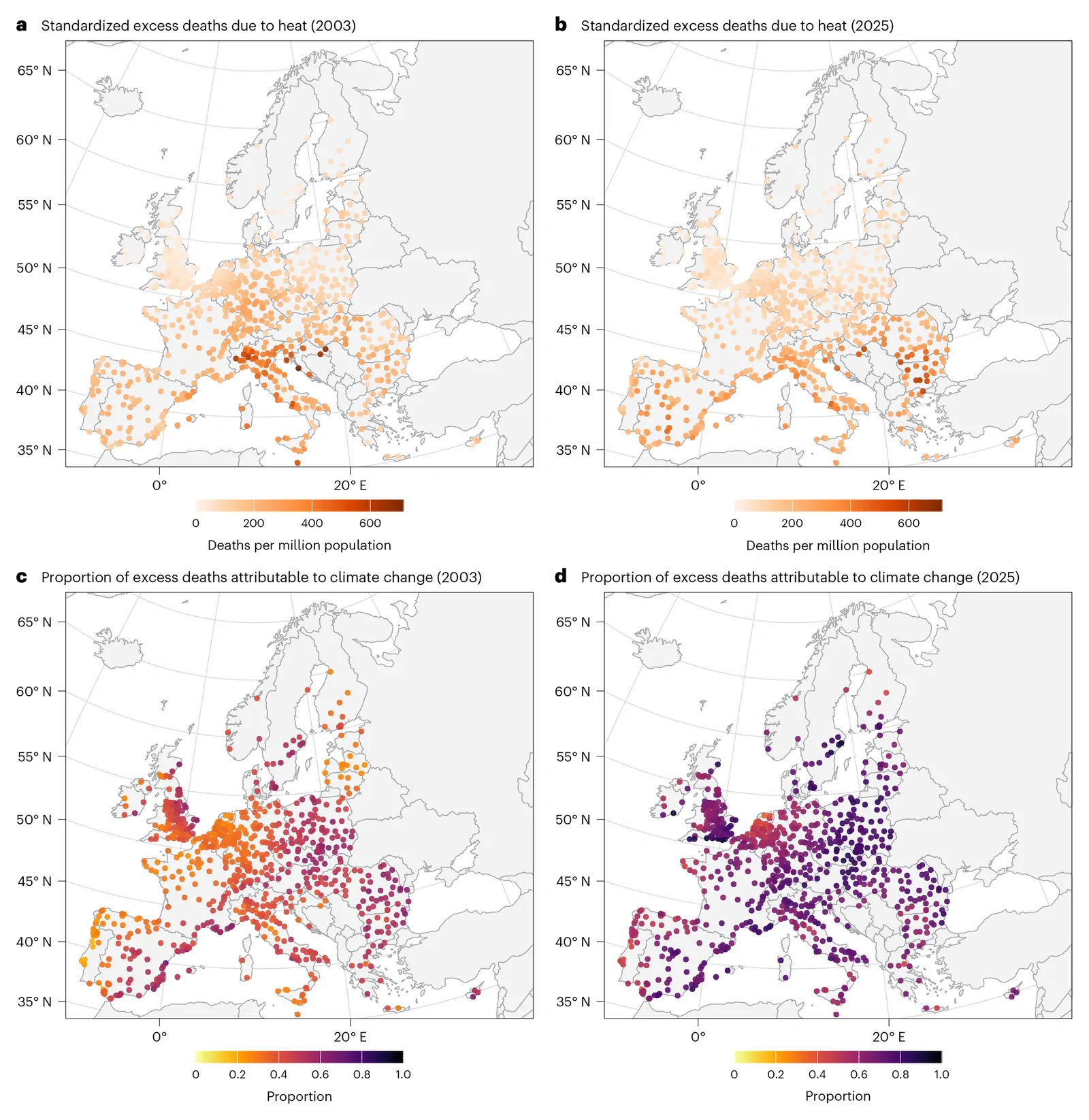
Comparison of modelled heat-related excess deaths and proportion attributable to human-caused warming in 2003 and 2025. **a**–**d**, Maps showing the median of annual age-standardized excess deaths due to heat in 2003 (**a**) and 2025 (**b**); and proportion of excess deaths attributable to climate change in 2003 (**c**) and 2025 (**d**) in each of the 854 cities. Map data from Natural Earth (https://www.naturalearthdata.com).

Standardized heat-related excess mortality showed similar geographical patterns in the summers of 2003 and 2025 (Fig. 4a,b); however, the proportion attributable to anthropogenic climate change was markedly higher in 2025 (Fig. 4c,d). In both summers, the strongest impacts were concentrated in eastern and southern Europe, with Valletta, Rome and Zagreb showing the highest mean standardized excess mortality among the 30 capitals in 2003 and Bucharest, Rome and Zagreb in 2025 (Extended Data Tables 3 and 4). In 2003, the heat signal was concentrated in early August and was closely mirrored by sharp spikes in daily excess mortality, particularly in western and southern Europe (Extended Data Fig. 1, box 1). By contrast, summer 2025 was characterized by temperature spikes in June and early July, followed by a shorter heatwave in mid-August (Extended Data Fig. 1, boxes 2–5). Although summer 2003 resulted in a higher estimated number of deaths than summer 2025, only 42% of those deaths were attributable to anthropogenic climate change (95% eCI 33–50%). By contrast, 68% of the estimated heat-related deaths in 2025 were attributable to human activity (95% eCI 56–77%), suggesting that the role of anthropogenic warming in driving heat mortality has grown substantially and uniformly across the 854 cities (Fig. 4c,d and Extended Data Table 1).

### Sensitivity analysis

In a sensitivity analysis, we used variable populations and mortality rates across the study period to test the effect of the assumption of fixed population and mortality rates used in the analysis above. Although the number of heat deaths in 2025 became comparable with 2003, reflecting increases in population across the years, the proportion of deaths attributed to anthropogenic climate change was consistent across the approaches (Supplementary Information, section S1, and Extended Data Tables 5 and 6).

In a second sensitivity analysis, we restricted the variable population and mortality rate approach to the subset of countries with mortality data available during the summer months, enabling the derivation of seasonally relevant baseline mortality rates for the June–August study period (Extended Data Table 7). Overall, the results were consistent with Table 2.

## Discussion

In this heat mortality attribution study of 854 European cities, we estimated the number and proportion of expected heat-related deaths that would have been avoided in a world without anthropogenic climate change. Overall, we estimated that more than half of the modelled heat-related deaths over the entire period from 2000 to 2025 could be attributed to human-induced climate change, with the largest burden falling on the oldest age groups. The proportion increased from 0.39 in 2000 to 0.68 in 2025, indicating that the ongoing climate crisis has already had a substantial impact on heat-related mortality in European urban populations. The impacts were highly heterogeneous across countries, with eastern and southern Europe (Croatia, Malta, Romania and Italy) bearing the highest estimated heat-related mortality burden. The largest number of modelled heat-related deaths occurred in 2022, with the second largest estimated number during the extreme summer of 2003; however, that number was not substantially higher than the estimates for 2024 and 2025. As the climate warms, dangerously hot summer temperatures are becoming increasingly frequent and, with them, a steadily growing proportion of heat-related deaths are attributable to anthropogenic climate change.

The analysis builds on the first rapid mortality attribution study published in summer 2025^21^ and extends the results to 854 cities^22^ over a 26-year period. We used an established methodology for disentangling the impact of human-induced climate change^23^, coupled with state-of-the-art approaches for estimating exposure–response functions^24^. By integrating climate model simulations with large-scale mortality datasets, our analysis provides an unprecedented level of spatial, temporal and age-specific detail for quantifying the health impacts of increasing heat across Europe, with direct relevance for local public health planning and urban climate adaptation policy. Finally, by examining the robustness of the approach and discussing and comparing our estimates with previous studies, we provide further support for its use in future rapid attribution analyses of heat-related mortality.

This study assumes a constant baseline daily mortality rate throughout the study period, derived from historical mortality data^24^. In a sensitivity analysis, we relaxed this assumption and found that the proportion of deaths attributed to human-induced climate change was broadly consistent across methods. However, these results also suggest that our estimates may underestimate the absolute heat-related mortality burden, particularly in the most recent years. A related assumption is that baseline mortality remains constant within summers. Because mortality tends to rise during heatwaves, the underlying death rate on very hot days likely exceeds the constant rate assumed here. Similarly, because temperatures were averaged across each study area from gridded data with a spatial resolution of approximately 11 km, the exposure metric used in the analysis is an area-level daily mean temperature and does not capture within-area heterogeneity in heat exposure. Therefore, the analysis cannot account for finer-scale variation related to local urban heat island effects, green space, housing conditions, indoor environments, air conditioning, socioeconomic gradients or the spatial distribution of older adults. In addition, daily mean temperature does not capture all dimensions of heat exposure, such as daytime extremes, nighttime heat, humidity, apparent temperature or heatwave duration. All of these factors may further contribute to an underestimation of the absolute heat burden.

The exposure–response relationships used in the attribution analysis were derived from health data spanning from 2000 to 2019 and are assumed to remain constant throughout the study period. As a result, we do not explicitly account for potential changes in population vulnerability over time. On one hand, adaptation, through improvements in public health preparedness, housing, urban planning and health care systems, may have attenuated local heat–mortality associations over time^25–27^. On the other hand, our analyses do not capture potential maladaptation, demographic ageing or increases in the prevalence of chronic conditions, all of which could heighten local vulnerability^28^. Not accounting for adaptation may affect not only the absolute burden estimates but also the estimated proportion of heat-related deaths attributable to anthropogenic climate change, although the direction and magnitude of this bias are uncertain as they depend on how adaptation has altered the shape of the exposureresponse curve. Importantly, assuming constant baseline mortality and exposure–response relationships allows us to isolate the contribution of temperature exposure and evaluate heat mortality across varying summer conditions. These results are characterized at the city level and may not be representative of conditions across entire countries, limiting the extent to which our findings can be extrapolated to national scales. Additionally, the attributable warming is likely a conservative estimate, given the tendency of climate models to underestimate long-term warming trends (Supplementary Information, section S2). Finally, we note that the eCIs do not capture all potential sources of uncertainty. In particular, uncertainty related to population data, baseline mortality rates, study area boundary definitions, exposure assignment and climate model structural uncertainty was not incorporated. Therefore, the reported intervals may underestimate the full uncertainty surrounding the impact estimates.

We note that our results differ from those of the first mortality attribution study focusing on summer 2003, which estimated that approximately 20% of heat-related deaths in London and 70% in central Paris were attributable to anthropogenic climate change^17^, whereas we estimate 36% (95% eCI 23–50%) in London and 30% (95% eCI 22–41%) in Paris (Extended Data Table 3). However, Mitchell et al. only account for heat-related mortality on days with apparent temperatures exceeding a fixed threshold for each city and multiply this by the fraction of attributable risk, which represents the change in likelihood of exceeding the modelled number of heat deaths in both the factual and counterfactual scenarios^17^. This approach therefore answers a fundamentally different question than our analysis, which includes the possibility of heat-related mortality even on moderately warm days, meaning that the estimates cannot be compared directly^29^. Other than this, our results are broadly consistent with previous studies. In line with previous studies, the highest burden was observed in older populations, reflecting a combination of higher baseline mortality, greater prevalence of chronic conditions and reduced thermoregulatory capacity^9,24^. In agreement with analyses focusing on summer 2022 in Europe, we estimate that 59% of heat-related deaths in summer 2022 (95% eCI 46–68%) were attributable to humaninduced climate change, with the highest proportion in older populations^19,20^ (Table 1 and Extended Data Table 1). A global study covering the period from 1991 to 2018 reported that about 37% of heat-related deaths were attributable to climate change, which is comparable to the proportions we observe in the early years of our study period. Another global analysis of the 2023 heatwave estimated that 54% of heatwave-related deaths were attributable to human-induced climate change^16^, slightly smaller than our estimate of 64% but well within the CI (95% eCI 51–73%). We estimated an annual average of 16,553 heat-related deaths during the summer months over the period from 2000 to 2025 (95% eCI 14,482–18,518). This figure is notably higher than the estimate reported by a previous study that used the same underlying exposure–response functions, which found 13,589 deaths per year (13,589; 95% eCI 11,530–15,475)^24^. The discrepancy is likely driven by the fact that our analysis extends beyond 2019, a period characterized by exceptionally high heat-related mortality not captured in the earlier work. Our temporal estimates of the proportion of deaths attributable to human-induced climate change are broadly consistent with those reported for the summers from 2015 to 2021 (ref. 19). In line with previous studies, we observed higher rates of heat-related mortality in southern Europe^9,15,24^, with Croatia, Malta and Romania being the most affected countries. This pattern likely reflects the persistently higher ambient temperatures in these countries, rather than a greater relative risk of heat-related mortality^24^.

Comparisons with official mortality estimates must be made cautiously because the methods used differ from those presented here in several important respects. Our analysis focuses on adults aged over 20 years and uses city-level estimates over the full summer period to quantify mortality attributable to ambient heat using a parametric temperature–mortality relationship. By contrast, official estimates may cover different age groups, use regional rather than city boundaries, focus on heatwave periods rather than ambient heat across the summer and estimate excess mortality relative to a baseline period. For example, for summer 2025, the UK Health Security Agency estimated 317 heat-related deaths in London (95% eCI 139–495) across all age groups, compared to 416 (95% eCI 274–554; Extended Data Table 4) in our analysis^30^, and Euromomo reported 451 heat-attributable deaths in Madrid across all age groups, compared to 544 (95% eCI 399–699; Extended Data Table 4) in our analysis (https://momo.isciii.es/panel_momo/). Both approaches have important strengths and limitations and are best viewed as complementary rather than competing. For instance, estimating excess mortality relative to a reference period is particularly well suited to describing extreme events, uses directly observed mortality and has the advantage of not requiring assumptions about the functional form of the temperature–mortality relationship. However, the resulting estimates can depend strongly on the choice of baseline period, model specification and demographic adjustment^31^. By contrast, our approach does not account for population adaptation to heat and uses estimated baseline mortality but enables estimation of counterfactual heat-attributable deaths, supports real-time forecasting and provides a framework for rapid health attribution.

Our analysis shows that anthropogenic climate change has already become a major driver of heat-related mortality in European cities, accounting for more than half of the estimated heat-related deaths over the 2000–2025 study period. This burden is unevenly distributed: impacts are greatest in southern Europe and during extreme summers and appear to be driven primarily by changes in the frequency and intensity of heat exposure rather than by differences in exposure–response relationships alone. With mortality rates and temporal variation of exposure held constant, and allowing variation by age and space, the contribution of anthropogenic warming to heat mortality increased steadily over time, suggesting that summers with mortality burdens comparable to the devastating 2003 heatwave are becoming an increasingly regular occurrence.

These findings carry important policy implications. The pronounced spatial variation in risk underscores the need for locally tailored public health and climate adaptation planning, particularly for older and vulnerable populations in southern Europe. At the same time, adaptation measures alone are unlikely to fully offset the health risks associated with rising temperatures: our results suggest that a substantial share of expected heat-related mortality is linked to anthropogenic warming, and this increase could in principle be reduced under stronger mitigation. Without deep and sustained emissions reductions, the health burden associated with continued warming is expected to increase, placing growing pressure on public health systems.

## Methods

### Ethics

This study uses secondary, aggregate anonymized data, so no ethical permission is required.

### Climate attribution

The attribution analysis was performed using an established probabilistic attribution methodology^23,32–34^. First, time series of daily mean temperatures in each of the 854 cities during the northern hemisphere summer months (June–August) were retrieved from gridded data products. For each time series, a statistical model was used to estimate the relationship between the monthly mean temperature and the global mean surface temperature (GMST). Results from both observation-based datasets and climate models were combined to estimate the trend in local temperatures as a function of global temperatures and so to estimate the expected change in temperatures associated with the 1.3 °C of human-caused warming that occurred between the pre-industrial period (1850–1900) and 2025.

### Observational temperature data

Two observational data products were used to estimate historic warming trends in this study. ERA5-Land is a gridded reanalysis dataset that combines historical observations into global estimates using advanced modelling and data assimilation systems, spanning the period from 1950 to 2025^35^ at a resolution of 0.1 ° × 0.1 °, while E-Obs (v31.0e + pre1950) is a land-only product derived from the interpolation of station-derived meteorological observations from 1920 to 2025^36^, at a resolution of 0.1 ° × 0.1 °. For the dataset, we retrieved the daily mean temperature data in each Urban Audit city^37^ by taking the mean over all grid cells intersecting with the polygon defining the region. The ‘factual’ temperatures referred to in the study were taken from ERA5-Land, while both ERA5-Land and E-Obs were used to estimate the historic warming trend.

### GMST data

The annual GMST time series was obtained from the National Aeronautics and Space Administration (NASA) Goddard Institute for Space Science surface temperature analysis^38,39^. To reduce variability in the GMST data due to the El Nino-Southern Oscillation, we used a 4-year rolling average of the GMST.

### Climate model data

The attribution step used a multimodel ensemble of coupled global circulation models from the CMIP6 experiments^40^. All years from 1850 to 2030 were used to estimate the historic warming trend, with each model’s own local temperatures regressed against its own GMST time series. For all simulations, the period from 1850 to 2014 was based on historical simulations, while the SSP5-8.5 scenario was used to project from 2015 to 2030. The results were not expected to be sensitive to the choice of scenario because all trends were estimated in terms of global mean temperature, rather than time, which controls for the different rates of warming in different models and scenarios; furthermore, divergence between the scenarios before 2030 was minimal (see, for example, section 4.3 of the Intergovernmental Panel on Climate Change’s Sixth Annual Assessment Report^41^). In an exploratory analysis comparing the spatial climatology of summer temperatures from 1990 to 2020 with the observed climatology, all models were found to perform well. To avoid using different subsets of climate models in each region, no further selection was performed based on the local seasonal cycles or parameter fits. However, four model variants were removed from an original ensemble of 40 because they were lower-resolution variants of other models within the ensemble, leaving 36 models each providing one realization to the multimodel ensemble. A list of the models included in the study can be found in Extended Data Table 8.

### Statistical modelling of local temperature trends in response to global warming

For each time series (both observations-based and derived from climate models), we estimated the parameters of a nonstationary normal distribution in which the mean monthly temperature depends linearly on GMST, while the variance remains stationary, so that in year *y* and calendar month *m* the local temperature *T* at location *l* is modelled as:

$$\begin{array}{l} T_{yml}=\alpha_{ml}+\beta_{ml}W_{y}+\epsilon_{ml}\\ \epsilon_{ml}\sim N\left(0,\sigma_{ml}^{2}\right) \end{array}$$

where *W*_*y*_ = *G*_*y*_ − (*G*_2025_ − 1.3) is the attributable warming in year *y*, calculated as the difference between the smoothed GMST *G*_*y*_ in year *y* and in a preindustrial period assumed to be 1.3 °C cooler than in 2025^1^. The intercept, slope and scale parameters, {*α*.}, {*β*.} and {*σ*.}, were obtained simultaneously for all months and locations using ordinary least squares estimation. The key parameters of interest in this model are the {*β*_*ml*_}, which denote the change in local monthly mean temperatures per degree of global warming; 95% CIs are obtained by block bootstrapping of all locations and calendar months, using a sample size of 1,000 (that is, by resampling whole years of data for each boot-strap). This procedure was repeated independently for each dataset.

### Synthesis of local warming trends

For each city and month, the estimated trend parameters from the observational products and climate models were combined into a single estimate of the change in local temperatures per degree of global warming using an established synthesis algorithm^34^. We first pooled the observational estimates by taking their mean and inflating the variance with an additional representation-error term (added in quadrature) to capture discrepancies in long-term trends between the observational products. We obtained a separate precision-weighted mean of the model-derived estimates, with a term added in quadrature to account for intermodel spread as well as internal variability. Finally, we took the unweighted mean of the estimates from the observations and the climate models to yield an overall best estimate of the local warming trend with a corresponding 95% CI. We note that this is a departure from the standard protocol defined in that paper, which would usually use inverse-variance weighting of the estimates from the observations and models; however, climate models are known to produce overconfident underestimates of warming in the region (Supplementary Information, section S2), so this approach avoids giving undue weight to the climate models. However, this still results in a lower estimate of attributable warming than the historic warming in observations and should therefore be interpreted as a conservative estimate.

### Calculating counterfactuals

Factual daily mean temperatures for the summers from 2000 to 2025 were taken from ERA5-Land reanalysis^35^. To calculate counterfactual temperatures, we assumed that essentially all forced warming is anthropogenic, with around 1.3 °C of human-caused warming having occurred by 2025^1^, and calculated the anthropogenic global warming *W*_*y*_ in year *y* from the smoothed GMST time series, as defined above. The human-caused local change in temperatures at location *l* in calendar month *m* in year *y* was then calculated by multiplying *W*_*y*_ by the synthesized change in local temperatures per degree of warming (${\tilde{\beta }}_{ml}$, say). Finally, the human-caused local warming was subtracted from the factual temperatures to obtain the counterfactual temperatures that would have been expected in a world without climate change.

### Propagating uncertainty into the mortality analysis

To obtain a proper quantification of the uncertainty in the mortality estimates arising from uncertainty in the attributable warming, it is necessary to obtain a spatially coherent sample of local warming rates. To achieve this, we use the ERA5-Land (‘factual’) block boot-strap to estimate the 2,562 × 2,562 correlation matrix, *C*, between the rates of warming in all 854 cities and all calendar months. We then sampled 1,000 realizations from a multivariate normal distribution with standard normal marginals and correlation matrix *C*. Finally, the marginal distributions were transformed to have the same mean and variance as the synthesized trends for each city and calendar month. This resulted in a spatially coherent sample of the local synthesized warming, with the same correlation structure as the observed rate of warming. This adjustment was then subtracted from the daily factual temperatures to obtain an ensemble of preindustrial counterfactual time series, which were used as inputs to the epidemiological model.

### Mortality attribution

To quantify heat-related mortality attributable to human-induced climate change, we used a framework that integrates exposure–response functions with temperature time series under factual and counter-factual climate conditions. The approach consisted of three main components. First, we extracted age-specific temperature–mortality exposure–response functions for 854 European cities from Masselot et al.^24^. Second, we applied these city- and age-specific functions to the temperature series under both factual and counterfactual scenarios to estimate the number of heat-related deaths in each case. Third, we attributed mortality to climate change by comparing the excess deaths in the factual and counterfactual worlds.

### Exposure–response functions

**Extended Data Fig. 2:**
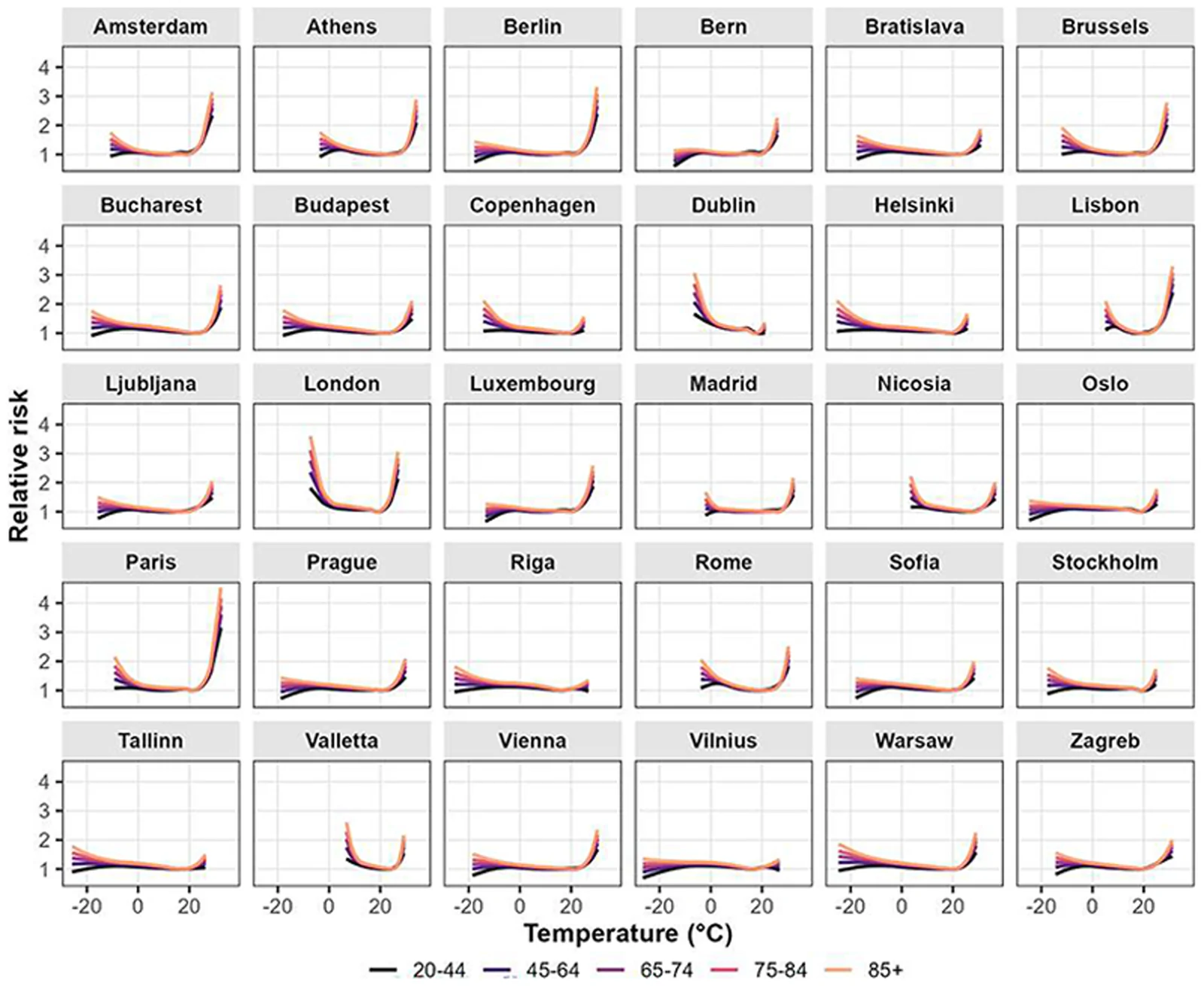
Exposure-response functions for 30 European capital cities. Median ambient temperature-related relative mortality risk by age group (20-44, 45-64, 65-74, 74-85 and 85 + years) in the 30 capitals. Relative risks are expressed with respect to the minimum mortality temperature, defined as the temperature between the 25th and 99th percentiles of the local temperature distribution at which the relative risk is minimised.

Temperature–mortality exposure–response functions were derived from the framework developed previously^24^. Briefly, temperature-related mortality risks were first estimated in 232 European cities with observed daily mortality data for each of the five age groups, using quasi-Poisson regression models combined with distributed lag nonlinear models to account for nonlinear and delayed temperature effects, with adjustment for day of week and long-term and seasonal trends^42^. The parameterization of the exposure–response association used a quadratic B-spline with three internal knots placed at the 10th, 75th and 90th percentiles of the location-specific temperature distribution, while the lag-response function was modelled using a natural cubic B-spline with an intercept and three internal knots equally spaced on the log scale of lag days^43^. These models were fitted to age-specific mortality series, yielding cumulative exposure–response coefficients for each city and age group. In a second stage, these coefficients were pooled in a repeated-measure multivariate meta-regression incorporating age, regional indicators and city-level vulnerability characteristics, with residual spatial heterogeneity modelled through kriging^44^. The resulting model was then used to predict exposure–response functions and associated variance–covariance matrices for each age group in all 854 cities included in our analysis. The full modelling procedure is detailed in refs. 24,45. Extended Data Figure 2 shows the relative risk curves in the 30 European capitals covered by the study.

The exposure–response functions used in this study were derived from daily mortality data from the Multi-country Multi-city Collaborative Research Network (https://mccstudy.lshtm.ac.uk/), fitted against local daily mean temperatures from the ERA5-Land reanalysis dataset for 1990–2019 (ref. 35).

### Health impact assessment

Using the retrieved exposure–response function, we calculated attributable fractions for both factual and counterfactual temperatures^19,20,46^. Daily deaths during the heatwave were estimated assuming a constant rate based on historical Eurostat data^24^. Applying this rate to the attributable mortality fraction yielded excess deaths in each scenario. As the interest was in estimating the impact of heat, attributable fractions were calculated only for temperatures above the minimum mortality temperature (defined as the temperature between the 25th and 99th percentiles of the local temperature distribution at which the relative risk is minimized) and for the summer months of June–August. To quantify the impact of human-induced climate change, we compared excess deaths in the factual and counterfactual scenarios and calculated the number and proportion of heat-related deaths attributable to human activity^21,22^. To propagate uncertainty, we drew 1,000 samples from the multivariate distribution of the basis-function coefficients for each location and age group^24^, allowing the minimum mortality temperature to vary between samples. We combined these with the factual temperature time series and sampled counterfactual time series to obtain a sampling distribution for the attributable fractions and excess deaths in the factual and counterfactual worlds. We summarized the estimates using the median and the 2.5th and 97.5th quantiles, which we report as 95% eCI.

### Reporting summary

Further information on research design is available in the Nature Portfolio Reporting Summary linked to this article.

## Extended Data

**Extended Data Table 1:** Heat-related mortality and number and proportion of deaths attributable to anthropogenic warming per year from 2000-2025, aggregated over all 854 cities

Year	Factual	Counterfactual	Difference	Proportion
2000	9,720 (8,162, 11,206)	5,903 (4,498, 7,296)	3,798 (2,868, 4,637)	0.39 (0.30, 0.47)
2001	11,328 (9,445, 13,023)	6,214 (4,659, 7,838)	5,034 (3,924, 6,123)	0.45 (0.36, 0.53)
2002	9,505 (7,844, 11,167)	5,156 (3,858, 6,575)	4,277 (3,297, 5,213)	0.45 (0.37, 0.53)
2003	28,462 (25,709, 31,199)	16,588 (13,673, 19,832)	11,867 (9,172, 14,180)	0.42 (0.33, 0.50)
2004	8,360 (6,806, 9,758)	3,905 (2,747, 5,243)	4,379 (3,350, 5,400)	0.53 (0.42, 0.62)
2005	10,108 (8,516, 11,501)	5,127 (3,891, 6,517)	4,954 (3,837, 5,922)	0.49 (0.39, 0.58)
2006	20,239 (18,003, 22,179)	11,094 (8,680, 13,693)	8,968 (6,830, 11,061)	0.45 (0.35, 0.55)
2007	12,225 (10,272, 14,003)	6,830 (5,287, 8,600)	5,346 (3,988, 6,571)	0.44 (0.33, 0.52)
2008	9,036 (7,244, 10,848)	4,210 (2,919, 5,703)	4,788 (3,674, 5,927)	0.53 (0.43, 0.62)
2009	10,673 (8,757, 12,515)	4,841 (3,326, 6,498)	5,759 (4,391, 6,984)	0.55 (0.44, 0.64)
2010	15,360 (13,248, 17,167)	8,342 (6,515, 10,428)	6,878 (5,331, 8,373)	0.45 (0.36, 0.54)
2011	9,082 (7,537, 10,607)	4,380 (3,133, 5,767)	4,682 (3,605, 5,698)	0.52 (0.41, 0.61)
2012	18,063 (15,622, 20,273)	9,467 (7,136, 11,985)	8,618 (6,589, 10,381)	0.48 (0.37, 0.57)
2013	14,812 (12,839, 16,584)	7,482 (5,763, 9,459)	7,252 (5,704, 8,693)	0.50 (0.39, 0.58)
2014	8,497 (6,978, 10,005)	3,631 (2,580, 4,903)	4,791 (3,758, 5,870)	0.57 (0.47, 0.66)
2015	23,673 (20,946, 25,841)	11,101 (8,420, 14,457)	12,537 (9,361, 15,049)	0.53 (0.40, 0.63)
2016	12,791 (10,709, 14,704)	4,960 (3,484, 6,813)	7,701 (6,065, 9,255)	0.61 (0.50, 0.70)
2017	18,286 (16,023, 20,382)	7,305 (5,305, 9,730)	10,961 (8,639, 12,944)	0.60 (0.49, 0.69)
2018	21,354 (18,874, 23,657)	8,562 (6,308, 11,391)	12,717 (10,128, 15,056)	0.60 (0.48, 0.69)
2019	22,601 (20,028, 25,121)	9,376 (7,080, 12,456)	13,139 (10,566, 15,477)	0.59 (0.47, 0.67)
2020	17,810 (15,688, 19,940)	7,381 (5,458, 9,752)	10,421 (8,275, 12,431)	0.59 (0.47, 0.68)
2021	16,512 (14,278, 18,655)	7,028 (5,156, 9,411)	9,409 (7,358, 11,182)	0.57 (0.46, 0.66)
2022	29,480 (26,643, 32,006)	12,116 (9,121, 15,963)	17,215 (13,570, 20,173)	0.59 (0.46, 0.68)
2023	21,958 (19,439, 24,245)	7,995 (5,734, 11,052)	13,846 (10,865, 16,218)	0.64 (0.51, 0.73)
2024	26,005 (22,858, 28,737)	8,860 (6,003, 12,810)	17,111 (13,188, 20,055)	0.66 (0.52, 0.76)
2025	24,572 (22,064, 26,867)	7,760 (5,385, 11,004)	16,768 (13,620, 19,321)	0.68 (0.56, 0.77)
**Average**	**16,553 (14,482, 18,518)**	**7,516 (5,724, 9,733)**	**8,986 (7,099, 10,561)**	**0.55 (0.44, 0.63)**

Median and 95% empirical confidence intervals of total number of expected heat-deaths in the factual and counterfactual scenarios, and the absolute and relative changes due to human-made climate change across 854 European cities, per year from 2000–2025.

**Extended Data Table 2:** Mean annual modelled heat-related mortality and number and proportion of deaths attributable to anthropogenic warming per year from 2000-2025 in 30 European capitals

Capital	Population	Heat deaths	Standardised excess	Difference	Proportion
Amsterdam	616,727	35 (23, 60)	74 (50, 127)	13 (8, 18)	0.35 (0.18, 0.56)
Athens	2,269,492	374 (270, 486)	161 (116, 209)	180 (120, 248)	0.51 (0.34, 0.66)
Berlin	2,805,374	284 (226, 360)	107 (85, 135)	146 (103, 191)	0.53 (0.35, 0.69)
Bern	176,221	21 (14, 30)	111 (75, 161)	11 (6, 16)	0.50 (0.27, 0.75)
Bratislava	337,865	29 (17, 46)	113 (66, 177)	18 (10, 28)	0.65 (0.42, 0.81)
Brussels	808,139	55 (41, 73)	81 (61, 108)	24 (15, 34)	0.45 (0.27, 0.62)
Bucharest	1,665,377	359 (268, 484)	268 (200, 360)	213 (131, 300)	0.64 (0.41, 0.79)
Budapest	1,414,149	157 (98, 246)	117 (73, 183)	91 (54, 137)	0.60 (0.40, 0.75)
Copenhagen	415,428	14 (9, 22)	42 (25, 63)	8 (4, 13)	0.58 (0.36, 0.75)
Dublin	893,653	6 (2, 10)	10 (4, 18)	3 (1, 7)	0.76 (0.28, 0.95)
Helsinki	453,721	19 (11, 27)	52 (31, 74)	10 (3, 17)	0.63 (0.27, 0.84)
Lisbon	1,425,616	117 (92, 156)	78 (61, 103)	38 (20, 56)	0.35 (0.20, 0.49)
Ljubljana	217,685	36 (25, 47)	177 (120, 232)	20 (13, 28)	0.56 (0.42, 0.69)
London	5,894,656	187 (123, 250)	47 (31, 62)	101 (57, 153)	0.62 (0.39, 0.79)
Luxembourg	70,074	4 (3, 6)	74 (53, 102)	2 (1, 3)	0.50 (0.32, 0.72)
Madrid	2,871,466	307 (220, 434)	108 (77, 153)	227 (152, 308)	0.73 (0.51, 0.87)
Nicosia	171,474	21 (13, 28)	166 (103, 218)	11 (3, 18)	0.55 (0.14, 0.80)
Oslo	435,270	10 (2, 17)	33 (8, 58)	5 (1, 10)	0.63 (0.20, 0.84)
Paris	6,869,559	328 (244, 454)	54 (40, 73)	177 (121, 243)	0.57 (0.36, 0.75)
Prague	990,604	62 (38, 96)	72 (44, 110)	39 (23, 59)	0.67 (0.50, 0.80)
Riga	550,511	72 (14, 133)	131 (24, 240)	28 (2, 65)	0.45 (0.14, 0.70)
Rome	2,158,892	577 (471, 679)	241 (197, 283)	350 (252, 437)	0.61 (0.45, 0.73)
Sofia	950,740	121 (74, 186)	156 (96, 241)	75 (42, 117)	0.65 (0.44, 0.82)
Stockholm	1,142,160	37 (24, 51)	39 (25, 54)	23 (12, 36)	0.68 (0.38, Inf)
Tallinn	316,478	18 (7, 32)	64 (25, 112)	8 (2, 16)	0.51 (0.26, 0.73)
Valletta	170,361	31 (23, 40)	226 (163, 288)	15 (-3, 26)	0.52 (-0.11, 0.81)
Vienna	1,315,748	139 (102, 187)	120 (89, 162)	86 (56, 121)	0.64 (0.43, 0.79)
Vilnius	429,750	52 (5, 107)	138 (16, 279)	19 (-1, 44)	0.41 (0.09, 0.69)
Warsaw	1,381,256	145 (92, 203)	105 (66, 147)	85 (52, 122)	0.60 (0.44, 0.74)
Zagreb	630,723	181 (128, 230)	314 (222, 400)	90 (60, 123)	0.51 (0.37, 0.64)

Population and median and 95% empirical confidence intervals of absolute and age-standardised mean annual expected heat-deaths in the factual scenario, and the absolute and relative changes due to human-made climate change from 2000–2025 in the 30 capitals.

**Extended Data Table 3:** As Extended Data Table 2, but for 2003 only

Capital	Population	Heat deaths	Standardised excess	Difference	Proportion
Amsterdam	616,727	43 (30, 70)	91 (65, 145)	14 (9, 19)	0.32 (0.17, 0.49)
Athens	2,269,492	265 (162, 383)	114 (70, 166)	119 (77, 174)	0.46 (0.31, 0.61)
Berlin	2,805,374	397 (315, 477)	149 (119, 179)	187 (132, 240)	0.47 (0.33, 0.59)
Bern	176,221	57 (41, 70)	299 (217, 368)	23 (13, 33)	0.40 (0.24, 0.55)
Bratislava	337,865	36 (20, 58)	141 (81, 225)	21 (11, 32)	0.59 (0.35, 0.77)
Brussels	808,139	89 (68, 111)	132 (102, 165)	28 (15, 43)	0.32 (0.18, 0.49)
Bucharest	1,665,377	314 (230, 457)	234 (171, 339)	170 (106, 242)	0.54 (0.34, 0.70)
Budapest	1,414,149	153 (85, 267)	114 (63, 199)	84 (49, 129)	0.56 (0.37, 0.72)
Copenhagen	415,428	15 (9, 24)	45 (26, 69)	8 (4, 13)	0.51 (0.34, 0.67)
Dublin	893,653	14 (5, 22)	23 (10, 38)	6 (2, 11)	0.46 (0.18, 0.68)
Helsinki	453,721	33 (20, 47)	89 (55, 127)	13 (4, 25)	0.41 (0.12, 0.65)
Lisbon	1,425,616	249 (215, 290)	165 (142, 192)	48 (27, 69)	0.19 (0.11, 0.27)
Ljubljana	217,685	67 (50, 85)	330 (244, 414)	27 (18, 37)	0.40 (0.28, 0.53)
London	5,894,656	421 (293, 533)	105 (73, 133)	148 (89, 217)	0.36 (0.23, 0.50)
Luxembourg	70,074	11 (9, 14)	204 (159, 241)	4 (2, 5)	0.31 (0.22, 0.43)
Madrid	2,871,466	385 (282, 522)	135 (98, 184)	233 (157, 314)	0.61 (0.45, 0.75)
Nicosia	171,474	16 (8, 24)	128 (65, 189)	8 (2, 16)	0.56 (0.14, 0.83)
Oslo	435,270	9 (2, 16)	29 (6, 55)	4 (1, 8)	0.50 (0.26, 0.74)
Paris	6,869,559	979 (788, 1,164)	161 (130, 192)	298 (210, 401)	0.30 (0.22, 0.41)
Prague	990,604	111 (67, 163)	129 (78, 188)	60 (36, 86)	0.54 (0.39, 0.67)
Riga	550,511	77 (15, 130)	138 (25, 233)	21 (1, 52)	0.28 (0.07, 0.59)
Rome	2,158,892	1,072 (929, 1,218)	446 (386, 507)	506 (361, 638)	0.47 (0.35, 0.60)
Sofia	950,740	98 (54, 174)	127 (69, 224)	58 (32, 94)	0.60 (0.38, 0.80)
Stockholm	1,142,160	60 (38, 83)	63 (40, 87)	34 (17, 55)	0.57 (0.31, 0.81)
Tallinn	316,478	26 (11, 42)	93 (39, 149)	8 (3, 17)	0.33 (0.14, 0.55)
Valletta	170,361	71 (54, 86)	513 (391, 627)	23 (-6, 45)	0.32 (-0.09, 0.61)
Vienna	1,315,748	194 (141, 262)	169 (122, 226)	106 (69, 147)	0.55 (0.37, 0.71)
Vilnius	429,750	37 (7, 78)	98 (18, 204)	13 (3, 28)	0.35 (0.19, 0.63)
Warsaw	1,381,256	94 (54, 147)	68 (39, 107)	57 (34, 83)	0.61 (0.42, 0.77)
Zagreb	630,723	286 (211, 352)	494 (365, 611)	103 (67, 144)	0.37 (0.24, 0.50)

Population and median and 95% empirical confidence intervals of absolute and age-standardised total number of expected heat-deaths in the factual scenario, and the absolute and relative changes due to human-made climate change in 30 capital cities during 2003.

**Extended Data Table 4:** As Extended Data Table 2, but for 2025 only

Capital	Population	Heat-deaths	Standardised excess	Difference	Proportion
Amsterdam	616,727	38 (26, 65)	80 (57, 134)	18 (12, 25)	0.48 (0.24, 0.71)
Athens	2,269,492	546 (421, 675)	235 (181, 291)	334 (231, 438)	0.62 (0.43, 0.77)
Berlin	2,805,374	225 (177, 301)	84 (67, 113)	144 (108, 181)	0.64 (0.45, 0.78)
Bern	176,221	38 (27, 48)	203 (142, 256)	25 (17, 35)	0.67 (0.46, 0.84)
Bratislava	337,865	27 (15, 44)	104 (59, 169)	22 (13, 35)	0.85 (0.61, 0.97)
Brussels	808,139	90 (68, 113)	134 (103, 169)	51 (34, 67)	0.56 (0.41, 0.72)
Bucharest	1,665,377	617 (474, 768)	460 (357, 572)	429 (274, 567)	0.71 (0.45, 0.86)
Budapest	1,414,149	210 (132, 317)	157 (99, 236)	156 (98, 227)	0.76 (0.55, 0.89)
Copenhagen	415,428	9 (6, 15)	28 (16, 44)	7 (4, 12)	0.79 (0.55, 0.93)
Dublin	893,653	15 (6, 25)	26 (11, 43)	10 (3, 18)	0.69 (0.30, 0.90)
Helsinki	453,721	28 (17, 41)	77 (47, 111)	21 (6, 35)	0.79 (0.19, 0.99)
Lisbon	1,425,616	176 (141, 225)	117 (94, 149)	82 (47, 113)	0.46 (0.29, 0.62)
Ljubljana	217,685	48 (35, 61)	236 (174, 298)	32 (23, 43)	0.68 (0.51, 0.80)
London	5,894,656	416 (274, 554)	104 (69, 138)	279 (167, 412)	0.69 (0.46, 0.84)
Luxembourg	70,074	6 (5, 8)	115 (86, 145)	4 (3, 5)	0.62 (0.47, 0.78)
Madrid	2,871,466	544 (399, 699)	190 (139, 246)	473 (333, 614)	0.88 (0.71, 0.97)
Nicosia	171,474	28 (19, 34)	219 (148, 272)	18 (5, 27)	0.66 (0.20, 0.89)
Oslo	435,270	22 (6, 36)	75 (21, 122)	14 (3, 26)	0.65 (0.25, 0.93)
Paris	6,869,559	542 (408, 703)	89 (67, 115)	389 (277, 511)	0.72 (0.55, 0.84)
Prague	990,604	54 (32, 85)	63 (38, 98)	44 (27, 66)	0.82 (0.66, 0.91)
Riga	550,511	52 (11, 103)	93 (20, 187)	32 (6, 77)	0.73 (0.23, 0.98)
Rome	2,158,892	910 (794, 1,033)	379 (331, 430)	666 (516, 789)	0.74 (0.58, 0.84)
Sofia	950,740	228 (151, 317)	297 (197, 411)	162 (94, 229)	0.72 (0.51, 0.87)
Stockholm	1,142,160	41 (25, 57)	43 (26, 60)	36 (19, 54)	0.93 (0.54, 1.00)
Tallinn	316,478	16 (7, 27)	59 (23, 95)	11 (4, 20)	0.71 (0.32, 0.96)
Valletta	170,361	43 (31, 54)	308 (225, 391)	27 (-19, 43)	0.63 (-0.46, 0.95)
Vienna	1,315,748	142 (104, 194)	123 (90, 167)	115 (80, 154)	0.82 (0.60, 0.94)
Vilnius	429,750	30 (4, 74)	80 (10, 194)	22 (3, 54)	0.77 (0.46, 0.97)
Warsaw	1,381,256	94 (58, 146)	68 (41, 105)	76 (47, 110)	0.82 (0.66, 0.92)
Zagreb	630,723	211 (150, 265)	365 (258, 462)	140 (98, 186)	0.68 (0.50, 0.82)

Population and median and 95% empirical confidence intervals of absolute and age-standardised total number of expected heat-deaths in the factual scenario, and the absolute and relative changes due to human-made climate change in 30 capital cities during 2025.

**Extended Data Table 5:** As Table 1, but using a variable mortality rate and population estimates

Country	Population	Heat-deaths	Standardised excess	Difference	Proportion
Austria	2,056,195	207 (156, 271)	116 (87, 152)	130 (88, 178)	0.63 (0.44, 0.78)
Belgium	2,592,559	190 (146, 245)	78 (60, 102)	85 (54, 118)	0.44 (0.29, 0.60)
Bulgaria	2,812,386	521 (346, 735)	221 (148, 310)	297 (182, 433)	0.60 (0.40, 0.74)
Croatia	1,038,972	397 (295, 490)	438 (325, 543)	196 (134, 262)	0.51 (0.37, 0.64)
Cyprus	401,527	34 (22, 44)	132 (86, 170)	19 (6, 30)	0.56 (0.16, 0.80)
Czechia	2,538,347	193 (118, 288)	94 (57, 138)	120 (70, 177)	0.63 (0.46, 0.76)
Denmark	1,451,136	45 (27, 66)	39 (24, 58)	24 (13, 39)	0.57 (0.35, 0.74)
Estonia	433,058	27 (10, 50)	74 (27, 134)	11 (3, 24)	0.49 (0.25, 0.71)
Finland	1,566,944	64 (28, 105)	49 (22, 80)	30 (10, 58)	0.52 (0.25, 0.76)
France	18,235,411	1,182 (955, 1,474)	67 (54, 84)	680 (505, 863)	0.56 (0.41, 0.70)
Germany	23,461,496	2,181 (1,750, 2,643)	107 (86, 129)	1,062 (753, 1,376)	0.49 (0.36, 0.62)
Greece	4,022,494	684 (499, 854)	180 (131, 224)	334 (226, 456)	0.50 (0.35, 0.64)
Hungary	2,794,665	378 (237, 558)	187 (119, 274)	206 (120, 303)	0.56 (0.37, 0.72)
Ireland	1,245,105	9 (4, 15)	12 (5, 19)	5 (1, 9)	0.66 (0.18, 0.89)
Italy	16,651,294	3,869 (3,170, 4,561)	213 (174, 251)	2,229 (1,516, 2,845)	0.57 (0.39, 0.69)
Latvia	763,497	166 (31, 312)	258 (52, 482)	62 (4, 141)	0.43 (0.13, 0.67)
Lithuania	892,253	102 (19, 199)	136 (28, 262)	39 (3, 85)	0.41 (0.14, 0.63)
Luxembourg	78,631	5 (4, 7)	82 (58, 112)	3 (2, 4)	0.49 (0.31, 0.72)
Malta	156,189	33 (24, 41)	264 (191, 335)	16 (-4, 28)	0.52 (-0.11, 0.81)
Netherlands	6,804,367	416 (267, 682)	73 (48, 119)	152 (96, 222)	0.35 (0.20, 0.54)
Norway	882,432	20 (8, 33)	32 (13, 51)	9 (3, 18)	0.55 (0.25, 0.76)
Poland	9,786,347	1,028 (667, 1,458)	130 (85, 184)	583 (368, 817)	0.57 (0.42, 0.70)
Portugal	3,491,884	314 (249, 407)	96 (77, 125)	113 (65, 162)	0.38 (0.23, 0.51)
Romania	5,127,898	961 (643, 1,320)	246 (166, 334)	543 (341, 773)	0.59 (0.40, 0.73)
Slovakia	879,013	101 (61, 149)	156 (96, 229)	61 (35, 89)	0.61 (0.43, 0.75)
Slovenia	314,357	54 (37, 70)	199 (138, 258)	29 (19, 41)	0.56 (0.39, 0.69)
Spain	18,398,828	2,262 (1,814, 2,872)	128 (103, 163)	1,384 (995, 1,777)	0.58 (0.43, 0.71)
Sweden	3,042,720	97 (62, 134)	35 (22, 49)	54 (27, 86)	0.59 (0.33, 0.78)
Switzerland	1,898,020	178 (129, 240)	99 (71, 135)	102 (69, 137)	0.55 (0.35, 0.75)
United Kingdom	27,004,332	650 (503, 796)	28 (22, 34)	354 (211, 501)	0.59 (0.36, 0.75)

Population and median and 95% empirical confidence intervals of absolute and age-standardised mean annual heat-related excess deaths in the factual scenario, and the absolute and relative changes due to human-made climate change from 2000–2025 across 854 European cities using variable mortality rates and population estimates, aggregated per country.

**Extended Data Table 6:** As Extended Data Table 1, but using a variable mortality rate and population estimates

Year	Factual	Counterfactual	Difference	Proportion
2000	8,591 (7,151, 9,910)	5,312 (4,068, 6,616)	3,248 (2,440, 3,978)	0.38 (0.30, 0.46)
2001	10,168 (8,388, 11,682)	5,698 (4,242, 7,205)	4,420 (3,441, 5,367)	0.44 (0.35, 0.52)
2002	8,717 (7,133, 10,214)	4,821 (3,588, 6,102)	3,848 (2,985, 4,689)	0.44 (0.36, 0.53)
2003	25,627 (23,086, 28,089)	15,098 (12,472, 18,116)	10,484 (8,142, 12,565)	0.41 (0.32, 0.49)
2004	7,740 (6,271, 9,083)	3,717 (2,623, 5,011)	3,987 (3,064, 4,921)	0.52 (0.41, 0.61)
2005	9,407 (7,937, 10,718)	4,853 (3,640, 6,144)	4,521 (3,521, 5,405)	0.48 (0.39, 0.57)
2006	19,080 (16,935, 20,904)	10,584 (8,222, 12,950)	8,372 (6,359, 10,300)	0.44 (0.34, 0.54)
2007	11,564 (9,741, 13,244)	6,522 (5,029, 8,215)	4,982 (3,702, 6,103)	0.43 (0.33, 0.51)
2008	8,697 (6,959, 10,428)	4,133 (2,842, 5,514)	4,529 (3,496, 5,631)	0.53 (0.42, 0.61)
2009	10,237 (8,455, 11,932)	4,743 (3,258, 6,342)	5,429 (4,153, 6,609)	0.54 (0.43, 0.63)
2010	14,926 (12,828, 16,713)	8,190 (6,384, 10,186)	6,596 (5,141, 8,043)	0.45 (0.35, 0.53)
2011	9,026 (7,386, 10,546)	4,423 (3,149, 5,795)	4,561 (3,541, 5,556)	0.51 (0.41, 0.60)
2012	17,917 (15,461, 20,007)	9,419 (7,106, 11,934)	8,445 (6,503, 10,207)	0.48 (0.37, 0.56)
2013	14,979 (12,934, 16,745)	7,586 (5,838, 9,569)	7,293 (5,740, 8,747)	0.49 (0.39, 0.58)
2014	8,723 (7,226, 10,190)	3,775 (2,669, 5,074)	4,860 (3,814, 5,951)	0.56 (0.47, 0.65)
2015	23,819 (21,136, 26,044)	11,145 (8,427, 14,491)	12,605 (9,415, 15,108)	0.53 (0.40, 0.63)
2016	13,079 (10,934, 15,029)	5,083 (3,568, 6,927)	7,865 (6,209, 9,451)	0.61 (0.50, 0.70)
2017	18,898 (16,551, 21,007)	7,525 (5,513, 10,031)	11,316 (8,898, 13,353)	0.60 (0.49, 0.69)
2018	21,950 (19,442, 24,267)	8,784 (6,500, 11,704)	13,056 (10,435, 15,445)	0.60 (0.48, 0.69)
2019	23,316 (20,714, 25,813)	9,640 (7,314, 12,723)	13,573 (10,908, 15,975)	0.59 (0.48, 0.67)
2020	18,561 (16,431, 20,661)	7,686 (5,689, 10,153)	10,862 (8,619, 12,928)	0.59 (0.48, 0.68)
2021	17,117 (14,840, 19,295)	7,320 (5,358, 9,707)	9,757 (7,663, 11,560)	0.57 (0.46, 0.66)
2022	30,815 (27,975, 33,482)	12,616 (9,526, 16,559)	18,090 (14,243, 21,199)	0.59 (0.47, 0.68)
2023	23,043 (20,461, 25,307)	8,350 (5,990, 11,448)	14,568 (11,461, 17,047)	0.64 (0.51, 0.73)
2024	27,135 (23,992, 29,989)	9,074 (6,152, 13,167)	17,994 (13,950, 20,975)	0.67 (0.53, 0.76)
2025	26,133 (23,540, 28,492)	8,141 (5,636, 11,511)	17,905 (14,646, 20,622)	0.69 (0.57, 0.77)
**Average**	**16,511 (14,469, 18,432)**	**7,472 (5,659, 9,699)**	**8,981 (7,090, 10,548)**	**0.55 (0.44, 0.63)**

Median and 95% empirical confidence intervals of total number of expected heat-deaths under the factual and counterfactual scenarios using a variable mortality rate and population, and the absolute and relative changes due to human-made climate change across 854 European cities, aggregated per year from 2000–2025.

**Extended Data Table 7:** As Table 1, but using a summer-specific mortality rate and variable population estimates

Country	Population	Heat-deaths	Standardised excess	Difference	Proportion
Austria	2,056,195	193 (145, 252)	108 (81, 141)	120 (81, 165)	0.63 (0.44, 0.78)
Belgium	2,592,559	174 (133, 226)	72 (55, 94)	77 (49, 108)	0.44 (0.28, 0.60)
Bulgaria	2,812,386	470 (312, 662)	199 (133, 279)	268 (164, 390)	0.59 (0.40, 0.74)
Switzerland	1,898,020	166 (119, 225)	92 (66, 126)	94 (63, 126)	0.55 (0.35, 0.75)
Cyprus	401,527	31 (20, 40)	119 (78, 154)	17 (5, 27)	0.56 (0.16, 0.80)
Czechia	2,538,347	178 (108, 267)	86 (53, 128)	111 (65, 163)	0.63 (0.46, 0.76)
Denmark	1,451,136	42 (25, 62)	37 (22, 54)	22 (12, 36)	0.57 (0.35, 0.74)
Estonia	433,058	25 (9, 46)	67 (24, 123)	10 (3, 22)	0.48 (0.25, 0.71)
Greece	4,022,494	691 (505, 863)	181 (132, 227)	337 (227, 459)	0.50 (0.35, 0.64)
Spain	18,398,828	2,092 (1,676, 2,659)	119 (95, 151)	1,276 (917, 1,640)	0.58 (0.43, 0.71)
Finland	1,566,944	59 (26, 98)	45 (20, 74)	28 (9, 54)	0.52 (0.25, 0.76)
France	18,235,411	1,077 (866, 1,353)	61 (49, 77)	615 (457, 782)	0.56 (0.40, 0.70)
Hungary	2,794,665	345 (216, 511)	170 (108, 249)	188 (109, 276)	0.56 (0.37, 0.72)
Italy	16,651,294	3,594 (2,946, 4,238)	198 (162, 233)	2,068 (1,405, 2,639)	0.57 (0.39, 0.69)
Lithuania	892,253	94 (17, 184)	125 (25, 241)	35 (3, 78)	0.41 (0.14, 0.63)
Luxembourg	78,631	5 (3, 7)	76 (54, 104)	2 (2, 3)	0.48 (0.31, 0.72)
Latvia	763,497	151 (28, 284)	233 (47, 436)	56 (3, 128)	0.42 (0.12, 0.67)
Netherlands	6,804,367	390 (248, 640)	68 (44, 111)	140 (88, 205)	0.34 (0.19, 0.53)
Norway	882,432	19 (8, 31)	30 (12, 47)	9 (3, 16)	0.55 (0.25, 0.76)
Poland	9,786,347	935 (606, 1,329)	118 (77, 167)	530 (333, 743)	0.57 (0.42, 0.70)
Portugal	3,491,884	280 (221, 363)	86 (68, 111)	100 (58, 144)	0.38 (0.23, 0.51)
Romania	5,127,898	886 (594, 1,218)	225 (153, 306)	500 (315, 712)	0.59 (0.40, 0.73)
Sweden	3,042,720	88 (56, 123)	32 (20, 45)	49 (25, 78)	0.59 (0.33, 0.78)
Slovakia	879,013	93 (57, 138)	144 (89, 211)	56 (32, 82)	0.61 (0.43, 0.75)
United Kingdom	27,004,332	597 (461, 734)	26 (20, 32)	322 (190, 457)	0.59 (0.36, 0.75)

Population and median and 95% empirical confidence intervals of absolute and age-standardised mean annual heat-related excess deaths in the factual scenario, and the absolute and relative changes due to human-made climate change from 2000–2025 across 854 European cities using summer-specific mortality rates and variable population, aggregated per country (where available).

**Extended Data Table 8:** List of climate models used in the attribution analysis

ACCESS-CM2 rlilplfl	ACCESS-ESM1-5 r1i1p1f1	AWI-ESM-1-REcoM r1i1p1f1
BCC-CSM2-MR rlilplfl	CAMS-CSM1-0 r1i1p1f1	CanESM5 r10i1p1f1
CanESM5-CanOE r1i1p2f1	CESM2 r10i1p1f1	CESM2-WACCM r1i1p1f1
CIESM rlilplfl	CMCC-CM2-SR5 r1i1p1f1	CMCC-ESM2 r1i1p1f1
CNRM-CM6-1-HR r1i1p1f2	CNRM-ESM2-1 r1i1p1f2	E3SM-1-1 r1i1p1f1
EC-Earth3-CC r1i1p1f1	FGOALS-f3-L r1i1p1f1	FIO-ESM-2-0 r1i1p1f1
GFDL-CM4 r1i1p1f1	GFDL-ESM4 r1i1p1f1	GISS-E2-1-G r1i1p1f2
HadGEM3-GC31-MM r1i1p1f3	IITM-ESM r1i1p1f1	INM-CM4-8 r1i1p1f1
INM-CM5-0 r1i1p1f1	IPSL-CM6A-LR r14i1p1f1	KACE-1-0-G r1i1p1f1
KIOST-ESM r1i1p1f1	MIROC-ES2L r1i1p1f2	MIROC6 r10i1p1f1
MPI-ESM1-2-LR r10i1p1f1	MRI-ESM2-0 r1i1p1f1	NESM3 r1i1p1f1
NorESM2-MM r1i1p1f1	TaiESM1 r1i1p1f1	UKESM1-0-LL r1i1p1f2

List of CMIP6 climate models and realisations used in the attribution analysis.

## Supplementary Material

Supplementary information The online version contains supplementary material available at https://doi.org/10.1038/s44360-026-00209-8.

Appendix

Peer Review File

Supplementary information

Reporting Summary

## Figures and Tables

**Table 1:** Population size, medians and 95% eCIs of the total number of expected heat deaths from 2000 to 2025 in 854 European cities under the factual scenario and the absolute and relative changes due to human-made climate change, aggregated per age group

Age	Population	Heat deaths	Difference	Proportion
20–44	66,886,597	12,116 (8,180–16,170)	4,321 (2,950–5,814)	0.36 (0.27–0.47)
45–64	54,059,644	58,970 (47,789–71,317)	28,056 (21,611–34,446)	0.48 (0.38–0.57)
65–74	19,676,737	68,881 (58,766–78,784)	36,607 (28,837–43,785)	0.53 (0.43–0.62)
75–84	12,762,858	123,455 (107,344–138,259)	68,562 (54,277–80,954)	0.56 (0.45–0.64)
85+	5,087,812	165,992 (147,573–185,609)	95,583 (75,355–112,112)	0.58 (0.47–0.66)
Total	158,473,649	430,365 (376,535–481,458)	233,626 (184,565–274,584)	0.55 (0.44–0.63)

**Table 2:** Population size, medians and 95% eCIs of absolute and age-standardized mean annual heat-related excess deaths in the factual scenario and the absolute and relative changes due to human-made climate change from 2000 to 2025 in 854 European cities, aggregated per country

Country	Population	Heat deaths	Standardized excess	Difference	Proportion
Austria	1,949,160	192 (144–251)	109 (82–142)	120 (81–165)	0.64 (0.45–0.78)
Belgium	2,455,390	177 (137–229)	73 (56–95)	80 (51–111)	0.45 (0.29–0.60)
Bulgaria	2,596,877	498 (334–700)	213 (144–299)	284 (175–413)	0.60 (0.40–0.74)
Croatia	1,101,531	337 (253–414)	327 (245–402)	170 (117–227)	0.52 (0.38–0.64)
Cyprus	457,113	50 (33–64)	147 (98–189)	27 (8–42)	0.57 (0.17–0.80)
Czechia	2,522,881	193 (118–288)	89 (54–132)	119 (70–175)	0.63 (0.46–0.76)
Denmark	917,663	28 (17–41)	35 (21–49)	15 (8–24)	0.57 (0.35–0.75)
Estonia	440,155	27 (10–49)	65 (24–117)	11 (3–23)	0.48 (0.25–0.71)
Finland	1,460,602	63 (29–103)	46 (21–75)	30 (10–57)	0.52 (0.25–0.76)
France	17,962,199	1,146 (926–1,387)	61 (49–74)	659 (488–836)	0.58 (0.43–0.71)
Germany	23,406,685	2,249 (1,806–2,722)	93 (74–113)	1,099 (779–1,424)	0.49 (0.36–0.62)
Greece	3,401,064	618 (459–771)	171 (127–214)	297 (199–407)	0.50 (0.34–0.64)
Hungary	2,806,295	393 (247–575)	152 (96–221)	217 (127–316)	0.56 (0.37–0.72)
Ireland	1,090,316	8 (3–13)	11 (4–17)	4 (1–8)	0.67 (0.19–0.90)
Italy	17,186,805	3,947 (3,227–4,653)	197 (161–233)	2,256 (1,536–2,884)	0.57 (0.40–0.70)
Latvia	874,434	116 (24–215)	131 (27–244)	44 (4–98)	0.42 (0.14–0.66)
Lithuania	1,033,748	129 (26–250)	128 (26–247)	49 (4–106)	0.41 (0.16–0.63)
Luxembourg	70,074	4 (3–6)	74 (53–102)	2 (1–3)	0.50 (0.32–0.72)
Malta	170,361	31 (23–40)	226 (163–288)	15 (–3 to 26)	0.52 (–0.11–0.81)
the Netherlands	5,644,325	368 (235–606)	70 (46–115)	132 (83–194)	0.35 (0.20–0.54)
Norway	829,551	20 (9–31)	32 (14–49)	9 (3–17)	0.54 (0.26–0.76)
Poland	10,396,899	1,084 (704–1,540)	117 (76–165)	608 (382–853)	0.57 (0.42–0.70)
Portugal	3,457,781	324 (259–418)	91 (72–117)	116 (67–167)	0.38 (0.23–0.52)
Romania	6,010,799	1,168 (778–1,602)	223 (150–305)	660 (416–940)	0.59 (0.40–0.73)
Slovakia	877,322	94 (57–139)	145 (89–212)	56 (32–82)	0.61 (0.43–0.76)
Slovenia	308,320	55 (38–72)	190 (131–247)	30 (19–42)	0.56 (0.39–0.69)
Spain	17,714,878	2,212 (1,772–2,806)	118 (94–150)	1,335 (956–1,719)	0.58 (0.43–0.71)
Sweden	2,807,484	93 (59–129)	35 (22–48)	52 (26–83)	0.59 (0.33–0.78)
Switzerland	1,977,029	174 (126–237)	90 (65–123)	100 (67–134)	0.56 (0.36–0.76)
UK	26,545,907	625 (483–767)	27 (21–33)	338 (200–481)	0.59 (0.36–0.75)

## Data Availability

The datasets used in this study are open access. Temperature data can be downloaded from ERA5-Land (https://cds.climate.copernicus.eu/datasets/reanalysis-era5-land?tab=overview) and E-OBS (https://cds.climate.copernicus.eu/datasets/insitu-gridded-observations-europe?tab=overview), the annual GMST from NASA (https://data.giss.nasa.gov/gistemp/index_v3.html) and the multimodel ensemble of coupled global circulation models from the CMIP6 experiments (https://cds.climate.copernicus.eu/datasets/projections-cmip6?tab=download). Selected data used in the attribution step are available via Zenodo at https://doi.org/10.5281/zenodo.22228326 (ref. 47). The exposure response functions at the 854 cities are available via Zenodo at https://doi.org/10.5281/zenodo.7672107 (ref. 48), mortality data are available from eurostat (https://ec.europa.eu/eurostat/data/database), and population data are available from WorldPop (https://www.worldpop.org/).

## Code availability

Preprocessing of weather and climate data was performed using Python (version 3.11.7). Statistical modelling was performed in R (version 4.5.3). Code used in the attribution step is available on Zenodo at https://doi.org/10.5281/zenodo.22228326, and code for performing the health impact assessment is provided on GitHub at https://github.com/PierreMasselot/Paper--2023--LancetPH--EUcityTRM.
