# Supplementary material for: Heat-related urban mortality attributable to human-induced climate change in Europe from 2000 to 2025
Source: Nat Health. Author manuscript; Available in PMC 2026 Oct 29. (PMC7619643; doi:10.1038/s44220-026-00674-w)
Supplement: Supplementary file 1 [file EMS221703-supplement-Appendix.pdf]

nature health

Supplementary information https://doi.org/10.1038/s44360-026-00209-8

Heat-related urban mortality attributable to human-induced climate change in Europe from 2000 to 2025

In the format provided by the authors and unedited

Contents

S1 Sensitivity to updated Population and Mortality Rates **2**

S2 Underestimation of European warming trends in climate models **12**

S1 Sensitivity to updated Population and Mortality Rates

In a sensitivity analysis, we derived city-specific annual mortality estimates for 2000–2025. Briefly, we combined all-cause mortality data at the third level of the Nomenclature of Territorial Units for Statistics (NUTS3) with gridded population data to estimate age-specific mortality rates in each NUTS3 region. We then assumed that each city (URAU specification) shared the mortality rate of its corresponding NUTS3 region and applied this rate to city-level population counts to estimate the annual number of deaths by age group and city.

We retrieved weekly all-cause mortality data from Eurostat, aggregated by NUTS3 region and five-year age groups (0–4, 5–9, . . . , 85–89, 90+). For Germany, Croatia, Ireland, Malta and Slovenia, mortality data were only available at annual resolution, Table S1. Data availability varied across countries: some had long and complete time series, whereas others had more limited temporal coverage, Table S1. We aggregated the available data into age groups consistent with the main analysis, namely 20–44, 45–64, 65–74, 75–84 and 85+ years, and then summed deaths to the annual level for all available years.

The NUTS3 classification provided in the dataset corresponds to the year to which the data refer. To ensure consistent spatial boundaries over time, we harmonised all data to the most recent available NUTS3 classification, which was 2024 for most countries and 2021 for the UK. We retained the NUTS3 regions containing the 854 study cities and achieved an almost exact match between the 2024 and historical NUTS3 regions in all countries except Portugal. In Portugal, the Área Metropolitana de Lisboa was split in the 2024 NUTS classification into two regions, Grande Lisboa and Península de Setúbal. To preserve consistent boundaries over time, we aggregated these two regions into a single unit.

In a small number of NUTS3 regions, the total number of deaths recorded in one year was fewer than 5% of the next-smallest year. For these specific NUTS3 regions, the outlying year of data was removed as errors. Additionally, for NUTS3 regions where the total number of deaths recorded in a year was at least twice that of the next largest year, we also removed these data as errors.

We obtained population estimates for the study period from WorldPop (https://www.worldpop.org/). Our study period spans two WorldPop products: the WorldPop 'Global 1' estimates [1] for 2000–2014 and the 'Global 2' estimates [2] for 2015–2025, both at a spatial resolution of 30 arc-seconds (approximately 1 km at the equator). Population counts in both products are provided in five-year age groups, although the definition of the oldest age group differs between products. In 'Global 1', the oldest age group is 80+, whereas in 'Global 2' the oldest is 90+. To obtain population estimates consistent with the age groups used in our study, we aggregated the original five-year age groups into the analysis groups. For older ages, we estimated the proportion of individuals aged 80+ who were aged 85+ using the 2015 WorldPop estimates, and applied this proportion to split the pre-2015 80+ population into 80–84 and 85+ groups. We then aggregated the gridded population estimates to both NUTS3 regions and URAU city units (hereafter referred to as cities).

For each NUTS3 region in which the centroid of a city was located, we estimated the annual age-specific mortality rate by dividing the all-cause mortality counts retrieved from Eurostat by the corresponding NUTS3 population totals, Fig. S1. To infer mortality rates for years without Eurostat data, we fitted a mixed-effects regression model to the NUTS3 regions included in the analysis. For each country, annual mortality trends were modelled using a linear term, with random slopes for the NUTS3 regions containing our 854 cities to allow for region-specific deviations. This partial pooling improved the stability of slope

estimates, particularly in regions with limited temporal coverage and small numbers. For countries with only one NUTS3 region of interest, we used a fixed slope. The resulting annual mortality-rate estimates for each NUTS3 region were then used as proxies for the mortality rates of cities within that region and applied to city population counts to estimate the annual number of deaths at the city level.

The results of this approach are shown in Figs. S2–S3. Fig. S2 compares the annual mortality rates observed in the 30 capitals (black points) with the constant rate used by Masselot et al. [3] (blue line) and the rate predicted by our mixed model (red line). The two approaches were generally consistent, with larger differences mainly in areas with sparse data or outlying observations. Fig. S3 compares the estimated number of deaths in a random sample of 30 cities across the five age groups with the constant mortality counts used by Masselot et al. [3]. These estimates were also broadly consistent, with differences usually below 15%. Some spatial heterogeneity was apparent, especially in cities with smaller death counts, and discrepancies were greatest at the beginning and end of the study period.

Table S1: **Mortality data availability.** Description of data availability for mortality data estimates (All the data not mentioned here are available for weekly data across all age groups, NUTS3 regions, and years from 2000 to 2025).

Country	NUTS3 ID	Age group	Years available	Temporal resolution	Source
Croatia (HR)	HR024, HR025, HR050	All groups	2020-2024	annual	[4]
Croatia (HR)	HR031, HR033, HR035, HR036	All groups	2013-2024	annual	[4]
Cyprus (CY)	All regions	All groups	2014-2025	weekly	[5]
Czechia (CZ)	All regions	All groups	2005-2025	weekly	[5]
Denmark (DK)	All regions	All groups	2007-2025	weekly	[5]
Estonia (EE)	EE00A	All groups	2019-2024	weekly	[5]
Estonia (EE)	EE001, EE008	All groups	2000-2024	weekly	[5]
Finland (FI)	All regions	All groups	2000-2022, 2024-2025	weekly	[5]
France (FR)	All regions	All groups	2012-2025	weekly	[5]
Germany (DE)	DEB31	20-44	2013-2017	annual	[4]
Germany (DE)	See Note 1	20-44	2013-2017, 2023	annual	[4]
Germany (DE)	See Note 2	20-44	2013-2017, 2023-2024	annual	[4]
Germany (DE)	DE222	45-64	2013-2017, 2020-2023	annual	[4]
Germany (DE)	See Note 3	45-64, 65-74, 75-84, 85+	2013-2017, 2020-2024	annual	[4]
Greece (EL)	All regions	All groups	2014-2025	weekly	[5]
Ireland (IE)	All regions	All groups	2013-2024	annual	[4]
Italy (IT)	All regions	All groups	2011-2025	weekly	[5]

Table S1: Description of data availability for mortality data estimates (All the data not mentioned here are available for weekly data across all age groups, NUTS3 regions, and years from 2000 to 2025). *(continued)*

Country	NUTS3 ID	Age group	Years available	Temporal resolution	Source
Lithuania (LT)	All regions	All groups	2010-2025	weekly	[5]
Malta (MT)	All regions	All groups	2013-2024	annual	[4]
Netherlands (NL)	All regions	All groups	2014-2025	weekly	[5]
Romania (RO)	All regions	All groups	2014-2025	weekly	[5]
Slovenia (SI)	All regions	All groups	2013-2024	annual	[4]
United Kingdom (UK)	All regions	All groups	2014-2020	weekly	[5]

Note:

1: DE112, DE117, DE122, DE125, DE129, DE131, DE141, DE211, DE221, DE222, DE232, DE242, DE252, DE253, DE261, DE262, DE263, DE273, DE279, DE401, DE402, DE403, DE404, DE501, DE502, DE711, DE712, DE713, DE722, DE731, DE803, DE804, DE80J, DE911, DE912, DE913, DE925, DE931, DE935, DE944, DE945, DEA11, DEA13, DEA17, DEA18, DEA19, DEA24, DEA2B, DEA31, DEA32, DEA51, DEA54, DEA56, DEB21, DEB32, DEB38, DED2D, DED44, DED45, DEE01, DEF01, DEF02, DEF04, DEG01, DEG02, DEG05

2: DE111, DE113, DE115, DE126, DE134, DE136, DE138, DE142, DE144, DE147, DE212, DE213, DE241, DE254, DE271, DE300, DE600, DE714, DE719, DE721, DE724, DE732, DE80L, DE80N, DE91C, DE929, DE943, DEA12, DEA14, DEA15, DEA16, DEA1A, DEA1D, DEA1F, DEA22, DEA23, DEA26, DEA2C, DEA2D, DEA33, DEA34, DEA36, DEA41, DEA47, DEA52, DEA53, DEA55, DEA58, DEA5A, DEB11, DEB34, DEB35, DED21, DED41, DED51, DEE02, DEE03, DEF03, DEG03

3: DE111, DE112, DE113, DE115, DE117, DE122, DE125, DE126, DE129, DE131, DE134, DE136, DE138, DE141, DE142, DE144, DE147, DE211, DE212, DE213, DE221, DE222, DE232, DE241, DE242, DE252, DE253, DE254, DE261, DE262, DE263, DE271, DE273, DE279, DE300, DE401, DE402, DE403, DE404, DE501, DE502, DE600, DE711, DE712, DE713, DE714, DE719, DE721, DE722, DE724, DE731, DE732, DE803, DE804, DE80J, DE80L, DE80N, DE911, DE912, DE913, DE91C, DE925, DE929, DE931, DE935, DE943, DE944, DE945, DEA11, DEA12, DEA13, DEA14, DEA15, DEA16, DEA17, DEA18, DEA19, DEA1A, DEA1D, DEA1F, DEA22, DEA23, DEA24, DEA26, DEA2B, DEA2C, DEA2D, DEA31, DEA32, DEA33, DEA34, DEA36, DEA41, DEA47, DEA51, DEA52, DEA53, DEA54, DEA55, DEA56, DEA58, DEA5A, DEB11, DEB21, DEB31, DEB32, DEB34, DEB35, DEB38, DED21, DED2D, DED41, DED44, DED45, DED51, DEE01, DEE02, DEE03, DEF01, DEF02, DEF03, DEF04, DEG01, DEG02, DEG03, DEG05

Fig. S1: **Comparison of URAU and NUTS3 regions.** Map of URAU city boundaries (red) and corresponding NUTS3 regions (blue) for the 30 European capitals, linked based on city centroid locations (black stars).

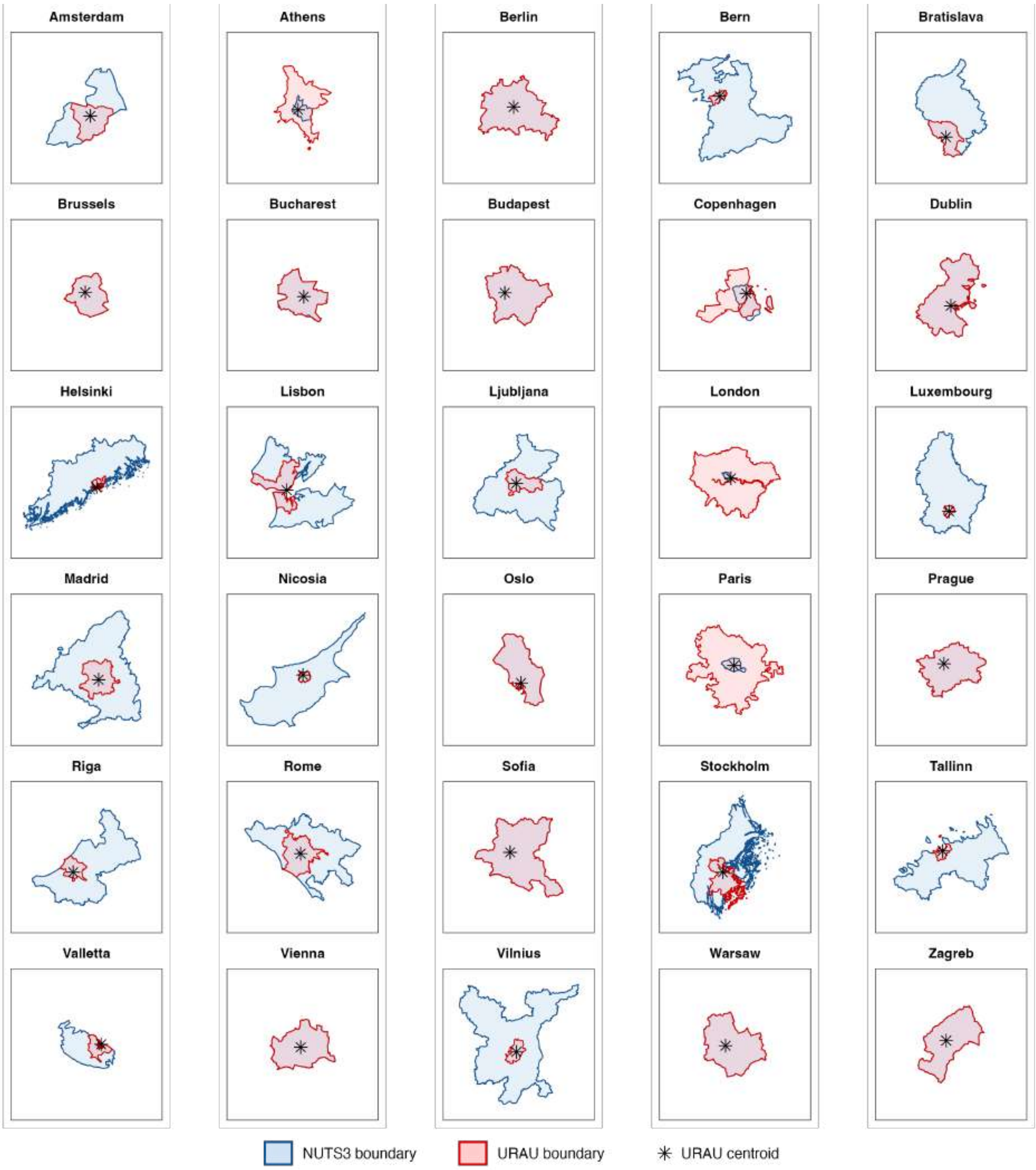

Fig. S2: **Comparison of** Time trends in annual mortality rates (2000-2025) by age group and shown for the 30 capital cities. Displayed are the mortality rates used in [3] (blue) and those estimated for our sensitivity analysis (red). The data used to estimate the mortality rates in our sensitivity analysis are also displayed (black).

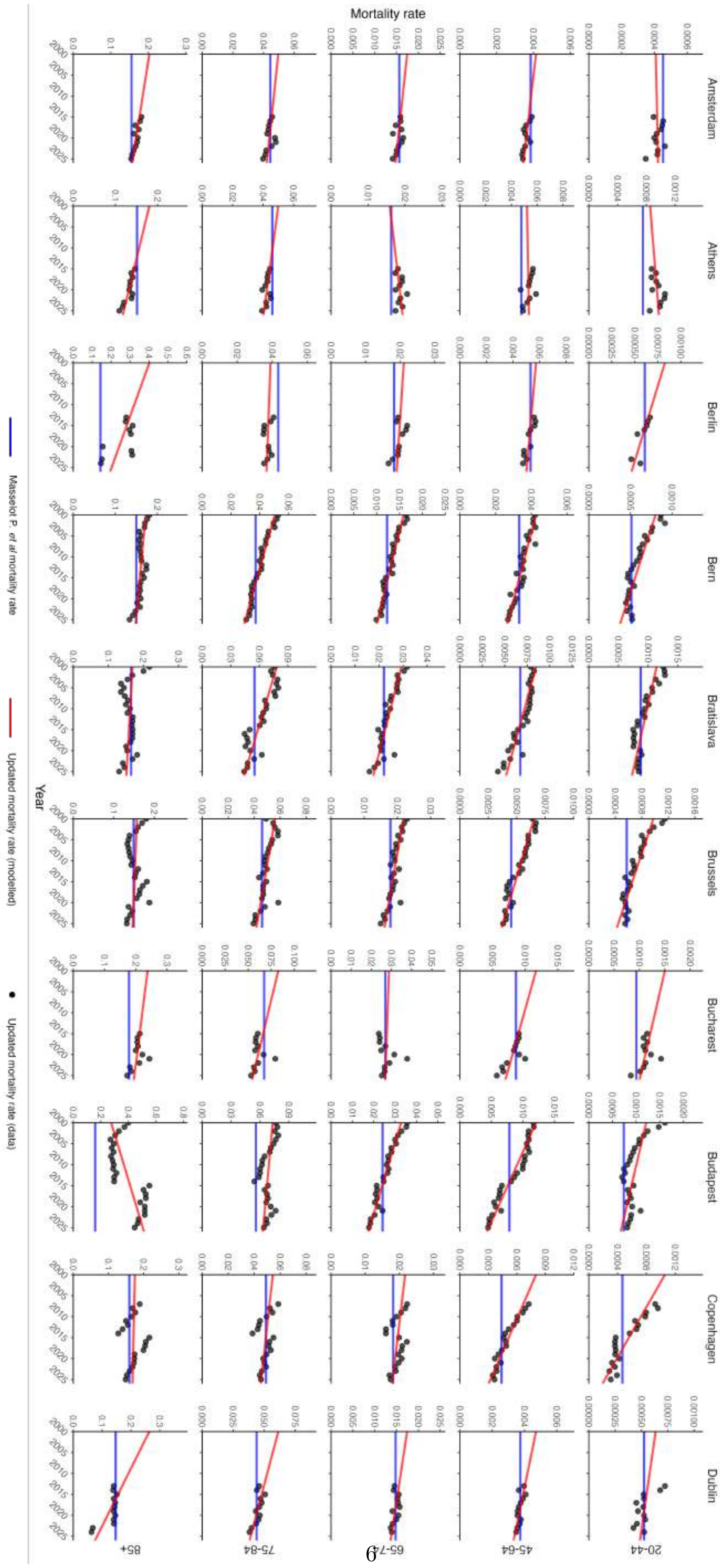

Fig. S2

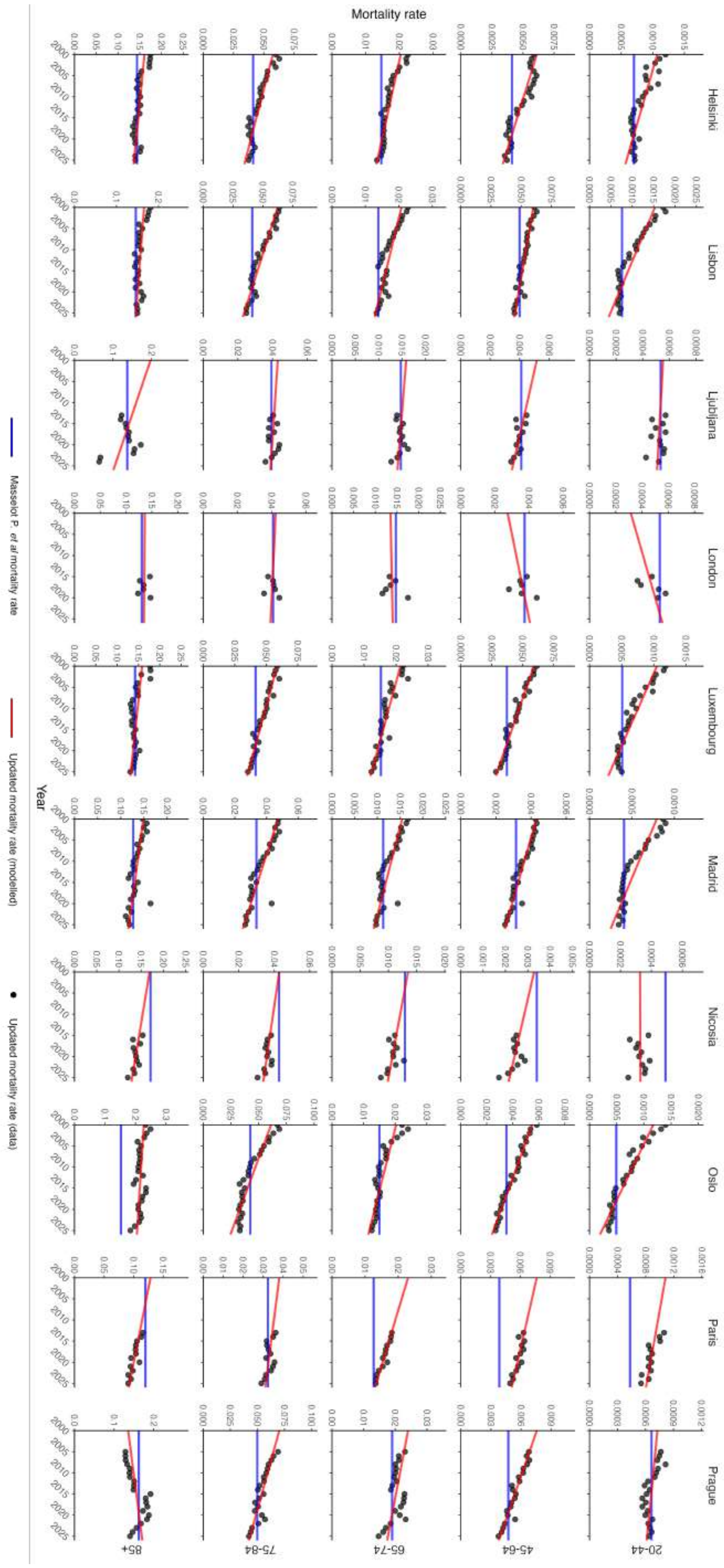
Mortality rate
Year
Helsinki
Lisbon
Ljubljana
London
Luxembourg
Madrid
Nicosia
Oslo
Paris
Prague
20-44
45-64
65-74
75-84
85+
Masselot P. et al mortality rate
Updated mortality rate (modelled)
Updated mortality rate (data)

Fig. S2

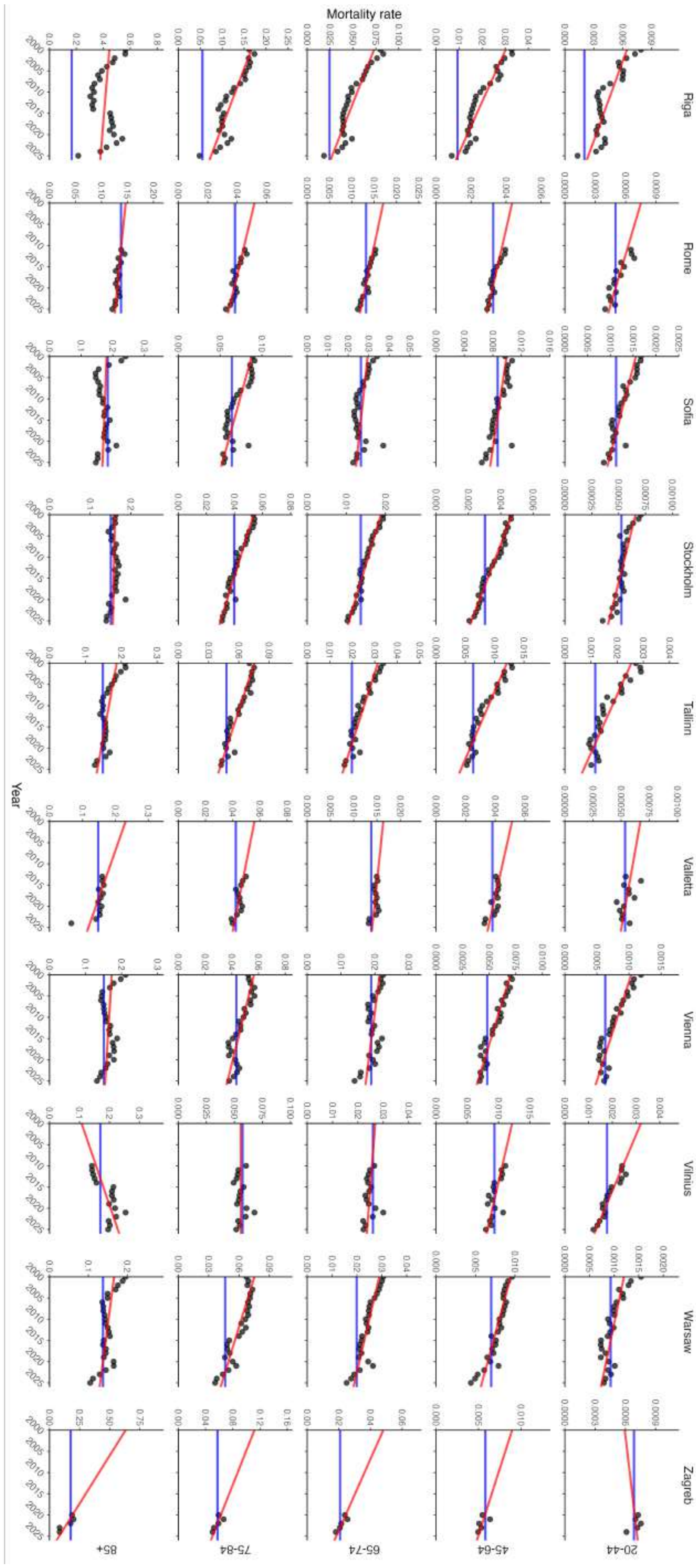

Riga
Rome
Sofia
Stockholm
Tallinn
Valletta
Vienna
Vilnius
Warsaw
Zagreb
20-44
45-64
65-74
75-84
85+
Mortality rate
Year
Masselot P. et al mortality rate
Updated mortality rate (modelled)
Updated mortality rate (data)

Fig. S3: Comparison between the number of annual city wide deaths (2000-2026) as used in [3] and as estimated for use in our sensitivity analysis, by age group and for a random subset of 30 cities. Here, data above the solid line indicate a higher mortality rate used in [3], and data below the solid line indicate a larger mortality rate used in our sensitivity analysis, where the dashed lines indicate a 5% and 10% magnitude of difference. The colour gradation indicates the year from 2000 (dark blue) to 2026 (light blue).

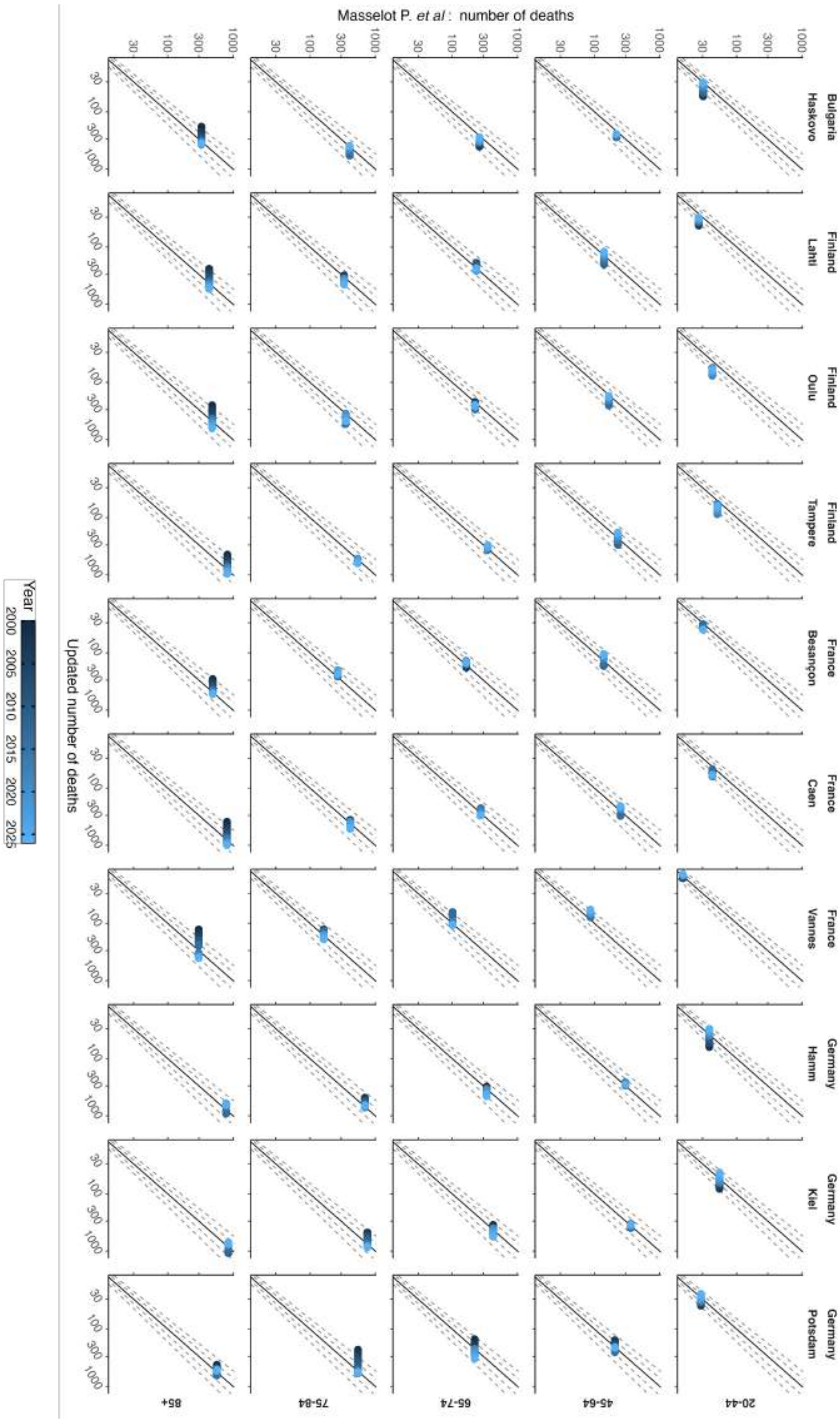

Fig. S3

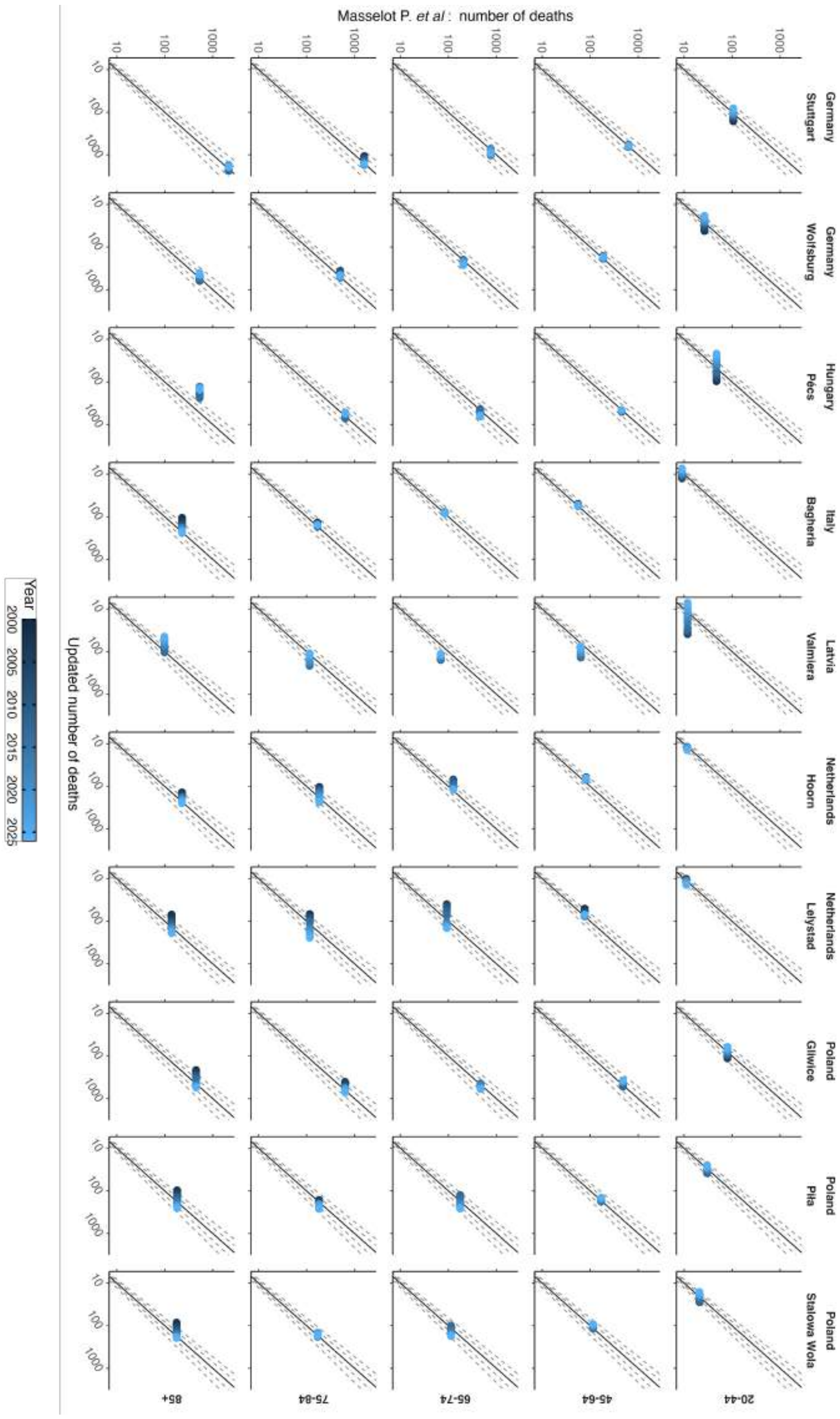
Masselot P. et al : number of deaths
Updated number of deaths
Germany Stuttgart
Germany Wolfsburg
Hungary Pécs
Italy Bagheria
Latvia Valmiera
Netherlands Hoorn
Netherlands Lelystad
Poland Gliwice
Poland Piła
Poland Stalowa Wola
20-44
45-64
65-74
75-84
85+
Year
2000
2005
2010
2015
2020
2025
10
100
1000

Fig. S3

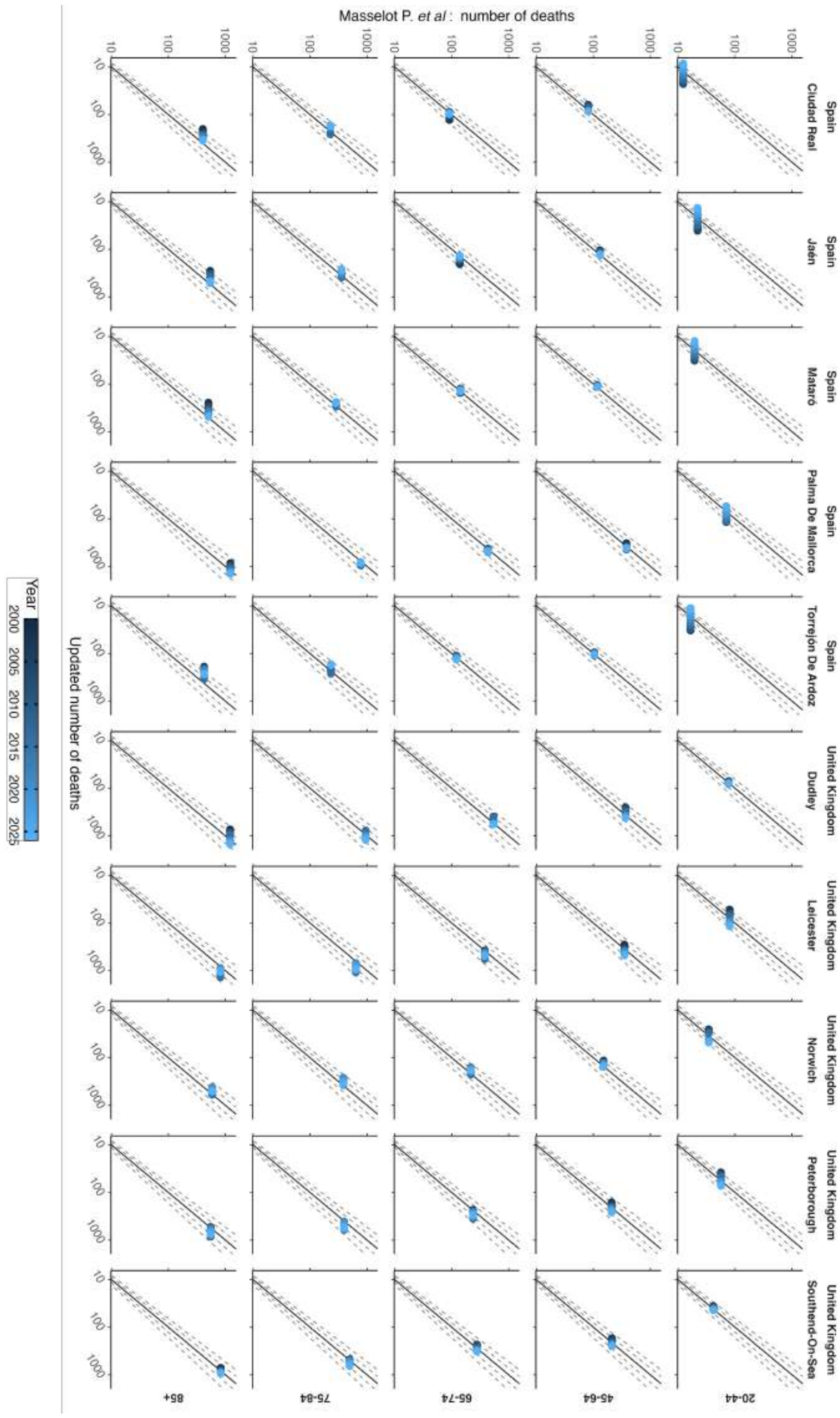
Masselot P. et al : number of deaths
Updated number of deaths
Year
2000
2005
2010
2015
2020
2025
Spain
Ciudad Real
Spain
Jaén
Spain
Mataró
Spain
Palma De Mallorca
Spain
Torrejón De Ardoz
United Kingdom
Dudley
United Kingdom
Leicester
United Kingdom
Norwich
United Kingdom
Peterborough
United Kingdom
Southend-On-Sea
20-44
45-64
65-74
75-84
85+

S2 Underestimation of European warming trends in climate models

Climate models are known to systematically underestimate the observed rate of warming across Europe, due to changes in atmospheric aerosols and circulation patterns that are not represented in the models [6–9]. It is therefore likely that the synthesised changes in temperature in Fig. 1 in the main text represent a conservative estimate of the true level of attributable warming across much of Europe. Fig. S4a shows a map of the historical change in temperatures in the 854 cities associated with 1.3°C of warming, obtained by averaging the change estimated from the two observational datasets. Panel b shows the average warming simulated by the 38 CMIP6 climate models, and panel c shows the difference between the two, highlighting where the underestimation is greatest.

While the spatial pattern of warming across the continent is fairly similar in models and observations, the climate models fail to simulate the full range of observed changes: in observations, the monthly changes range from 0.3 to 5.1°C, and in the climate models, from 0.9 to 2.6°C (Panel d, with the distribution of observed changes in blue and modelled changes in red). This is true in all calendar months but particularly in June, which has the widest spread of observed changes and the narrowest spread of modelled changes. The result of this is that for June temperatures, the synthesised changes (pink violins) are on average 0.6°C below the changes actually observed (95% central interval of differences: -1.2°C to +0.3°C); for July, 0.3°C below (-0.6°C to +0.2°C); and for August, 0.4°C below (-0.8°C to +0.2°C). To minimise this effect, we take the unweighted mean of the observed and modelled changes. However, this will still result in a lower estimate of attributable warming than the observed trend; the estimated temperature changes here should therefore be interpreted as a conservative estimate of the true level of human-caused warming across Europe. However, the inclusion of climate model data does produce better-constrained estimates of the level of attributable warming, with substantially narrower confidence intervals than those estimated from observations alone.

Fig. S4: Maps of estimated change in summer temperatures in (a) observational data products and (b) CMIP6 climate models; (c) differences between the two data sources. (d) Boxplot and violinplots of the estimated changes each month across all cities, in observations (blue) and models (red), with synthesised change in pink. The box represents the central 50% of the distribution, the whiskers indicate points within 1.5 times the interquartile range of the box, and circles indicate points more extreme than this.

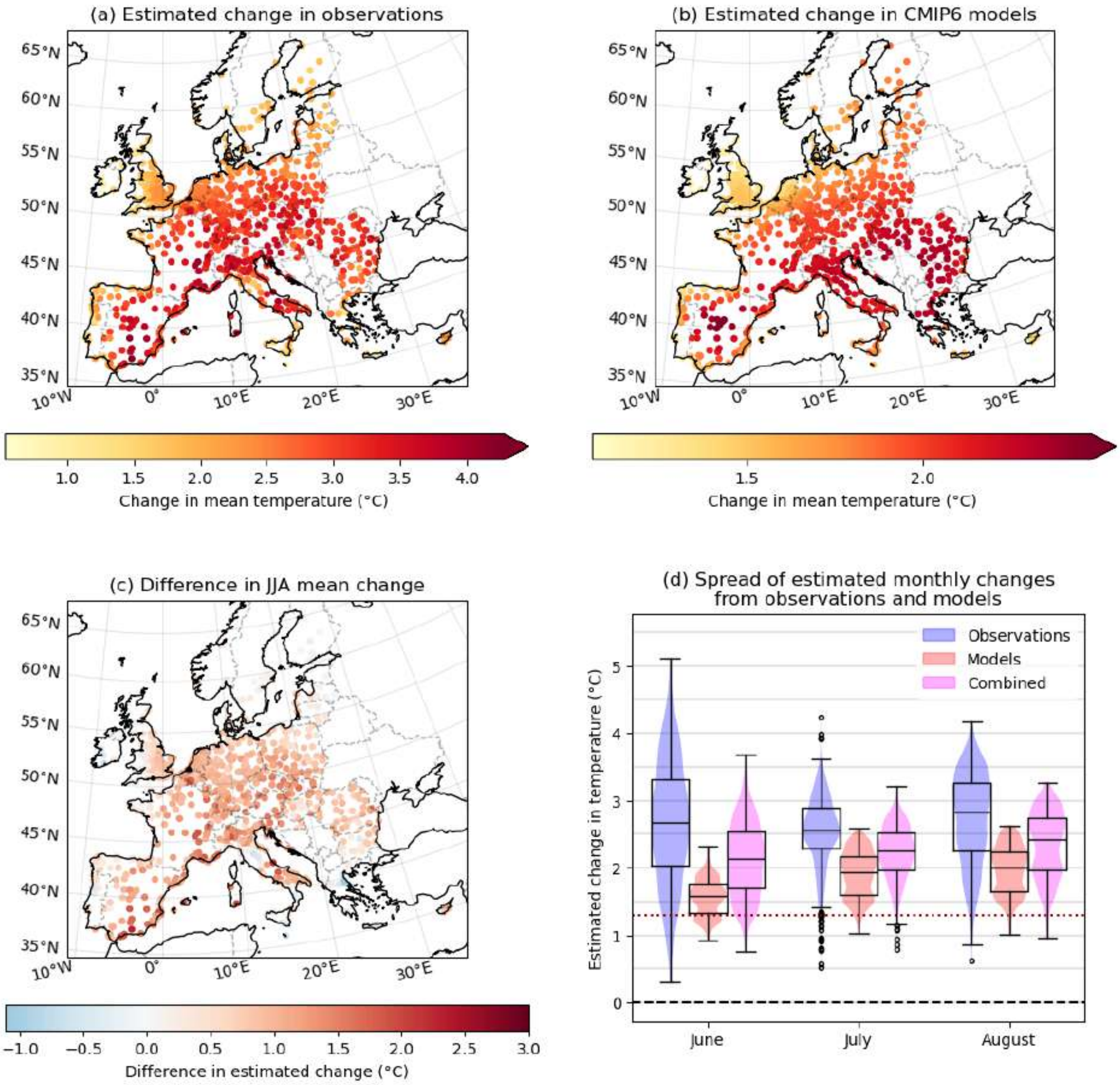

References

[1] WorldPop, Center for International Earth Science Information Network (CIESIN), Columbia University: Global High Resolution Population Denominators Project. Funded by The Bill and Melinda Gates Foundation (OPP1134076). WorldPop: School of Geography and Environmental Science, University of Southampton; Department of Geography and Geosciences, University of Louisville; Departement de Geographie, Universite de Namur (2018). https://doi.org/10.5258/SOTON/WP00647 . https://dx.doi.org/10.5258/SOTON/WP00647

[2] Bondarenko, M., Priyatikanto, R., Tejedor-Garavito, N., Zhang, W., McKeen, T., Cunningham, A., Woods, T., Hilton, J., Cihan, D., Nosatiuk, B., Brinkhoff, T., Tatem, A., Sorichetta, A.: The spatial distribution of population broken down by gender and age groupings in 2015-2030 at a resolution of 30 arc (approximately 1km at the equator), R2025A version v1. Global Demographic Data Project - funded by The Bill and Melinda Gates Foundation (INV-045237) (2025). https://doi.org/10.5258/SOTON/WP00846 . https://doi.org/10.5258/SOTON/WP00846

[3] Masselot, P., Mistry, M., Vanoli, J., Schneider, R., Iungman, T., Garcia-Leon, D., Ciscar, J.-C., Feyen, L., Orru, H., Urban, A., *et al.*: Excess mortality attributed to heat and cold: a health impact assessment study in 854 cities in Europe. The Lancet Planetary Health **7**(4), 271–281 (2023)

[4] Eurostat: Deaths by age group, sex and NUTS 3 region. Accessed: 2026-03-25 (2026). https://ec.europa.eu/eurostat/databrowser/view/demo_r_magec3/default/table?lang=en&category=reg.reg_dem.reg_demmor

[5] Eurostat: Deaths by week, sex, 5-year age group and NUTS 3 region. Accessed: 2026-03-16 (2026). https://ec.europa.eu/eurostat/databrowser/view/demo_r_mweek3/default/table?lang=en

[6] Van Oldenborgh, G.J., Wehner, M.F., Vautard, R., Otto, F.E.L., Seneviratne, S.I., Stott, P.A., Hegerl, G.C., Philip, S.Y., Kew, S.F.: Attributing and Projecting Heatwaves Is Hard: We Can Do Better. Earth's Future **10**(6), 2021–002271 (2022) https://doi.org/10.1029/2021EF002271 . _eprint: https://agupubs.onlinelibrary.wiley.com/doi/pdf/10.1029/2021EF002271. Accessed 2026-07-28

[7] Vautard, R., Cattiaux, J., Happé, T., Singh, J., Bonnet, R., Cassou, C., Coumou, D., D'andrea, F., Faranda, D., Fischer, E., *et al.*: Heat extremes in Western Europe increasing faster than simulated due to atmospheric circulation trends. Nature Communications **14**(1), 6803 (2023)

[8] Schumacher, D.L., Singh, J., Hauser, M., Fischer, E.M., Wild, M., Seneviratne, S.I.: Exacerbated summer European warming not captured by climate models neglecting long-term aerosol changes. Communications Earth & Environment **5**(1), 182 (2024) https://doi.org/10.1038/s43247-024-01332-8 . Accessed 2026-02-17

[9] Roldán-Gómez, P.J., Donat, M.G., Marcet-Carbonell, G., Smith, D.M.: Accelerated European Summer Warming Driven by Atmospheric Circulation Changes in Response to Aerosol Forcing. Geophysical Research Letters **53**(13), 2026–122424 (2026) https://doi.org/10.1029/2026GL122424 . _eprint:

https://agupubs.onlinelibrary.wiley.com/doi/pdf/10.1029/2026GL122424. Accessed 2026-07-27
