# Supplementary material for: Heat-related urban mortality attributable to human-induced climate change in Europe from 2000 to 2025
Source: Nat Health. Author manuscript; Available in PMC 2026 Oct 29. (PMC7619643; doi:10.1038/s44220-026-00674-w)
Supplement: Supplementary file 2 [file EMS221703-supplement-Peer_Review_File.pdf]

nature portfolio

Peer Review File

Multi-Country Projections of Temperature Related Suicide Mortality

Corresponding Author: Professor Yeonseung Chung

Parts of this Peer Review File have been redacted as indicated to maintain the confidentiality of unpublished data.

This file contains all reviewer reports in order by version, followed by all author rebuttals in order by version.

Version 0:

Reviewer comments:

Reviewer #1

(Remarks to the Author)
A. Summary of the key results
The manuscript, “Projections of temperature-related suicide mortality under climate change: A multi-country time-series study”, presents a global-scale projection of how rising temperatures may influence suicide mortality across 751 locations in 26 countries. Using a two-stage modeling approach, conditional Poisson regression with distributed lag non-linear models followed by multivariate mixed-effects meta-regression, the authors link historical temperature–suicide associations to projected future temperatures under shared socioeconomic pathway (SSP) 1-2.6, SSP2-4.5, and SSP5-8.5 scenarios. They report that suicide mortality attributable to temperature is expected to increase, especially in warmer regions, under all scenarios through the 2050s.

B. Originality and significance: if not novel, please include reference
The topic is highly relevant, methodologically rigorous, and of substantial societal and scientific interest. I believe the global scale and systematic approach make this an important and impactful contribution to climate change and mental health research.

C. Data & methodology: validity of approach, quality of data, quality of presentation
The methodology appears sound, rigorous, and appropriate for answering the research question. The authors use a two-stage approach: (1) conditional Poisson regression with distributed lag non-linear models for each location, and (2) multivariate meta-regression to pool estimates and produce projections using best linear unbiased predictions.
The dataset includes daily suicide and temperature data across 751 locations in 26 countries, covering a broad range of climates and socioeconomic contexts. Data sources are credible (e.g., Multi-Country Multi-City Collaborative Research Network). However, geographic representation is uneven, with underrepresentation in Africa, the Middle East, and parts of Southeast Asia. Importantly, the study appropriately acknowledges this limitation. The supplementary materials are detailed and transparent. Overall, I feel this complex methodology is clearly presented.

D. Appropriate use of statistics and treatment of uncertainties
The statistical methods are appropriate and sophisticated. The use of conditional Poisson regression with distributed lag non-linear models captures both non-linearity and lag effects of temperature on suicide. Meta-regression with best linear unbiased predictions is well-justified for pooling and predicting in countries with limited data. Empirical 95% confidence intervals (eCIs) were derived using Monte Carlo simulations (1,000 iterations), which is robust. Climate projection uncertainty is addressed by using five Global Circulation Models (GCM)s across three SSP scenarios. The meaning of the GCM acronym was only found in the SM. Please fix. Additionally, the authors could perhaps briefly explain how model, population, and behavioral uncertainties (e.g., lack of adaptation) might affect projections.

E. Conclusions: robustness, validity, reliability
The conclusions are broadly valid and robust, consistent with the analyses presented. The projections are associative, not causal, and are framed as such in the methodology, though the discussion occasionally slips into causal language. In the revision, please temper this phrasing slightly to ensure clarity and consistency. The findings that temperature-related suicide mortality may increase under all SSPs (especially in warmer regions) are reliable within the model’s assumptions.

F. Suggested improvements: experiments, data for possible revision
NA.

G. References: appropriate credit to previous work?
The manuscript cites a comprehensive and current body of literature

Clarity and context: lucidity of abstract/summary, appropriateness of abstract,
Clear and informative, but the following sections could be tempered ("e.g., "will experience large increases under all scenarios", "will experience smaller increases"); would something like "are projected to …" be more appropriate. Also, SSP is not written out in full in this section.
H. Introduction and conclusions

Introduction: Well-contextualized. Provides a clear rationale for the study, especially beyond single country projections.
Conclusions: Appropriate and aligned with the results. Overall writing is clear and well-organized.

Below I flag up some sections or areas I feel could be improved.

"Nonlinear relationships have been reported in East Asian countries such as the Republic of Korea and Taiwan, while linear patterns appeared in Western countries such as Canada, Spain, Switzerland, the UK, and USA, as well as in South Africa." Perhaps it would be useful for the lay readers if you briefly explain the actual patterns so they can understand these differences with clear examples.

"High-emission scenarios (could you give an example here) predict a substantial rise in heat-related mortality that will far outweigh the reductions in cold-related deaths. Specifically, warmer regions such as Southeast Asia are projected to experience sharp increases in mortality, and even temperate areas are expected to see increases despite reduced cold-related impacts (I was a bit confused what cold-related impacts were referring to, like an extended period of snow?).

"One study using the RCP8.5 high-emission" What is RCP?

"1.3% for SSP2-4.5, 0.6% for SSP1-2.6, and 2.4% for SSP5-8.5," Why not just go from low to high?

"22.7 °C in Costa Rica to 18.8 °C in Mexico", Again, why not go from low to high?

"As such, we did not account for population growth and aging, which could influence suicide trends." Perhaps including citations that have made these links.

"Australia and South Africa are each represented by a single country", do you mean Africa, the continent and Oceania the continent here?

I feel the Figures and Tables should be able to stand alone, so make sure you write out acronyms in full on their first appearance.

In the Supplemental Materials (SM), ANs need to be written out on the first appearance. I believe this is also the case for QAIC.

Fig.S1 seems to have an error; there are many blue dots around Pakistan.

Overall, excellent work and a delight to review.

(Remarks on code availability)
The authors provide thorough and transparent documentation of their approach, and while the shared data is simulated due to confidentiality constraints, the code and methodological details appear carefully considered and clearly presented. I did not formally rerun or test the accuracy of their models.

Reviewer #2

(Remarks to the Author)
Dear Editors and Authors, thank you for the opportunity to review this study that explored temperature related suicide mortality under climate change. Some comments for the authors to consider, including some points that require clarification or further detail. Hopefully, it can be helpful for further improvement.

Abstract

Line 108: It might be better to specify the observed data time frame here to clearly distinguish the baseline study period from the projection period from the 2010s to 2050s.

Line 112: Please clarify whether the mean temperature is daily, weekly, monthly or yearly.

Line 115: If only high-emission scenarios are reported, “respectively” can be deleted.

Lines 117-119: The statement “the difference in the total attributable fraction (AF) of suicide mortality ranged from 3.0% (95% eCI: 1.3, 6.0) in South Europe to 6.4% (3.9, 10.5) in Central America in the 2050s” is unclear. Does the 3.0% represent the difference from the 2010s under high-emission scenarios, or the total AF in the 2050s? Please clarify.

Line 121: Similarly, is the 1.2% figure a total AF by the 2050s or an increase from the 2010s? Please specify.

Introduction

Line 124: Please clarify whether this is the start of the Introduction or the Main section.

Lines 130, 141, 151, 160: Add line spacing between paragraphs for better readability.

Line 150: It would be better to give examples of these vulnerable populations you identified.

Line 163: by 2059 or by 2050s? 2059 is not mentioned in the Abstract and should be consistent throughout.

Methods

Line 355: The observational data vary across countries from 1971–2021, while the baseline association is defined for the 2010s (2010–2019). This poses challenges for countries lacking data during this baseline period (e.g., US data only available for 2001–2006, Mexico missing 2015–2019), similar for other countries. How were baseline associations in 2010s generated for the baseline period 2010s even without these data?

Line 356: Please define the 10 regions analysed. Do you need to indicate so, e.g. in Table 1 if applicable?

Line 364: Could I ask why use the modelled daily mean temperature data for the historical period, e.g. 1971-2024? While modelled daily mean temperature for future period can be understandable, but why historical temperature data you also used the modeled? The rationale for using modelled rather than observed data for historical periods should be clarified.

Line 376: Please add paragraph spacing. This applies to other sections as well.

Lines 378-380: After recalibration, was the modelled daily mean temperature data closely aligned with observed daily mean temperature data? Any evidence or metrics (e.g., correlation, RMSE) to demonstrate this alignment would be helpful.

Line 613: Table 2: Could you clarify how similar or close to the observed temperature during the baseline period 2010s, compared to the temperature that averaged across locations within each region under SSP1-2.6, SSP2-4.5, and SSP5-8.5 during the same baseline period?

Line 384: Do you also assume no adaptation here?

Line 405-406: Knots can be found in the supplementary material Table S1 for the justification. How was the lag decided? Please clarify. A concern is whether suicide outcomes occur so quickly within 3 days of temperature exposure?

Line 423: Also no changes in suicide rate?

Line 429: The sentence regarding “highest minimum temperatures... near the median of current temperatures” is unclear. Does this imply that the lowest minimum temperatures were not near the median? Please clarify.

Line 438: What is “decal ANs”? Please clarify.

Line 447: Were any sensitivity analyses conducted, for example, using different lags or temperature metrics?

Results

Lines 178, 188: Please add line spacing between paragraphs.

Line 228: Please clarify the top five regions referenced. Are they Central America, South Africa, South Europe, Southeast Asia, and Northern Europe? If so, why are Australia or South America excluded, especially if they experience relatively small temperature changes?

Line 230: The phrase “showing small or large temperature changes” is confusing, not very meaningful. Please clarify.

Line 231: Is it better to show results from some sensitivity analyses here to demonstrate the robustness of your study?

Discussion

Line 234: The study could be multiple countries study. I find it difficult to see this is a “global” study which missed most countries in these important regions such as Africa, the Middle East, Southeast Asia, and East Asia where the regions comprising over 40% of the global population.

Lines 276-289: These stressors can adversely affect mental health and contribute to rising suicide rate. It would be better to add contrasting statement and discussion to support this statement from both stressors and enablers, e.g. in Australia you showed the mild increase. However, my previously reviewed studies in Australia e.g. https://doi.org/10.1016/j.uclim.2021.101028 showed high mental health morbidity and cost due to climate change. Do such findings support or conflict with your study? Whether the high morbidity and cost due to climate change in Australia with higher average GDP to support your study on mitigating mortality despite increased morbidity? Please discuss.

Supplementary material

Line 21: Data in some countries did not cover the baseline period for the 2010s. Based on the data, how to generate the baseline associations? Please clarify.

Line 258: Font bold?

Line 318: Changing “Reference” to “References”?

(Remarks on code availability)

Reviewer #3

(Remarks to the Author)
Dear Editors and Authors

Thank you for the opportunity to review this interesting and highly relevant article. The authors model possible changes in suicide mortality on a population basis, based on three scenarios of socioeconomic, political and climate development in accordance with the so-called Shared Socioeconomic Pathways (SSP). The SSPs combine assumptions about possible political, economic and social developments, such as the further development of global economic power, international cooperation, public investment and population composition, and are part of the Sixth Assessment Report of the Intergovernmental Panel on Climate Change (IPCC). The epidemiological source data comes from the Multi Country Multi City Collaborative Research Network, which is coordinated by the London School of Hygiene & Tropical Medicine, and covers 751 locations in 26 predominantly highly industrialized countries. The modelling is based on the extrapolation of observed historical correlations between ambient temperature and suicide rates, for which data were aggregated for the following ten regions: North, Central and South America; North, Central and Southern Europe; East Asia; Southeast Asia; Australia; and South Africa. The time periods covered historically and the number of measurement points vary greatly between countries. The modelling horizon extends to the end of the 2050s. For simplicity's sake, the model assumes that neither the population structure nor suicide rates will change.

The article makes a valuable contribution to the scientific and political debate on the consequences of the climate crisis. I would recommend it for publication with some changes.

A Summary of the key results
In all three scenarios examined (SSP1-2.6, SSP2-4.5 and SSP5-8.5), suicide mortality, calculated here as the attributable fraction of total suicide mortality, increases depending on the region and the scenario used.

B Originality and significance: if not novel, please include reference
Following several publications providing forecasts for individual countries, this article is the first study to work with global data. However, developing countries, which are likely to be particularly affected by climate change, are hardly taken into account, obviously due to a lack of reliable data.

C Data & methodology: validity of approach, quality of data, quality of presentation
The model's approach leaves out key processes such as population development. Although this is unrealistic, it has been adequately explained and seems reasonable in order to limit the number of independent variables. To my knowledge, the SSPs also include assumptions about population development. To what extent these are sufficiently differentiated to be included in a model is beyond my knowledge.
The data used is appropriate. However, it is only representative to a limited extent, as there are few measuring points in some regions of the world and the measurement series are sometimes relatively short. The confidence intervals are therefore relatively wide in Central America and South Africa, for example.

D Appropriate use of statistics and treatment of uncertainties
Unfortunately, I am not in a position to assess the validity of the statistics used.

E Conclusions: robustness, validity, reliability

The conclusions are plausible, valid, and well-reasoned.

F Suggested improvements: experiments, data for possible revision
None

G References: appropriate credit to previous work?
The references seem adequate to me.

H Clarity and context: lucidity of abstract/summary, appropriateness of abstract, introduction and conclusions
For the most part, the text is fluent, catchy and easy to understand. One paragraph is difficult to understand in terms of content; I have listed this in detail below.

Comments in detail:

Manuscript
Line 105 - For clarity, I would use the term ambient temperature.
Line 157 - It would be more logical to keep the order of the scenarios according to radiative pressure or effective temperature, i.e. SSP1-2.6, SSP2-4.5, SSP5-8.5, especially since these are the same scenarios as in the submitted paper.
Line 160 – I would prefer: the "extent of the correlation" instead of "the strength of … relationship"
Line 172 – "…of Korea.", one could add: due to long time coverage and high population.
Line 193 / 260 – This finding, that the positive correlation between ambient temperature and suicides in East Asia and Australia practically disappears above a certain threshold, is very surprising at first glance. Can you think of any explanations for this?
Line 207 to 213 – Even after reading it several times, I still find this section hardly comprehensible. Elsewhere, it is argued that higher temperatures lead directly and indirectly to increased suicide mortality. At this point, if I understand correctly, we are talking about the contribution of low temperatures in the context of general global warming to suicide mortality. Does that mean you want to reflect the effects of temperature extremes in both directions? If that is the case, it should be introduced in a more comprehensible manner.
Line 238 – In any case, it is worth explaining why these three SSPs were selected.
Line 251 / 253 – "We observed regional variations in future … suicide mortality", "…the next five decades…" - Since we are in the year 2025 and the projection extends to 2059, this represents three and a half decades.
Line 267 to 280 - This section seems very important to me. Later on, possible ways in which increased temperatures can directly influence the suicide rate are discussed (lines 290 to 304). However, according to the literature, these effects are likely to be secondary in nature. Suicide is a very complex phenomenon with multiple causes. Decades of quantitative research have identified various risk factors that can be classified at the individual level into biological, psychological, clinical and social factors, such as the presence of mental illness, substance abuse, predisposed personality traits such as impulsivity, delinquency, poverty, loneliness, stressful life events, previous trauma, belonging to an ethnic minority or having a different sexual orientation or identity. In addition, environmental factors that affect the population as a whole are also significant. In particular, the influence of economic hardship has been robustly documented retrospectively. Since economically less developed countries in particular suffer from the consequences of climate change, these indirect effects are likely to be more significant in the end.
317 – The rising average age of the world's population will almost certainly have an impact on suicide behavior, as suicide, unlike suicide attempts, becomes much more likely with age.
336 – Another limitation, in my view, is that reporting on suicides in less developed countries is considered unreliable and many suicides are misclassified, whether for cultural or religious reasons or due to inadequate post-mortem examinations. Even in high-income countries, a certain degree of underreporting is assumed.

Supplemental information
Fig. S1 – A larger cloud of dots appears here in southern Pakistan, but this does not seem to be part of this investigation.

(Remarks on code availability)

Reviewer #4

(Remarks to the Author)
This manuscript estimates temperature-related suicide mortality for 751 locations across 26 countries and projects future impacts under multiple GHG emissions scenarios. Quantifying spatially heterogeneous risks is important for both adaptation and mitigation planning. However, there are substantive issues in the statistical specification and interpretation of results that affect the validity of the projections. I recommend major revision.

Major comments
Choice of reference temperature and exposure–response shape (DLNM)
The analyses set the suicide relationship at each location’s 50th percentile of temperature distribution as the reference the temperature. This is not generally appropriate. In time-series studies using DLNMs, the standard practice is to set the reference temperature at the Minimum Mortality/Morbidity Temperature (MMT)—the temperature at which the relative risk

(RR) is minimized (e.g., Gasparrini 2011; Peng et al. 2017; Zhou et al. 2023; Liang et al. 2024). The temperature higher or lower the MMT will cause relative risk greater than 1, which means the harmful risks cause by both hot and cold temperature. Setting the median as the reference temperature implies that temperatures below the 50th percentile often show RR < 1 across wide ranges, which is visible in Fig. 1 and is epidemiologically counter-intuitive. It doesn't make sense that all the exposure-response curve show relative risk lower than 1, i.e. no risks for the temperature lower the 50th percentile. I suggest the authors re-estimate the location-specific DLNMs following standard procedures: (i) identify the MMT for each location, (ii) re-center the cross-basis at the MMT, and (iii) compute RRs on the absolute temperature scale (°C), not percentiles. Please report the lag structure, spline bases/knots, and criteria used for model selection.

Implications for attributable fractions and numbers (AF/AN)

With the current centering, temperatures below the 50th percentile yield RR < 1, producing negative AF (AF=(RR-1)/RR×100). The net effect then becomes a cancellation between positive AF (hot side) and negative AF (cold side), obscuring true burdens. This further highlights the problems of defining the exposure–response function by using the 50th percentile temperature as the reference point, as noted in my first comment. Therefore, the projection of impact of climate change on suicide should be redone based on the new exposure response function established as I suggested above. Therefore, I would recommend the authors to compute and present hot- and cold-attributable burdens separately as well as the net total, after recalculated the exposure-response function using MMT. This is standard practice and avoids misleading cancellations.

Report attributable numbers (AN), not only AF

The Methods state that ANs were calculated, but Figs. 2–4 present only AFs. For policy relevance, decision-makers need case counts. The authors should add figures/tables showing AN (with 95% uncertainty intervals), and specify baseline populations and suicide rates used, projection time slices, and how uncertainties from exposure–response functions and climate projections were propagated.

Scenario coverage in Figs. 3–4

Figs. 3–4 show results only for SSP5-8.5, whereas Fig. 2 includes SSP1-2.6, SSP2-4.5, and SSP5-8.5. Please include all three scenarios in Figs. 3–4 (or provide parallel panels/appendix figures). This will materially improve the paper's mitigation insights.

Characterizing extremes

Suicide risks are plausibly more sensitive to extreme heat/cold than to decadal mean temperatures. In Table 2 (and/or a new table), I suggest the authors to add regional statistics for relevant extreme indices (e.g., hot-day/hot-night frequencies, heatwave metrics, TXx/TNn) consistent with the exposure metric used in the DLNM.

Discontinuity in Northern Europe in Fig. S3

There appears to be a discontinuity around 2010 between historical and projected temperature changes for Northern Europe. Please check the stitching of historical and future series (bias correction, baseline alignment, ensemble concatenation) and correct the discontinuity.

Model transparency and confounding control

Ensure the main text or supplement clearly states: over-dispersion handling; seasonality/long-term trend control; day-of-week/holiday adjustments; lag length justification; meta-analytic pooling (random-effects), any effect-modification analyses (e.g., by income, baseline suicide rate, health system indicators), and calculation details of AF and AN. This information is essential for reproducibility and interpretation.

The manuscript would benefit from professional English editing to improve clarity and readability.

Reference

Gasparrini, A. Distributed lag linear and non-linear models in R: the package dlnm. J. Stat. Softw. 43, 1–20 (2011)
Liang, C., Yuan, J., Tang, X., Kan, H., Cai, W., Chen, J. The influence of humid heat on morbidity of megacity Shanghai in China. Environment International, 183, 108424 (2024).
Peng, Z., Wang, Q., Kan, H., Chen, R. & Wang, W. Effects of ambient temperature on daily hospital admissions for mental disorders in Shanghai, China: a time-series analysis. Sci. Total Environ. 590, 281–286 (2017).
Zhou, Y., Y. Gao, P. Yin, C. He, W. Liu, H. Kan, M. Zhou, and R. Chen Assessing the Burden of Suicide Death Associated With Nonoptimum Temperature in a Changing Climate. JAMA Psychiatry, 80, 488–497 (2023).

(Remarks on code availability)

Version 1:

Reviewer comments:

Reviewer #1

(Remarks to the Author)
I have carefully read the authors' responses to all reviewer comments, particularly those addressing my own points. I am satisfied that my concerns have been fully addressed, and I have no further suggestions for revision. The manuscript has been strengthened through the revision process, excellent work.

(Remarks on code availability)

Reviewer #2

(Remarks to the Author)
Dear Editors and Authors, thank you for the opportunity to review the revised manuscript. The paper has been much improved. I have one minor comment for the authors to consider at this stage before I recommend acceptance.

Methods:
Lines 440–444 indicated that temperature projections were obtained under three SSP scenarios, representing combined projections of future socioeconomic development and greenhouse gas emissions, ranging from mitigation (SSP1-2.6) to intermediate stabilization (SSP2-4.5) and high-emission (SSP5-8.5) scenarios. However, lines 467–468 stated that the projections assume no changes in population structure, and lines 475–476 further indicated no adaptation and no change in population structure. Given that SSP scenarios inherently consider and incorporate assumptions about future population dynamics and adaptation, there appears to be some inconsistency between the use of SSP scenarios and the assumption of no population change and no adaptation. If the authors have used SSPs solely as sources of temperature projections, this should be more clearly stated and justified. Alternatively, the authors may consider using RCP-based temperature projections.

(Remarks on code availability)

Reviewer #3

(Remarks to the Author)
Dear Yeonseung Chung,
dear Authors,

you have incorporated my comments into your manuscript very carefully. I recommend your paper for publication without further revision.

best regards

(Remarks on code availability)

Reviewer #4

(Remarks to the Author)
The revised manuscript has improved substantially in clarity, structure, and interpretation. In particular, the revisions to methodology clarification, sensitivity analyzes, and complete scenarios coverage have strengthened the overall quality and rigor of the study.
I believe the manuscript is now close to being suitable for publication. I recommend minor revision, primarily to further strengthen the scholarly positioning of the work.
1. In the Introduction, citing several recently published studies would help better situate the findings within the current body of literature and clarify the manuscript's contribution relative to prior work. This is particularly important given the broad readership of the journal. Below are examples of relevant recent publications that could be considered.
a. Liang, C., Yuan, J.*, Zhang, R. et al. Projecting the morbidity burden of mental and behavioral disorders associated with increasing humid heat in Shanghai. Nat. Mental Health (2025). https://doi.org/10.1038/s44220-025-00519-y
b. Min, H. et al. Ambient temperature, suicide, and urbanicity: A nationwide time-stratified case-crossover study in South Korea. PLOS ONE 20, e0337945 (2025).
c. Baecker L, et al. (2025). Impacts of extreme heat on mental health: Systematic review and qualitative investigation of the underpinning mechanisms. Journal of Climate Change and Health, 22: 100446. https://doi.org/10.1016/j.joclim.2025.100446

2. Some additional limitations should be discussed. First, the analysis focuses on daily mean temperature and does not explicitly account for the potentially distinct effects of extreme heat or cold events. Second, suicide count data rather than rate-based data were used, as information on population size and temporal changes in suicide rates was not available.

(Remarks on code availability)

Version 2:

Reviewer comments:

Reviewer #2

(Remarks to the Author)

Dear Editors and Authors, thank you for the opportunity to review the revised manuscript. My concerns have been addressed. I do not have further comments.

(Remarks on code availability)

Reviewer #4

(Remarks to the Author)
The authors have adequately addressed all of my concerns. I recommend acceptance in Nature Mental Health without any further revisions.

(Remarks on code availability)

Open Access This Peer Review File is licensed under a Creative Commons Attribution 4.0 International License, which permits use, sharing, adaptation, distribution and reproduction in any medium or format, as long as you give appropriate credit to the original author(s) and the source, provide a link to the Creative Commons license, and indicate if changes were made.
In cases where reviewers are anonymous, credit should be given to 'Anonymous Referee' and the source.
The images or other third party material in this Peer Review File are included in the article's Creative Commons license, unless indicated otherwise in a credit line to the material. If material is not included in the article's Creative Commons license and your intended use is not permitted by statutory regulation or exceeds the permitted use, you will need to obtain permission directly from the copyright holder.
To view a copy of this license, visit https://creativecommons.org/licenses/by/4.0/

Dear Editor,

We would like to express our sincere gratitude to you and all four reviewers for your thoughtful and constructive comments on our manuscript entitled "Projections of temperature-related suicide mortality under climate change: A multi-country time-series study"

We believe that the manuscript has been substantially strengthened through the revision process in response to their insightful suggestions.

The following pages provide a detailed, point-by-point response to all reviewers' comments, along with explanations of the corresponding revisions made in the manuscript. Original reviewer comments are shown in bold, and our responses follow each comment accordingly.

Comments from Reviewer 1

#1.1. Appropriate use of statistics and treatment of uncertainties

The meaning of the GCM acronym was only found in the SM. Please fix. Additionally, the authors could perhaps briefly explain how model, population, and behavioral uncertainties (e.g., lack of adaptation) might affect projections.

[Response]

The full term "general circulation model (GCM)" has been in the main text. (Line 446, page 17, main text)

> **(Line 446, page 17, in main text)**
> five general circulation models (GCMs)

In addition, we fully agree that the projections in our study may be affected by multiple sources of uncertainty. In response to your suggestion, we have added and/or strengthened the discussion in the revised manuscript regarding (1) uncertainty arising from exposure-response relationship modeling, (2) climate model uncertainty, (3) uncertainty related to future demographic changes, and (4) behavioral/adaptation-related uncertainty.

In brief, we accounted for modeling-related uncertainty in our analysis, (1) & (2), as described in detail in the *Methods* section. (Lines 558–567, page 22, in main text) However, uncertainties arising from future demographic changes (3) and from behavioral/adaptation-related factors (4) were not incorporated in this study, and we therefore described these as limitations in the *Discussion* section. (Lines 374-385, page 15, in main text)

> **(Lines 558–567, page 22, in main text)**
> We quantified the uncertainty in future temperature-related suicide mortality using Monte Carlo simulations at the meta-regression-model level as previously described[64] (see Section 2.2.2 in the Supplementary Material). These simulations provided eCIs that accounted for the correlation among location-specific predictions and uncertainty associated with the spatialization of random effects in the predictive meta-regression model. In addition, uncertainty from climate projections was incorporated by combining temperature estimates from five GCMs under three SSP scenarios, ensuring that the final impact estimates reflect both epidemiological and sources of uncertainty. This approach captures the uncertainty in multiple stages of the analysis—including exposure-response estimation and climate projections—and reduces the risk of underestimation, particularly in analyses involving small-area data and low heterogeneity[64].
>
> **(Lines 374–385, page 15, in main text)**
> Several limitations should be acknowledged in this study. First of all, in order to isolate the changes solely due to climate change, our projections were based on simplified assumptions, including constant suicide rates, no human adaptation, and a static population. As such, we did not account for population aging, despite evidence that suicide rates tend to increase among individuals aged 60 years and older[52]. We used a fixed baseline suicide rate and did not account for potential temporal changes in baseline suicide rates. Furthermore, we did not consider the potential for human adaptation to rising temperatures,

which may mitigate suicide risk in warmer climates. Also, to the extent that larger estimates in warmer countries are modified by socio-economic status, we did not capture any changes that might occur due to faster economic growth in some of these countries. Consequently, projections assuming no adaptation may overestimate the future burden. Additionally, we did not consider temporal demographic shifts which could alter the relationship between temperature and suicide risk. For instance, susceptibility to temperature-related suicide may change according to demographic change such as age and racial groups.

#1.3. Conclusions: robustness, validity, reliability

The projections are associative, not causal, and are framed as such in the methodology, though the discussion occasionally slips into causal language. In the revision, please temper this phrasing slightly to ensure clarity and consistency.

[Response]

We have carefully revised the *Discussion* section to ensure that all descriptions emphasize associative rather than causal interpretations. Accordingly, causal verbs such as "reason" and "due to" were replaced with neutral terms such as "explanation", "associated with" or "linked to," and additional words like "mediated by" or "indirectly influenced by" were included where appropriate. (Lines 322–334, page 13, in main text) We also replaced "suicide attributable to temperatures" with "suicide associated with temperatures.

(Lines 322–334, page 13, in main text)

Another potential explanation for regional variations in future temperature-related suicide mortality may be differences in socioeconomic conditions. Except for South Europe, the warmer regions that showed sharp increases in suicide mortality associated with temperatures over time often overlapped with countries of relatively low socioeconomic status (based on average GDP, Table 1). In contrast, regions where suicide risk increased more gradually tended to comprise countries with higher average GDP. These findings suggest that the impact of climate change on suicide mortality may mediated indirectly by regional disparities in economic stability and social infrastructure. Climate change can exacerbate existing inequalities, as vulnerable populations and low-income countries face greater economic instability[35,36], escalating conflicts[37-39], and declining agricultural productivity/food security[40]. Moreover, in regions with limited access to mental health care and underdeveloped infrastructure, the lack of timely intervention and support may heighten the burden on vulnerable populations[36]. Therefore, the link between rising temperatures and suicide mortality may reflects, at least in part, the indirect consequences of socioeconomic hardship amplified by climate change.

#1.4. Clarity and context: lucidity of abstract/summary, appropriateness of abstract, Clear and informative, but the following sections could be tempered ("e.g., "will experience large increases under all scenarios", "will experience smaller increases"); would something like "are projected to …" be more appropriate. Also, SSP is not written out in full in this section.

[Response]

We revised the *abstract* section to replace deterministic language by "are projected to". (Lines 121 and 125, page 5, in main text) And we provided the full term "shared socioeconomic pathway (SSP)" at first appearance. (Lines 115-116, page 5, in main text)

(Lines 120–121, page 5, in main text)
Warmer regions, including Central and South America, Southern Europe, Southeast Asia, and South Africa, are projected to have larger increases under all scenarios.

(Lines 124–125, page 5, in main text)
Temperate and colder regions, such as North America, North and Central Europe, East Asia, and Australia, are projected to show smaller increases**,**

(Lines 115–116, page 5, in main text)
These associations were then linked to future temperatures under climate change scenarios, specifically the shared socioeconomic pathways SSP1-2.6, SSP2-4.5, and SSP5-8.5.

#1.5. "Nonlinear relationships have been reported in East Asian countries such as the Republic of Korea and Taiwan, while linear patterns appeared in Western countries such as Canada, Spain, Switzerland, the UK, and USA, as well as in South Africa." Perhaps it would be useful for the lay readers if you briefly explain the actual patterns so they can understand these differences with clear examples.

[Response]

We have revised the text to include regional examples of inverse J-shaped and linear patterns (Lines 144–150, page 6, in main text). These additions clarify that East Asian countries (e.g., Japan, the Republic of Korea, and Taiwan) exhibit inverse J-shaped relationships, whereas Western countries (e.g., Canada, Spain, Switzerland, the United Kingdom, and the United States) display nearly linear associations.

(Lines 144–150, page 6, in main text)
Nonlinear inverse J-shaped relationships have been reported in East Asian countries such as Japan, the Republic of Korea and Taiwan, where suicide risk tends to decrease at extremely high temperatures after reaching a peak. In contrast, nearly linear patterns appeared in Western countries such as Canada, Spain, Switzerland, the UK, and USA, where suicide risk continued to increase with higher temperatures. Meanwhile, in the Philippines, Brazil, Vietnam, and South Africa, the nonlinear curves were unclear or showed large uncertainty.[20]

#1.6. "High-emission scenarios (could you give an example here) predict a substantial rise in heat-related mortality that will far outweigh the reductions in cold-related deaths. Specifically, warmer regions such as Southeast Asia are projected to experience sharp increases in mortality, and even temperate areas are expected to see increases despite reduced cold-related impacts (I was a bit confused what cold-related impacts were referring to, like an extended period of snow?).

[Response]

We added a specific example of a high-emission scenario, Representative Concentration Pathway 8.5 (RCP8.5), which assumes continued high greenhouse gas emissions. (Lines 156–158, page 7, in main text)

In this study, "cold-related impacts" do not necessarily refer to prolonged snow events or general winter conditions, but rather to cold-related excess mortality (i.e., mortality attributable to temperatures below the minimum mortality temperature, MMT). Accordingly, we clarified and added the definitions of heat-related excess mortality (i.e., mortality attributable to temperatures above the MMT) and cold-related excess mortality (i.e., mortality attributable to temperatures below the MMT). (Lines 159–162, page 7, in main text)

In addition, we provided a more detailed explanation of regional differences, warmer regions (e.g., Central and South America, Southern Europe, and particularly Southeast Asia) are projected to experience sharp increases in heat-related excess mortality (Lines 158–162, page 7, in main text), whereas temperate regions (e.g., Northern Europe, East Asia, and Australia) are expected to show smaller net increases due mainly to reductions in cold-related mortality (Lines 163–165, page 7, in main text)).

**(Lines 156–167, page 7, in main text)**
Under high-emission scenarios such as representative concentration pathway 8.5 (RCP8.5)—which assumes continued high greenhouse gas emissions—warmer regions, including Central and South America, South Europe, and particularly South-East Asia, exhibit sharp increases in heat-related excess mortality (i.e., mortality attributable to temperatures above the minimum mortality temperature, MMT), which outweigh reductions in cold-related excess mortality (i.e., mortality attributable to temperatures below the MMT).[26,27] Consequently, the net change in temperature-related excess mortality is projected to rise substantially in these regions. In contrast, temperate regions such as North Europe, East Asia, and Australia, where warming is less intense, show smaller net increases in temperature-related excess mortality, primarily due to pronounced declines in cold-related mortality.[26,27]

#1.7. "One study using the RCP8.5 high-emission" What is RCP?
[Response]
We added an explanation of "RCP" in the preceding paragraph (Lines 156–158, page 7, in main text), specifying that it stands for Representative Concentration Pathway, which describes greenhouse gas concentration trajectories.

**(Lines 156–158, page 7, in main text)**
Under high-emission scenarios such as representative concentration pathway 8.5 (RCP8.5), which assumes continued high greenhouse gas emissions,

#1.8. "1.3% for SSP2-4.5, 0.6% for SSP1-2.6, and 2.4% for SSP5-8.5," Why not just go from low to high?
[Response]
We revised as suggested "0.6% for SSP1-2.6, 1.3% for SSP2-4.5, and 2.4% for SSP5-8.5" (Line 175, page 7, in main text).

**(Lines 174–176, page 7, in main text)**

In the 2090s, the projected increases compared to the 2010s were estimated under different shared socioeconomic pathway (SSP) scenarios, 0.6% for SSP1-2.6, 1.3% for SSP2-4.5, and 2.4% for SSP5-8.5, with greater increases under more extreme scenarios

#1.9. "22.7 °C in Costa Rica to 18.8 °C in Mexico", Again, why not go from low to high?
[Response]
We revised as suggested "18.8°C in Mexico to 22.7°C in Costa Rica". (Lines 199-200, page 8, in main text)

(Lines 199–200, page 8, in main text)
Central America (18.8 °C in Mexico to 22.7 °C in Costa Rica).

#1.10. "As such, we did not account for population growth and aging, which could influence suicide trends." Perhaps including citations that have made these links.
[Response]
We cited Shah et al. (2016) to support the finding that suicide rates increase among older adults. Specifically, Shah et al. (2016) reported a consistent rise in suicide rates with advancing age among individuals aged 60 years and older. (Lines 376-378, page 15, in main text)

Also, we removed "population growth" because its relationship with suicide varies considerably across contexts, with studies showing various findings—some reporting higher elderly suicide rates in countries with low or rapidly increasing population growth (Shah A, 2009), while others suggesting an inverse association between population increase and suicide (Zhang J., 1998). Therefore, we considered it ambiguous to generalize this effect.

(Lines 376–378, page 15, in main text)
As such, we did not account for population aging, despite evidence that suicide rates tend to increase among individuals aged 60 years and older.

Reference
Shah A. et. al. (2016). Suicide rates in five-year age-bands after the age of 60 years: the international landscape. Aging & mental health, 20(2), 131–138. https://doi.org/10.1080/13607863.2015.1055552

Zhang J. (1998). Suicide in the world: toward a population increase theory of suicide. Death studies, 22(6), 525–539. https://doi.org/10.1080/074811898201380

Shah A. (2009). The relationship between population growth and elderly suicide rates: a cross-national study. International psychogeriatrics, 21(2), 379–383. https://doi.org/10.1017/S1041610208007953

#1.11. "Australia and South Africa are each represented by a single country", do you mean Africa, the continent and Oceania the continent here?
[Response]

We revised the text to clarify that, unlike other regions composed of multiple countries (e.g., Northern Europe, East Asia), Australia and South Africa were analyzed as individual countries. (Lines 400–403, page 16, in main text)

(Lines 400–403, page 16, in main text)
Furthermore, Australia and South Africa represent a single country rather than the entire continent, although they are compared individually with other regions. It may not fully reflect regional and climate variations, potentially limiting the broader applicability of the findings.

#1.12. I feel the Figures and Tables should be able to stand alone, so make sure you write out acronyms in full on their first appearance.

[Response]

We revised the title and caption of all figures and tables in main manuscript and supplementary materials to write out acronyms in full.

#1.13. In the Supplemental Materials (SM), ANs need to be written out on the first appearance. I believe this is also the case for QAIC.

[Response]

We added the full term "attributable number (AN)" at its first appearance (Line 243, page 9, Supplementary Materials) and the full term "quasi Akaike's Information Criterion (QAIC)" at its first appearance. (Lines 174-175, page 7, Supplementary Materials)

(Line 243, page 9, Supplementary Materials)
Using these reference temperatures, the attributable number (AN) and fraction (AF) of suicide mortality

(Lines 174–175, page 7, Supplementary Materials)
The number of knots and its locations were determined based on quasi Akaike's Information Criterion (QAIC) values in Table S4

#1.14. Fig.S1 seems to have an error; there are many blue dots around Pakistan.

[Response]

Fig. S1 has been corrected and replaced in the Supplementary Materials. (Lines 331-335, page 20, Supplementary Materials)

Fig. S1 | Map of the average temperatures at the 751 locations included in the analysis.

Redacted

Comments from Reviewer 2

#2.1. Line 108: It might be better to specify the observed data time frame here to clearly distinguish the baseline study period from the projection period from the 2010s to 2050s.

[Response]

We revised the text to clearly distinguish the observed data period (1971–2021) from the projection period (2010–2059). Specifically, we clarified that the location-specific temperature–suicide associations were estimated using observed data from 1971 to 2021, whereas the projections were conducted for decades from the baseline 2010s (2010–2019) to the 2050s (2050–2059). (Lines 110–118, page 5, in main text)

> **(Lines 110–118, page 5, in main text)**
> We collected observed time-series data on daily mean temperature and daily suicide counts for 751 locations in 26 countries from 1971-2021 from the Multi-Country Multi-City Collaborative Research Network and modeled temperature data from 1971-2059 from the Coupled Model Intercomparison Project Phase 6 (CMIP6). Using a two-stage analysis, location-specific temperature–suicide associations were estimated based on observed data. These associations were then linked to future temperatures under climate change scenarios, specifically the shared socioeconomic pathways SSP1-2.6, SSP2-4.5, and SSP5-8.5. Then, temperature-related suicide mortality was projected by decade from the baseline 2010s (2010–2019) to the 2050s (2050–2059), assuming no adaptation and no changes in population structure or suicide rate.

#2.2. Line 112: Please clarify whether the mean temperature is daily, weekly, monthly or yearly.

[Response]

We added the term "daily" to clarify that the mean temperature refers to the daily mean temperature. (Line 110-111, page 5, in main text)

> **(Line 110-111, page 5, in main text)**
> We collected observed time-series data on daily mean temperature and daily suicide counts

#2.3. Line 115: If only high-emission scenarios are reported, "respectively" can be deleted.

[Response]

We deleted "respectively" as suggested. (Line 119-120, page 5, in main text)

> **(Line 119–120, page 5, in main text)**
> Total attributable fractions (AF) of suicide mortality increased over decades in all scenarios, especially under the high-emission scenarios (SSP5-8.5).

#2.4. Lines 117-119: The statement "the difference in the total attributable fraction (AF) of suicide mortality ranged from 3.0% (95% eCI: 1.3, 6.0) in South Europe to 6.4% (3.9, 10.5) in Central America in the 2050s" is unclear. Does the 3.0% represent the difference from the 2010s under high-emission scenarios, or the total AF in the 2050s? Please clarify.

[Response]

We revised the sentences to clarify that the percentages represent the differences in total attributable fraction (AF) of suicide mortality in the 2050s relative to the 2010s baseline. (Lines 122-123, page 5, in main text)

> **(Lines 122–123, page 5, in main text)**
> Under the high-emission scenario, the difference in total attributable fraction (AF) of suicide mortality in the 2050s relative to the baseline 2010s

#2.5. Line 121: Similarly, is the 1.2% figure a total AF by the 2050s or an increase from the 2010s? Please specify.
[Response]
We revised the sentences to clarify that the percentages represent the differences in total attributable fraction (AF) of suicide mortality in the 2050s relative to the 2010s baseline. (Lines 124-127, page 5, in main text)

> **(Lines 124–127, page 5, in main text)**
> Temperate and colder regions, such as North America, North and Central Europe, East Asia, and Australia, are projected to show smaller increases, with differences in total AF in the 2050s relative to the baseline 2010s ranging from 1.5% (0.4, 3.3) in North Europe to 2.0% (1.2, 3.5) in East Asia.

#2.6. Line 124: Please clarify whether this is the start of the Introduction or the Main section.
[Response]
We added the section title "**Introduction**" in the beginning of the *Introduction* section. (Line 129, page 6, in main text)

#2.7. Lines 130, 141, 151, 160: Add line spacing between paragraphs for better readability.
[Response]
We added line spacing between paragraphs throughout the *Introduction* section.

#2.8. Line 150: It would be better to give examples of these vulnerable populations you identified.
[Response]
We replaced the wording "vulnerable populations" with "populations living in warmer and, in some cases, poorer regions". (Lines 165–167, page 7, in main text)

> **(Lines 165–167, page 7, in main text)**
> These findings underscore the importance of policies to mitigate global warming and the disproportionate health risks faced by populations living in warmer and, in some cases, poorer regions.

#2.9. Line 163: by 2059 or by 2050s? 2059 is not mentioned in the Abstract and should be consistent throughout.
[Response]
This sentence does not describe the results of our study mentioned in the Abstract; rather, it describes the findings from a previous study. But to make it clearer, we changed the expression to "up to the year 2050" (Line 170-173, page 7, main text).

(Lines 170–173, page 7, in main text)
One study using the high-emission scenario RCP8.5 estimated that a 1-°C monthly average temperature increase would cause a 0.7% rise in suicide rates in US counties and 2.1% rise in Mexican municipalities, potentially resulting in 9,000-40,000 additional suicides up to the year 2050.[30]

#2.10. Line 355: The observational data vary across countries from 1971–2021, while the baseline association is defined for the 2010s (2010–2019). This poses challenges for countries lacking data during this baseline period (e.g., US data only available for 2001–2006, Mexico missing 2015–2019), similar for other countries. How were baseline associations in 2010s generated for the baseline period 2010s even without these data?

[Response]

We acknowledge that our previous explanation was unclear, and we have revised the manuscript to clarify this point. The reviewer may have interpreted the observation period for the empirical data and the baseline period used for projections as the same; however, these represent two distinct periods. Specifically, the empirical observation period spans 1971–2021 (with country-specific variations), and these data were used to estimate the temperature–mortality association. We then linked the estimated associations with modeled temperature data for 2011–2059 to project the temperature-attributable suicide burden by decade. To examine decadal trends, we quantified differences in projected suicide burden for the 2020s, 2030s, 2040s, and 2050s relative to the baseline 2010s.

(Lines 439–441, page 17, in main text)
The projection periods are defined by decade, starting from the 2010s (2010–2019) and continuing through the 2050s (2050–2059).

(Lines 472–476, page 18-19, in main text)
First, location-specific temperature-suicide associations were estimated based on the observed data using a two-stage approach. Second, the estimated associations were extrapolated using the modeled temperature up to the year 2059, and future increases in suicide mortality associated with temperatures were calculated under three important assumptions—no adaptation, no change in population structure, and no change in suicide rate.

(Lines 531–538, page 21, in main text)
For each combination of SSPs and GCMs, based on these RRs, daily attributable numbers (ANs) were calculated for each day from 2010 to 2059 at each location for all combinations of GCMs and SSP scenarios. The location-specific ANs were then aggregated by region, SSP, and decade, and averaged across GCMs. The region-specific decadal ANs were divided by the total number of suicide deaths in each region to obtain decadal attributable fraction (AF) of suicide mortality associated with temperatures.[26,31,32,62] Finally, the difference in AF for each decade (2020s–2050s) relative to the 2010s (as presented in Fig. 2) was calculated to assess changes in future suicide burden associated with climate warming.

#2.11. Line 356: Please define the 10 regions analysed. Do you need to indicate so, e.g. in Table 1 if applicable?
[Response]
We added the names of the ten regions in the revised text. Also, Table 1 lists the countries included in each region. (Lines 428-432, page 17, in main text)

(Lines 428–432, page 17, in main text)
The 26 countries were categorized into 10 regions based on geographical and climatic characteristics: North America, Central America, South America, North Europe, Central Europe, South Europe, East Asia, Southeast Asia, Australia, and South Africa, with the latter two each representing a single-country region. Table 1 shows the countries included in each region along with their respective observed data periods.

#2.12. Line 364: Could I ask why use the modelled daily mean temperature data for the historical period, e.g. 1971-2024? While modelled daily mean temperature for future period can be understandable, but why historical temperature data you also used the modeled? The rationale for using modelled rather than observed data for historical periods should be clarified.
[Response]
We appreciate the reviewer's helpful clarifying question. In our study, the modeled temperature data from the historical period (1971–2021; with country-specific variations) and from the future projection period (2010–2059) were used for two distinct purposes.

First, the modeled data from the historical period were used to derive the calibration formula by comparing modeled temperatures with observed temperatures. In other words, calibration parameters were estimated based on the differences between modeled and observed data during the historical period. We followed the calibration approach described in a previous tutorial for projection studies (Vicedo-Cabrera et al., 2019), and the relevant details are provided in the main text (Lines 456–462, page 18). Second, the modeled data from the future projection period were used, after applying this calibration, to project temperature-attributable suicide burdens by decade from the baseline 2010s to the 2050s.

(Lines 456–462, page 18, in main text)
To adjust for differences between the modeled and observed daily mean temperatures, the modeled temperature data were recalibrated as previously described[62] using monthly averages and daily variability in the observed mean temperature data[63]. Regions showing large median differences (>5 °C) between observed and modeled temperatures after adjustment underwent a secondary bias correction by aligning their future mean temperatures with the national mean. Country-specific RMSE values, presented in Table S3, indicate close alignment between the calibrated modeled and observed temperatures.

Reference
62. Vicedo-Cabrera, A. M., Sera, F. & Gasparrini, A. Hands-on tutorial on a modeling framework for projections of climate change impacts on health. Epidemiology 30(3), 321–329 (2019).

https://doi.org/10.1097/EDE.0000000000000982

#2.13. Line 376: Please add paragraph spacing. This applies to other sections as well.
[Response]
We added line spacing between paragraphs throughout the *Methods* section.

#2.14. Lines 378-380: After recalibration, was the modelled daily mean temperature data closely aligned with observed daily mean temperature data? Any evidence or metrics (e.g., correlation, RMSE) to demonstrate this alignment would be helpful.
[Response]
We reported RMSE values before and after bias correction for each climate scenario in Table S3 of the Supplementary Material (Lines 306–310, page 15, Supplementary Materials). Overall, the RMSE values generally decreased after recalibration.

Table S3 | Country-specific root mean square errors (RMSEs) between modeled and observed temperatures before and after calibration under three climate scenarios

RMSE / Country	SSP1-2.6		SSP2-4.5		SSP5-8.5	
	**Before**	**After**	**Before**	**After**	**Before**	**After**
Canada	4.96	4.91	4.95	4.9	4.95	4.91
United States	4.28	4.13	4.28	4.13	4.28	4.13
Costa Rica	2.14	1.05	2.14	1.05	2.14	1.05
Guatemala	1.40	1.30	1.39	1.29	1.38	1.29
Mexico	2.83	2.4	2.83	2.4	2.83	2.4
Brazil	2.08	1.98	2.09	1.99	2.08	1.99
Chile	3.42	2.79	3.42	2.79	3.42	2.79
Ecuador	2.53	1.17	2.54	1.17	2.55	1.2
Paraguay	4.19	4.18	4.23	4.23	4.19	4.18
Estonia	4.53	4.4	4.53	4.4	4.6	4.46
Finland	5.15	5.13	51.4	5.12	5.16	5.13
United Kingdom	3.00	2.87	3.00	2.87	3.00	2.87
Czech Republic	4.21	4.15	4.26	4.2	4.26	4.21
Germany	4.09	4.08	4.11	4.09	4.13	4.11
Romania	4.21	4.14	4.2	4.15	4.21	4.15
Switzerland	4.47	3.65	4.48	3.65	4.48	3.65
Italy	2.81	2.79	2.8	2.79	2.8	2.79
Spain	3.39	2.96	3.39	2.96	3.39	2.96
China	3.14	3.04	3.16	3.02	3.24	3.07
Japan	3.12	2.64	3.12	2.63	3.13	2.64
Korea	3.38	3.1	3.39	3.11	3.39	3.11
Philippines	1.94	1.25	1.94	1.24	1.92	1.23

Taiwan	3.17	2.29	3.17	2.29	3.17	2.29
Vietnam	1.24	1.15	1.24	1.15	1.24	1.15
Australia	2.96	2.92	2.97	2.94	2.97	2.94
South Africa	3.22	2.95	3.22	2.95	3.22	2.95

#2.15. Line 613: Table 2: Could you clarify how similar or close to the observed temperature during the baseline period 2010s, compared to the temperature that averaged across locations within each region under SSP1-2.6, SSP2-4.5, and SSP5-8.5 during the same baseline period?
[Response]
The observed temperature data period varied across countries as presented in Table 1 and some countries did not have observational temperature records for 2010–2019, which is the baseline period for projections. Accordingly, it was not possible to compare between observed and modeled temperatures during the baseline period of the 2010s.

#2.16. Line 384: Do you also assume no adaptation here?
[Response]
Predictions of suicide mortality was modeled under the assumptions of no population changes and no variations in suicide rates. Therefore, assumption of no adaptation was not necessary. We revised the relevant text. (Lines 465-468, page 18, in main text)

> **(Lines 465–468, page 18, in main text)**
> Modeled daily suicide mortality were calculated by averaging the observed suicide data for each calendar day and repeating the daily averages in all years during the projection period (2010-2059), assuming no changes in population structure or suicide rate. Hence, the population and suicide rates from the observed data were used as the baseline

#2.17. Line 405-406: Knots can be found in the supplementary material Table S1 for the justification. How was the lag decided? Please clarify. A concern is whether suicide outcomes occur so quickly within 3 days of temperature exposure?
[Response]
The 3-day lag was selected based on prior epidemiological studies (Thawonmas et al., 2023; Zhou et al., 2023), which indicate that suicide risk is influenced primarily by short-term temperature exposure—typically within 2–3 days—rather than by prolonged lag effects. We revised the text to clarify. (Lines 486–488, page 19, in main text)

In addition, sensitivity analyses using alternative lag periods (3-day vs. 6-day) produced consistent results, supporting the robustness of our findings (see our response to comment #2.21). Therefore, we adopted a 3-day lag to capture these short-term effects based on both previous evidence and empirical validation.

> **(Lines 486–488, page 19, in main text)**

The choice of lag was selected based on prior epidemiological studies[31,32], which indicate that suicide risk is primarily influenced by short-term temperature exposure—typically within 2–3 days—rather than by prolonged lag effects.

References

Thawonmas, R., Hashizume. M. & Kim, Y. Projections of temperature-related suicide under climate change scenarios in Japan. Environ. Health Perspect. 131(11), 117012 (2023).

Zhou, Y. et al. Assessing the Burden of Suicide Death Associated With Nonoptimum Temperature in a Changing Climate. JAMA psychiatry 80(5), 488–497 (2023).

#2.18. Line 423: Also no changes in suicide rate?

[Response]

You are correct. We assumed no change in suicide rate in the projection of suicide mortality attributable to future temperatures. We revised the relevant text. (Line 509-512, page 20, in main text)

(Lines 509–512, page 20, in main text)

The burden of suicide mortality associated with temperatures (i.e., temperatures lower or higher than the median of observed temperatures) was projected to compare the impact of climate change on suicide across regions, assuming no adaptation or population changes, or variations in suicide rates, as previously described[26,31,32,63,65] (see Section 2.2.1 in the Supplementary Material).

#2.19. Line 429: The sentence regarding "highest minimum temperatures... near the median of current temperatures" is unclear. Does this imply that the lowest minimum temperatures were not near the median? Please clarify.

[Response]

We clarified the relevant text. (Lines 518–526, page 20, in main text) We examined where the projected minimum temperatures in the 2050s are positioned within the historically observed temperature distribution for all locations. In Fortaleza, Brazil, the corresponding temperature percentile was the highest among all locations, approximately at the 50th percentile (i.e., the historical median). Thus, in other locations, the projected minimum temperatures were positioned below the median of the historically observed temperature distribution.

(Lines 518–526, pages 20, in main text)

To ensure reasonable future projections, the reference temperature was selected within a range of temperatures expected to exist in the future, enabling consistent and meaningful comparisons of suicide risk across different time periods. In this study, the 50th percentile (median) of the observed temperature distribution at each location was selected as the reference, as it is likely to remain within the projected temperature range in the 2050s even under the most extreme scenario (SSP5-8.5). For example, in Fortaleza, Brazil, the lowest projected temperature in the 2050s under SSP5-8.5 is 27.6°C, which coincides with its observed median temperature (27.6 °C), indicating that future temperatures are expected to begin at levels that historically correspond to the median temperature (Table S6, Fig. S6).

#2.20. Line 438: What is "decal ANs"? Please clarify.

[Response]

We corrected the phrase to "region-specific decadal ANs". (Lines 534, page 21, in main text)

**(Lines 534, page 21, in main text)**<br>The region-specific decadal ANs were

#2.21. Line 447: Were any sensitivity analyses conducted, for example, using different lags or temperature metrics?

[Response]

We conducted sensitivity analyses using an extended lag of 6 days in addition to the main 3-day model (Lines 585-592, page 23, in main text) and reported the results in Fig. S11 (Lines, 433-437, page 30, Supplementary Materials) where the results were roughly consistent between the two lag choices. Regarding the temperature metrics, only daily mean temperature was available for modeled temperature data.

(**Lines 585–592, page 23, in main text)**

Sensitivity analysis

To assess the robustness of our results, a sensitivity analysis was conducted, referring to previous projection studies[20,31,32]. Due to data accessibility limitations, the sensitivity analysis was not performed remotely; instead, it was conducted using data from 426 locations included in this study. Since temperature can influence suicide risk over short-term periods and prior epidemiological studies[31,32] have adopted a 3-day lag, we compared only 3-day and 6-day lag structures. Temporal variations in total attributable fraction (AF) were similar between the two settings, indicating robustness to lag selection (Fig. S11).

Fig. S11 | Sensitivity analysis using short-term lag structures (3-d and 6-d lag).

Redacted

#2.22. Lines 178, 188: Please add line spacing between paragraphs.
[Response]
We added line spacing between paragraph throughout the *Discussion* section.

#2.23. Line 228: Please clarify the top five regions referenced. Are they Central America, South Africa, South Europe, Southeast Asia, and Northern Europe? If so, why are Australia or South America excluded, especially if they experience relatively small temperature changes?
[Response]
We clarified that the five regions referenced are Central America, South Africa, Southern Europe, Southeast Asia, and South America, which were the top five regions with respect to the largest differences in total attributable fraction (AF). (Lines 267-269, page 11, in main text)

> **(Lines 267–269, page 11, in main text).**
> The five regions exhibiting the largest increases in total AF—Central America, South Africa, South Europe, South-East Asia, and South America—are generally warmer. These regions are projected to show moderate temperature increases of about 1.5–2°C and larger sums of RRs.

#2.24. Line 230: The phrase "showing small or large temperature changes" is confusing, not very meaningful. Please clarify.
[Response]
To avoid confusion, we deleted the phrase. (Lines 269-272, page 11, in main text)

> **(Lines 269–272, page 11, in main text).**
> In contrast, the remaining regions—North Europe, North America, Central Europe, East Asia, and Australia—were colder or temperate, showing smaller increases in total AF mainly because of their smaller sums of the RRs.

#2.25. Line 231: Is it better to show results from some sensitivity analyses here to demonstrate the robustness of your study?
[Response] As mentioned in our response to comment #2.21, we conducted sensitivity analyses focusing on different lag structures to assess the robustness of our results (Lines 585–592, page 23, in main text). The analyses compared 3-day and 6-day lag models and produced consistent results in both the direction and magnitude of associations. The corresponding figure is provided in the Supplementary Materials (Fig. S11).

#2.26. Line 234: The study could be multiple countries study. I find it difficult to see this is a "global" study which missed most countries in these important regions such as Africa, the Middle East, Southeast Asia, and East Asia where the regions comprising over 40% of the global population. This study provides the first multiple countries-scale projection of suicide mortality
[Response]

We agreed with the reviewer's comment and replaced the term "global" with "large scale multi-country projection", (Line 278, page 11, in main text)

(**Lines 278, page 11, in main text)**
This study provides the first large scale multi-country projection of suicide mortality burden

#2.27. Lines 276-289: These stressors can adversely affect mental health and contribute to rising suicide rate. It would be better to add contrasting statement and discussion to support this statement from both stressors and enablers, e.g. in Australia you showed the mild increase. However, my previously reviewed studies in Australia e.g. https://doi.org/10.1016/j.uclim.2021.101028 showed high mental health morbidity and cost due to climate change. Do such findings support or conflict with your study? Whether the high morbidity and cost due to climate change in Australia with higher average GDP to support your study on mitigating mortality despite increased morbidity? Please discuss.

[Response]

Thank you very much for your valuable comment. We also appreciate you sharing the previous Australian study (Tong, M et al., 2021, Urban Climate) reporting that climate change may substantially increase mental health morbidity and related healthcare costs. As you noted, interpreting such findings in relation to our results is very important.

However, our study does not aim to cover a broad range of mental health indicators; rather, it focuses on suicide as a specific mental health outcome, with particular emphasis on describing and interpreting the regional heterogeneity in projected increases in temperature-attributable suicide burden under climate change. Therefore, we believe that adding the requested discussion—linking the projected increases in mental health morbidity and healthcare costs in a particular country of Australia to our findings of regional heterogeneity in future suicide burden under climate change—may be beyond the scope of the current study. Nevertheless, the study you brought to our attention is highly relevant to our work and we have citied it in the relevant section of the manuscript. (Lines 131-133, page 6, in main text) If the reviewer considers it essential, we would be happy to further revise the manuscript in the next round of revision.

(**Lines 131-133, page 6, in main text)**
Notably, rising global temperatures and increasing extreme weather events have been linked to various adverse health outcomes, including mental health disorders[3–7]
**Reference**
7. Tong, M. et al. Heat-attributable hospitalisation costs in Sydney: Current estimations and future projections in the context of climate change. Urban Climate 40, 101028 (2021).

Supplemental information

#2.28. Line 21: Data in some countries did not cover the baseline period for the 2010s. Based on the data, how to generate the baseline associations? Please clarify.

[Response]

As clarified in our response to comment #2.10, the baseline temperature–suicide associations were not estimated using modeled data from the 2010s; instead, they were derived from each country's available observed data period. These associations were then linked with modeled daily mean temperature data for 2010–2059 and extrapolated to cover the full range of the temperature distribution through 2059.

In this study, the term "baseline (2010s)" refers to the reference decade used to calculate decadal differences in the total attributable fraction (AF), rather than the empirical observation period used to estimate the temperature–suicide associations.

#2.29. Line 258: Font bold?
[Response]
We removed the bold formatting. (Line 324, Supplementary Materials)

#2.30. Line 318: Changing "Reference" to "References"?
[Response]
We changed "Reference" to "References". (Line 438, Supplementary Materials)

Comments from Reviewer 3

#3.1. Line 105 - For clarity, I would use the term ambient temperature.

[Response]

We added the term "ambient" to clarify the description (Line 108, page 5, in main text).

(**Line 108, page 5, in main text)**<br>Several studies have highlighted a relationship between ambient temperature and mental health,

#3.2. Line 157 - It would be more logical to keep the order of the scenarios according to radiative pressure or effective temperature, i.e. SSP1-2.6, SSP2-4.5, SSP5-8.5, especially since these are the same scenarios as in the submitted paper.

[Response]

We revised as suggested "0.6% for SSP1-2.6, 1.3% for SSP2-4.5, and 2.4% for SSP5-8.5" (Lines 174-176, page 7, in main text).

**(Lines 174–176, page 7, in main text)**<br>In the 2090s, the projected increases compared to the 2010s were estimated under different shared socioeconomic pathway (SSP) scenarios, 0.6% for SSP1-2.6, 1.3% for SSP2-4.5, and 2.4% for SSP5-8.5,

#3.3. Line 160 – I would prefer: the "extent of the correlation" instead of "the strength of … relationship"

[Response]

We replaced the phrase "the strength of the relationship" by "the extent of the temperature–suicide association". (Line 184, page 8, in the main text)

**(Line 184, page 8, in main text)**<br>This study aimed to (1) determine the extent of the temperature–suicide association based on observed data,

#3.4. Line 172 – "…of Korea.", one could add: due to long time coverage and high population.
and the Republic of Korea due to long time coverage and high population.

[Response]

We added the phrase "due to long time coverage and high suicide rate." (Lines 196-198, page 8, in main text),

**(Lines 196–198, page 8, in main text)**<br>The total number of suicides in the Multi-Country Multi-City (MCC) dataset was largely contributed by Japan and the Republic of Korea due to long time coverage and high suicide rate.

#3.5. Line 193 / 260 – This finding, that the positive correlation between ambient temperature and suicides in East Asia and Australia practically disappears above a certain threshold, is very surprising at first glance. Can you think of any explanations for this?

[Response]

We added text to the relevant paragraph in the *Discussion* section to explain this pattern. (Lines 308–312, page 12, in main text) The inverse J-shaped temperature–suicide relationship observed in East Asia and Australia may be explained by several mechanisms, including behavioral and physiological adaptation to heat, reduced physical activity and impulsivity during extreme heat, and the effects of public health interventions such as heat-health warning systems and increased public awareness.

> **(Lines 308–312, page 12, in main text)**
> Notably, East Asia and Australia demonstrated inverse J-shaped temperature–suicide relationships, where suicide risk increased with temperature up to a certain point and then plateaued or slightly declined at extreme heat levels. This pattern may reflect behavioral and physiological adaptation to high temperatures among populations regularly exposed to hot conditions (e.g., greater use of cooling systems and modified daily activities)[34].

#3.6. Line 207 to 213 – Even after reading it several times, I still find this section hardly comprehensible. Elsewhere, it is argued that higher temperatures lead directly and indirectly to increased suicide mortality. At this point, if I understand correctly, we are talking about the contribution of low temperatures in the context of general global warming to suicide mortality. Does that mean you want to reflect the effects of temperature extremes in both directions? If that is the case, it should be introduced in a more comprehensible manner.

[Response]

We acknowledge that our explanation in this paragraph was insufficient, and we sincerely appreciate your comment bringing this issue to our attention.

As you correctly understood, the temperature–suicide relationship is generally monotonic, meaning that higher temperatures increase suicide risk. Accordingly, when estimating the temperature-attributable suicide burden, it is common to define the reference temperature as the minimum suicide temperature (MinST), often corresponding to the lowest temperature within the observed temperature distribution, and to quantify the excess number of suicides attributable to temperatures above this reference.

However, in this study, our goal was to project future temperature-related suicide burdens. If we were to use the minimum temperature in the current temperature distribution as the reference, this temperature would be unlikely to occur under future climate conditions. Therefore, we instead selected a reference temperature that is expected to remain within the future temperature distribution, specifically the 50th percentile of the current temperature distribution, which corresponds to the minimum temperature in the projected future temperature distribution. As a result, when computing the temperature-attributable suicide burden, we also quantified the avoided suicides associated with temperatures below this reference temperature in addition to the excess suicides associated with temperatures above the reference. That is, we quantified temperature effects in both directions.

A more detailed explanation is provided in the main text (Lines 540–554, page 21, in main text), and we kindly refer the reviewer to our responses to comments #4.1.1 and #4.2 for further clarification.

(Lines 540–554, page 21, in main text)
To put it more concretely, we examined the temporal changes in AF (%) of suicide mortality associated with temperatures above and below the reference. Because the reference temperature corresponds to the median of observed temperature distribution, the total AF can be decomposed into contributions from temperatures below and above this reference**.** For this decomposition, daily attributable numbers were separately aggregated for days with temperatures above and below the reference at each location and summed by region, SSP, and decade. These aggregated values were then divided by the total number of suicide deaths in each region to obtain AFs for warm and cold temperatures, respectively. Accordingly, regional changes in total AF were decomposed into contributions from warm (above-reference) and cold (below-reference) temperatures (Fig. S7). While the relative contributions varied by region and location, AFs associated with both warm and cold temperatures increased over time and more intense warming, especially SSP5-8.5. The increase in AF associated with warm temperatures reflects a rise in excess suicide mortality at higher-than-reference temperatures. By contrast, the rise in AF for cold temperatures results from a diminishing protective effect of lower-than-reference temperatures, reducing the contribution of these lower temperatures that would otherwise offset excess suicide mortality.

#3.7. Line 238 – In any case, it is worth explaining why these three SSPs were selected.
[Response]
We added an explanation for these three climate scenarios. (Lines 441-444, page 17, in main text)
Specifically, SSP1-2.6, SSP2-4.5, and SSP5-8.5 were chosen to represent a broad and distinct range of greenhouse gas emission trajectories—from strong mitigation (SSP1-2.6) to intermediate stabilization (SSP2-4.5) and high-emission (SSP5-8.5) scenarios. These SSPs are widely used in climate impact assessments to capture different degrees of future climate change.

(Lines 441–444, page 17, in main text)
SSP1-2.6, SSP2-4.5, and SSP5-8.5 scenarios represent combined projections of future socioeconomic development and greenhouse gas emissions, ranging from mitigation (SSP1-2.6) to intermediate stabilization (SSP2-4.5) and high-emission (SSP5-8.5) scenarios[57]. These SSPs are widely used in climate impact assessments to capture varying degrees of future climate change.

#3.8. Line 251 / 253 – "We observed regional variations in future … suicide mortality", "…the next five decades…" - Since we are in the year 2025 and the projection extends to 2059, this represents three and a half decades.
[Response]
We replaced the phrase "the next five decades" with "by the 2050s" to accurately reflect the projection period. (Line 302, page 12, in main text)

(Line 302, page 12, in main text)
steep increases were projected by the 2050s in all climate change scenarios.

#3.9. Line 267 to 280 - This section seems very important to me. Later on, possible ways in which increased temperatures can directly influence the suicide rate are discussed (lines 290 to 304). However, according to the literature, these effects are likely to be secondary in nature. Suicide is a very complex phenomenon with multiple causes. Decades of quantitative research have identified various risk factors that can be classified at the individual level into biological, psychological, clinical and social factors, such as the presence of mental illness, substance abuse, predisposed personality traits such as impulsivity, delinquency, poverty, loneliness, stressful life events, previous trauma, belonging to an ethnic minority or having a different sexual orientation or identity. In addition, environmental factors that affect the population as a whole are also significant. In particular, the influence of economic hardship has been robustly documented retrospectively. Since economically less developed countries in particular suffer from the consequences of climate change, these indirect effects are likely to be more significant in the end.

[Response]

We appreciate this valuable comment for discussing the individual- and environmental-level factors that may directly and indirectly influence suicide. We find your insightful comment very helpful, particularly regarding the possibility that various individual-level factors and broader environmental factors may have a more direct influence on suicide, whereas physiological or biological mechanisms may play a secondary role. We have revised the relevant paragraph to better reflect these points. (Lines 322–334, page 13, in main text) If any parts remain insufficient, we would be happy to make further revisions accordingly.

(Lines 322–334, page 13, in main text)

Another potential explanation for regional variations in future temperature-related suicide mortality may be differences in socioeconomic conditions. Except for South Europe, the warmer regions that showed sharp increases in suicide mortality associated with temperatures over time often overlapped with countries of relatively low socioeconomic status (based on average GDP, Table 1). In contrast, regions where suicide risk increased more gradually tended to comprise countries with higher average GDP. These findings suggest that the impact of climate change on suicide mortality may mediated indirectly by regional disparities in economic stability and social infrastructure. Climate change can exacerbate existing inequalities, as vulnerable populations and low-income countries face greater economic instability35,36, escalating conflicts37-39, and declining agricultural productivity/food security40. Moreover, in regions with limited access to mental health care and underdeveloped infrastructure, the lack of timely intervention and support may heighten the burden on vulnerable populations36. Therefore, the link between rising temperatures and suicide mortality may reflect, at least in part, the indirect consequences of socioeconomic hardship amplified by climate change.

#3.10. Line 317 – The rising average age of the world's population will almost certainly have an impact on suicide behavior, as suicide, unlike suicide attempts, becomes much more likely with age.

[Response] We strongly agree with the reviewer. We therefore acknowledged not considering the population aging in our study is one of the limitations. We have added an explanation of the potential impact on the results if this aspect is not considered. (Line 374-379, page 15, in main text)

(Line 374–379, page 15, in main text)

Several limitations should be acknowledged in this study. First of all, in order to isolate the changes solely due to climate change, our projections were based on simplified assumptions, including constant suicide rates, no human adaptation, and a static population. As such, we did not account for population aging, despite evidence that suicide rates tend to increase among individuals aged 60 years and older[52]. We used a fixed baseline suicide rate and did not account for potential temporal changes in baseline suicide rates.

Reference

52. Shah, A. et al. Suicide rates in five-year age-bands after the age of 60 years: the international landscape, Aging & Mental Health, 20(2), 131–138 (2016). https://doi.org/10.1080/13607863.2015.1055552

#3.11. Line 336 – Another limitation, in my view, is that reporting on suicides in less developed countries is considered unreliable and many suicides are misclassified, whether for cultural or religious reasons or due to inadequate post-mortem examinations. Even in high-income countries, a certain degree of underreporting is assumed.

[Response]

We strongly agree with the reviewer. We added text in the *Discussions* section (Lines 403–406, page 16, in main text). This addition clarifies that differences in data reliability and underreporting should be considered when interpreting regional suicide mortality.

(Lines 403–406, page 16, in main text).

Lastly, the quality of suicide data varies countries substantially across countries.[12] Globally, suicide underreporting—driven by poor certification systems, social stigma, and in some settings the illegality of suicidal behavior—is estimated at about 18%, exceeding 30% in low- and middle-income countries.[12,53]

References

12. WHO. Suicide Worldwide in 2021: Global health estimates (WHO, 2021).

53. Meda, N. et al. How many people die by suicide each year? Not 727,000: a systematic review and meta-analysis of suicide underreporting across 71 countries over 122 years. Front. Psychiatry 16, 1609580 (2025).

#3.12. Fig. S1 – A larger cloud of dots appears here in southern Pakistan, but this does not seem to be part of this investigation.

[Response]

We corrected and replaced the figure to remove the extraneous data points in southern Pakistan (Fig. S1, Supplementary Materials). Please see our response to comment #1.14 for details.

Comments from Reviewer 4

#4.1. Choice of reference temperature and exposure–response shape (DLNM)

#4.1.1 The analyses set the suicide relationship at each location's 50th percentile of temperature distribution as the reference the temperature. This is not generally appropriate. In time-series studies using DLNMs, the standard practice is to set the reference temperature at the Minimum Mortality/Morbidity Temperature (MMT)—the temperature at which the relative risk (RR) is minimized (e.g., Gasparrini 2011; Peng et al. 2017; Zhou et al. 2023; Liang et al. 2024). The temperature higher or lower the MMT will cause relative risk greater than 1, which means the harmful risks cause by both hot and cold temperature. Setting the median as the reference temperature implies that temperatures below the 50th percentile often show RR < 1 across wide ranges, which is visible in Fig. 1 and is epidemiologically counter-intuitive. It doesn't make sense that all the exposure-response curve show relative risk lower than 1, i.e. no risks for the temperature lower the 50th percentile. I suggest the authors re-estimate the location-specific DLNMs following standard procedures: (i) identify the MMT for each location, (ii) re-center the cross-basis at the MMT, and (iii) compute RRs on the absolute temperature scale (°C), not percentiles.

[Response]

We sincerely thank the reviewer for the insightful comments regarding the choice of reference temperature and the centering of the exposure–response function in the DLNM framework. After careful consideration, we decided to retain the 50th percentile of each location's observed temperature distribution as the reference temperature for the following reasons.

In studies of the association between temperature and all-cause mortality, it is appropriate to define the reference temperature as the minimum mortality temperature (MMT) for future projections. The temperature–mortality relationship typically shows a U-shaped pattern, with the MMT located near the middle of the observed temperature distribution. Therefore, the MMT, which is within the currently observed temperature range, is highly likely to be observed in the future.

However, in studies of the temperature–suicide relationship, the association is generally monotonically increasing. In this case, if the reference temperature is defined as the minimum suicide temperature (MinST, like MMT), this reference temperature is highly unlikely to occur in future climate conditions. Consequently, when projecting future suicide burdens attributable to temperature, using the MinST as a reference temperature raises a conceptual issue: we project temperature-related suicide mortality based on a temperature value that is unlikely to be observed in future temperature distributions.

Therefore, to enhance comparability across regions and periods, we chose the reference temperature as the one which will remain within a plausible future temperature range, even under the most extreme scenario (SSP5-8.5). This approach provides a consistent benchmark across all locations and minimizes extrapolation uncertainty that may arise when the MinST lies outside the projected future temperature range. Also, defining the reference temperature as one that is likely to occur in future periods enables attributable fractions (AFs) and attributable numbers (ANs) to be interpreted consistently and compared across decades. Following this principle, a recent study projecting the temperature-related suicide burden in Japan selected

the 25th percentile of the observed prefecture-specific temperature distribution as a reference. (Thawonmas R et al., 2024).

Nevertheless, we sincerely respect the reviewer's valuable suggestion, as the choice of the reference temperature, particularly the MinST, is indeed important and may raise questions among readers. Therefore, we have provided additional results using MinST as a reference in the *Methods* section (Lines 569–583, page 22, in main text).

We re-estimated the exposure–response functions using the MinST as the reference temperature. The results (Figure S10, Supplementary Materials) show that the RR curve estimates centered at the MinST (top panel) yielded consistent directions with results in Fig 1. In addition, the decadal trends in changes in total AFs relative to the 2010s across scenarios and regions (bottom panel, Figure S10) is consistent with results in Fig 2. Notably, the uncertainty in total AFs was larger when using the MinST as the reference.

Finally, we computed RRs on the absolute temperature scale (°C), and the percentile scales in the figures were used solely for visualization purposes to allow for comparable display of temperature ranges across regions.

(Lines 569–583, page 22, in main text)

Using the minimum suicide temperature (MinST) as reference temperature

We compared our results with those obtained using the reference temperature defined as the temperature corresponding to the lowest suicide risk—the minimum suicide temperature (MinST) in the exposure–response curve (see Fig. S10 in the Supplementary Material). To avoid bias from extreme outliers, the MinST was identified within the 1st to 99th percentile range of observed daily temperatures, which in practice corresponded to the minimum temperature in most locations (top panel in Fig. S10). The overall shapes of the temperature–suicide association curves were similar to those obtained using the median temperature as the reference (top panel in Fig. S10; Fig. 1); however, using the MinST as the reference resulted in wider relative risk estimates at higher temperatures (top panel in Fig. S10). Using the MinST -based reference, temporal changes in total AF across future decades remained largely consistent with those obtained using the median-temperature reference (bottom panel in Fig. S10; Fig. 2). Although the absolute values of total AF varied slightly depending on the choice of reference temperature, AF estimates based on the MinST reference exhibited wider uncertainty across regions. Overall, the minimal differences observed between the two reference settings support the robustness of the results and the appropriateness of using the median temperature as the reference in the main analyses.

Reference

Thawonmas, R., Hashizume. M. & Kim, Y. Projections of temperature-related suicide under climate change scenarios in Japan. Environ. Health Perspect. 131(11), 117012 (2023).

Fig. S10 | Temperature-suicide associations and change in total attributable fractions using the minimum suicide temperature (MinST) as the reference.

Redacted

The top panel shows temperature–suicide relationships re-estimated using the MinST as the reference, with the MinST representing the temperature corresponding to the lowest suicide risk within the 1st–99th percentile range of observed temperatures. The bottom panel shows the decadal changes in total AF relative to the 2010s with 95% eCIs across regions and future decades, as recalculated using the MinST-based reference. MinST, minimum suicide temperature; AF, attributable fraction; eCIs, empirical confidence intervals.

#4.1.2 Please report the lag structure, spline bases/knots, and criteria used for model selection.

[Response]

In the first stage, the lag–response association was parameterized using dummy variables to define intervals for lag 0 and lags 1–3. The exposure–response association was modeled using a natural cubic spline with a single internal knot positioned at the 50th percentile of the observed temperature distribution for each location. The number and placement of knots were determined based on quasi–Akaike's Information Criterion (QAIC) values (Table S4). As previous projection studies commonly used a 3-day lag, we used 3-day lag but compared models with 3-day and 6-day lags to determine an appropriate short-term lag. Detailed model selection criteria are provided in the *Methods* section of the Supplementary Materials. (Lines 170-176, page 7, Supplementary Materials)

In the second stage, the model with the lowest I^2 statistic (6.7%) was selected, indicating minimal residual heterogeneity in the meta-analysis (Table S5) (Lines 202–203, page 8, Supplementary Materials).

(Lines 170–176, page 7, Supplementary Materials)
The exposure-response association was modeled using a natural cubic spline with a single internal knot positioned at the 50th percentile of the observed temperature distribution for each location.[8] The lag-response association was parameterized using dummy variables to define intervals for lag 0 and lags 1–3, capturing the short-term delayed effects of temperature on suicide mortality.[8] The number of knots and its locations were determined based on quasi Akaike's Information Criterion (QAIC) values in Table S4. As previous projection studies commonly used a 3-day lag, we compared 3- and 6-day lags to determine an appropriate short-term lag.

(Lines 202–203, Supplementary Materials)
The model with the lowest I^2 statistic (6.7%) was selected, indicating minimal heterogeneity in the meta-analysis (see Table S5).[11]

#4.2. Implications for attributable fractions and numbers (AF/AN)
With the current centering, temperatures below the 50th percentile yield RR < 1, producing negative AF (AF=(RR-1)/RR×100). The net effect then becomes a cancellation between positive AF (hot side) and negative AF (cold side), obscuring true burdens. This further highlights the problems of defining the exposure–response function by using the 50th percentile temperature as the reference point, as noted in my first comment. Therefore, the projection of impact of climate change on suicide should be redone based on the new exposure response function established as I suggested above. Therefore, I would recommend the authors to compute and present hot- and cold-attributable burdens separately as well as the net total, after recalculated the exposure-response function using MMT. This is standard practice and avoids misleading cancellations.
[Response]
We sincerely thank the reviewer for this important comment. As the reviewer correctly noted, when the 50th percentile temperature is used as the reference, some temperature ranges yield RR < 1, resulting in negative attributable fractions (AFs). The concepts of attributable number (AN) and attributable fraction (AF) are defined relative to the chosen reference temperature, representing variations in mortality associated with deviations from that reference. Hence, relative risks (RRs) below 1 at lower temperatures do not imply the absence of risk but rather may indicate relatively lower risk compared with the median reference.

These negative AFs may partially offset the positive AFs at higher temperatures, potentially leading to misinterpretation of the net burden. However, as our interpretation focuses on decadal changes in total AFs relative to the 2010s, we emphasize that both warm- and cold-related contributions increase over time when compared with the baseline decade of 2010s (Figure S7). This approach minimizes potential misinterpretation caused by offsetting effects between positive and negative AFs.

We clarified in the text that negative AF values associated with colder temperatures should be interpreted as protective effects relative to the reference temperature (i.e., avoided suicides due to temperatures below reference temperature), rather than as the absence of risk (Lines 540–556 in the main text; Lines 250–256, page 9, Supplementary Materials).

(Lines 540–556, page 21, in main text)

To further understand the underlying components of these changes, we examined the temporal changes in AF (%) of suicide mortality associated with temperatures above and below the reference. Because the reference temperature corresponds to the median of observed temperature distribution, the total AF can be decomposed into contributions from temperatures below and above this reference. For this decomposition, daily attributable numbers were separately aggregated for days with temperatures above and below the reference at each location and summed by region, SSP, and decade. These aggregated values were then divided by the total number of suicide deaths in each region to obtain AFs for warm and cold temperatures, respectively. Accordingly, regional changes in total AF were decomposed into contributions from warm (above-reference) and cold (below-reference) temperatures (Fig. S7). While the relative contributions varied by region and location, AFs associated with both warm and cold temperatures increased over time and more intense warming, especially SSP5-8.5. The increase in AF associated with warm temperatures reflects a rise in excess suicide mortality at higher-than-reference temperatures. By contrast, the rise in AF for cold temperatures results from a diminishing protective effect of lower-than-reference temperatures, reducing the contribution of these lower temperatures that would otherwise offset excess suicide mortality. The spatial distribution of these decomposed results for each location is shown in Fig. S8 for warm temperatures (above the reference temperature) and Fig. S9 for cold temperatures (below the reference temperature).

(Lines 250–256, page 9, Supplementary Materials)

Conceptually, the AF represents the proportion of suicide burden at a given temperature that is attributable to deviations from the reference temperature, while the AN quantifies the number of suicide deaths due to temperature changes compared with the total number of suicides at that temperature, reflecting how many deaths are estimated to occur (or be prevented) due to temperatures. For instance**,** a positive AF (RR > 1) indicates an increased suicide burden at higher-than-reference temperatures, whereas a negative AF (RR < 1) reflects a relative reduction or protective effect at lower-than-reference temperatures.

(Lines 383–393, page 26, Supplementary Materials)

Fig. S7 | Temporal changes in attributable fraction (AF, %) of suicide mortality associated with both warm and cold temperatures for each decade from the 2010s by region under three climate change scenarios (SSP1-2.6, SSP2-4.5, and SSP5-8.5).

Redacted

#4.3. Report attributable numbers (AN), not only AF

The Methods state that ANs were calculated, but Figs. 2–4 present only AFs. For policy relevance, decision-makers need case counts. The authors should add figures/tables showing AN (with 95% uncertainty intervals), and specify baseline populations and suicide rates used, projection time slices, and how uncertainties from exposure–response functions and climate projections were propagated.

[Response]

We added a new table summarizing ANs with 95% empirical confidence intervals (eCIs) to the Supplementary Material (Table S2). (Lines 299-305, page 13-14, Supplementary Materials) Because of the large inter-regional variability in AN value, graphical representation was less readable.

The total number of suicide deaths during the observed data period is provided in Table 1. However, information on population size and suicide rates was not available and suicide count data rather than rate-based data were used in this study.

We also provided details of the projection periods in *Methods* section (Lines 440–441, page 17, in main text), defined by decade from the 2010s (2010–2019) through the 2050s (2050–2059) under three climate scenarios (SSP1-2.6, SSP2-4.5, and SSP5-8.5).

**(Lines 440–441, page 17, in main text)**<br>The projection periods are defined by decade, starting from the 2010s (2010–2019) and continuing through the 2050s (2050–2059).

To better describe uncertainty propagation, we expanded the *Methods* section (Lines 558–567, page 22, in main text). Both the uncertainty in exposure–response estimation and the variability across different climate model projections were incorporated using a meta-regression–based Monte Carlo simulation (1,000 iterations). For each iteration, temperature time series corresponding to each GCM–SSP combination were applied, thereby reflecting both uncertainty from the exposure–response function and climate model variability.

(Lines 558–567, page 22, in main text)
We quantified the uncertainty in future temperature-related suicide mortality using Monte Carlo simulations at the meta-regression-model level as previously described[64] (see Section 2.2.2 in the Supplementary Material). These simulations provided eCIs that accounted for the correlation among location-specific predictions and uncertainty associated with the spatialization of random effects in the predictive meta-regression model. In addition, uncertainty from climate projections was incorporated by combining temperature estimates from five GCMs under three SSP scenarios, ensuring that the final impact estimates reflect both epidemiological and sources of uncertainty. This approach captures the uncertainty in multiple stages of the analysis—including exposure-response estimation and climate projections—and reduces the risk of underestimation, particularly in analyses involving small-area data and low heterogeneity[64].

#4.4. Scenario coverage in Figs. 3–4
Figs. 3–4 show results only for SSP5-8.5, whereas Fig. 2 includes SSP1-2.6, SSP2-4.5, and SSP5-8.5. Please include all three scenarios in Figs. 3–4 (or provide parallel panels/appendix figures). This will materially improve the paper's mitigation insights.
[Response]
Following the reviewer's suggestion, we expanded the scenario coverage to include all three climate change scenarios (SSP1-2.6, SSP2-4.5, and SSP5-8.5). We provided the additional results for SSP1-2.6 and SSP2-4.5 in Fig. S4 and Fig. S5. (Lines 351-370, page 23-24, Supplementary Materials) We explained this addition in main text. (Line 258, page 10; Line 272, page 11, in main text)

Fig. S4 | Map of projected changes in total attributable fraction of suicide mortality associated with non-reference temperatures between 2010s and 2050s under two climate change scenarios: (A) SSP1-2.6 and (B) SSP2-4.5.

Redacted

Redacted

Fig. S5 | Relative risk at extreme temperatures, projected warming, and change in total attributable fractions of suicide mortality by two climate change scenarios: (A) SSP1-2.6 and (B) SSP2-4.5)

Redacted

Redacted

#4.5. Characterizing extremes

Suicide risks are plausibly more sensitive to extreme heat/cold than to decadal mean temperatures. In Table 2 (and/or a new table), I suggest the authors to add regional statistics for relevant extreme indices (e.g., hot-day/hot-night frequencies, heatwave metrics, TXx/TNn) consistent with the exposure metric used in the DLNM.

[Response]

We strongly agree with the reviewer that suicide risk may be more sensitive to extreme heat and cold than to decadal mean temperatures. However, our study focuses on the association between daily mean temperature and suicide, and thus adding other temperature metrics to Table 2 may make the presentation somewhat less focused. Nevertheless, we believe that examining the relationships between various

temperature indicators and suicide would be highly valuable in future research, and we are currently planning to pursue such work. Thank you for this important suggestion.

#4.6. Discontinuity in Northern Europe in Fig. S3

There appears to be a discontinuity around 2010 between historical and projected temperature changes for Northern Europe. Please check the stitching of historical and future series (bias correction, baseline alignment, ensemble concatenation) and correct the discontinuity.

[Response]

We carefully rechecked the data processing steps, including bias correction, baseline alignment, and ensemble concatenation, and corrected the discontinuity. The revised results are presented in Figure S2. (Lines 337-344, page 21, Supplementary Materials)

Figure S2 | Decadal changes in mean temperature relative to the 2010s by region and climate scenario.

Redacted

#4.7. Model transparency and confounding control

Ensure the main text or supplement clearly states: over-dispersion handling; seasonality/long-term trend control; day-of-week/holiday adjustments; lag length justification; meta-analytic pooling (random-effects), any effect-modification analyses (e.g., by income, baseline suicide rate, health system indicators), and calculation details of AF and AN. This information is essential for reproducibility and interpretation.

[Response]

All requested methodological details are fully documented in the Supplementary Material (Section 2: Details on Statistical Analysis), specifically in Sections 2.1 and 2.2.

The analyses employed a conditional Poisson regression model under a time-stratified case-crossover design, which accounted for over-dispersion, seasonality, long-term trends, and day-of-week effects. (Lines 157-160, page 7, Supplementary Materials)

(Lines 157–160, page 7, Supplementary Materials)
a conditional Poisson regression model[1], taking overdispersion into account, was applied within a time-stratified case-crossover design[2] to control for temporal confounding factors such as long-term trends, seasonal patterns, and day-of-week effects.

The lag structure (maximum 3 days) was justified based on prior literature (Thawonmas et al., 2023; Zhou et al., 2023) and sensitivity analyses comparing 3- and 6-day lags (Lines 585-592, page 22, in main text).

(**Lines 585–592, page 23, in main text)**
Sensitivity analysis
To assess the robustness of our results, a sensitivity analysis was conducted, referring to previous projection studies20,31,32. Due to data accessibility limitations, the sensitivity analysis was not performed remotely; instead, it was conducted using data from 426 locations included in this study (excluding four cities in Australia, fourteen in Germany, and all locations in Finland). Since temperature can influence suicide risk over short-term periods and prior epidemiological studies[31,32] have adopted a 3-day lag, we compared 3-day and 6-day lag structures. Temporal variations in total attributable fraction (AF) were similar between the two settings, indicating robustness to lag selection (Fig. S11).

References
Thawonmas, R., Hashizume. M. & Kim, Y. Projections of temperature-related suicide under climate change scenarios in Japan. Environ. Health Perspect. 131(11), 117012 (2023).

Zhou, Y. et al. Assessing the Burden of Suicide Death Associated With Non-optimum Temperature in a Changing Climate. JAMA psychiatry 80(5), 488–497 (2023).

The location-specific associations were pooled through a multivariate mixed-effects meta-regression with random effects at both the country and city levels, following a hierarchical structure. Effect modifiers such as region, GDP, and mean temperature range were included as meta-predictors, and the model with the lowest heterogeneity ($I^2 = 6.7\%$) was selected. (Lines 196-200, page 8, Supplementary Materials)

(Lines 196–200, Supplementary Materials)
Random effects were incorporated to account for intercept variability at both country and city levels, assuming a hierarchical structure where cities are nested within countries. Meta-predictors were included as fixed effects, such as region, country-level GDP, and location-specific variables (e.g., average temperature and the range of daily mean temperatures during the observed period).

Both attributable fractions (AFs) and attributable numbers (ANs) were calculated using location-specific temperature–suicide associations, baseline population data, and modeled suicide counts for each decade and climate scenario. Detailed descriptions of the computational procedures, including the relevant equations, parameter definitions, and modeling assumptions, are provided in the Supplementary Materials. (Lines 243–269; pages 9-10, Supplementary Materials)

(Lines 243–269, Supplementary Materials)

Using these reference temperatures, the attributable number (AN) and fraction (AF) of suicide mortality were then calculated for each location and for each combination of the three Shared Socioeconomic Pathways (SSPs) and five General Circulation Models (GCMs). 4,5 The AF and AN were computed using the following formulas:

$\mathrm{AF}_{T,t} = 1 - e^{(-f(T_t;\eta))}$, $\mathrm{AN}_{T,t} = \mathrm{AF}_{T,t} \cdot D_t$

where D_t represents the number of suicide deaths at time t. These values indicate the number ($\mathrm{AN}_{T,t}$) and fraction ($\mathrm{AF}_{T,t}$) of suicide deaths at time t attributable to temperatures during the lag period, relative to the reference temperature. Conceptually, the AF represents the proportion of suicide burden at a given temperature that is attributable to deviations from the reference temperature, while the AN quantifies the number of suicide deaths due to temperature changes compared with the total number of suicides at that temperature, reflecting how many deaths are estimated to occur (or be prevented) due to non-reference temperatures. For instance, a positive AF (RR > 1) indicates an increased suicide burden at higher-than-reference temperatures, whereas a negative AF (RR < 1) reflects a relative reduction or protective effect at lower-than-reference temperatures.

Next, we calculated the daily number of attributable suicide deaths $D_{i,t}^{attr}$ for each of the days with modeled temperatures $T_{i,t}^{mod}$ as follows:

$$D_{i,t}^{attr} = D_{i,t}^{mod}\left[1 - e^{-\left(f(T_{i,t}^{mod};\hat{\theta}_i^b) - f\left(T_i^{ref};\hat{\theta}_i^b\right)\right)}\right]$$

where $D_{i,t}^{mod}$ is modeled suicide deaths and $\hat{\theta}_i^b$ denotes the reduced coefficients from the second stage modeling. As the temperature-suicide association is monotonically increasing, $D_{i,t}^{attr}$ is positive/negative for the days when the modeled temperature $T_{i,t}^{mod}$ is higher/lower than the reference temperature T_i^{ref}. Subsequently, for each location, we aggregated $D_{i,t}^{attr}$ across all days within each decade to calculate location-specific decadal attributable numbers (ANs). These ANs were then pooled by region and across SSPs. Ensemble averages across GCMs were calculated for the pooled ANs to obtain the projected region-specific decal ANs under each climate scenario.

Finally, total attributable fractions (AFs) are calculated as the ratio of the GCMs-ensemble ANs to the total modeled suicide deaths within the corresponding period in each region.[4-7,12] This fraction provides a measure of temperature-related suicide mortality under climate change.

#4.8 The manuscript would benefit from professional English editing to improve clarity and readability.

[Response]

The revised version has undergone professional English editing by Editage, and we have made every effort to further improve the overall clarity, coherence, and readability of the text.

Dear Editor,

We sincerely thank you and the reviewers for your careful re-evaluation of our revised manuscript entitled “Projections of temperature-related suicide mortality under climate change: A multi-country time-series study.”

We are grateful for the reviewers’ positive assessments and constructive suggestions during this second round of review. In response to the comments, we have further strengthened the discussion of limitations and incorporated recent relevant literature in the Introduction. The manuscript has been revised accordingly.

The following pages provide detailed point-by-point responses to the reviewers’ comment, along with descriptions of the corresponding revisions made in the manuscript. Reviewer comments are presented in bold, followed by our responses.

Comments from Reviewer 1

#1. I have carefully read the authors' responses to all reviewer comments, particularly those addressing my own points. I am satisfied that my concerns have been fully addressed, and I have no further suggestions for revision. The manuscript has been strengthened through the revision process, excellent work.

[Response]

We thank the reviewer for the positive feedback. We greatly appreciate your constructive comments, which have helped improve the manuscript.

Comments from Reviewer 2

#2. Methods: Lines 440–444 indicated that temperature projections were obtained under three SSP scenarios, representing combined projections of future socioeconomic development and greenhouse gas emissions, ranging from mitigation (SSP1-2.6) to intermediate stabilization (SSP2-4.5) and high-emission (SSP5-8.5) scenarios. However, lines 467–468 stated that the projections assume no changes in population structure, and lines 475–476 further indicated no adaptation and no change in population structure. Given that SSP scenarios inherently consider and incorporate assumptions about future population dynamics and adaptation, there appears to be some inconsistency between the use of SSP scenarios and the assumption of no population change and no adaptation. If the authors have used SSPs solely as sources of temperature projections, this should be more clearly stated and justified. Alternatively, the authors may consider using RCP-based temperature projections.

[Response]

We thank the reviewer for raising this important point. We agree that SSP pathways inherently incorporate assumptions regarding future socioeconomic development, demographic change, and adaptation, whereas our projections of temperature-related suicide burden assumed no changes in population structure and no adaptation. This creates a conceptual inconsistency between the use of SSP scenarios for temperature projections and the simplifying assumptions applied in projecting future temperature-related suicide burden.

The most ideal approach for assessing the future suicide burden attributable to climate change would be to use SSP-based future temperature projections while simultaneously accounting for changes in population structure and potential future adaptation, thereby estimating the temperature-attributable suicide burden under more realistic future conditions. However, while obtaining SSP-based future temperature projections is relatively straightforward, projecting future changes in population structure and assessing how adaptation may alter the temperature–suicide relationship across multiple countries involves substantial uncertainties and methodological challenges.

Therefore, in the present study, we incorporated the former component (SSP-based temperature projections) but did not account for the latter (changes in population structure and adaptation). Consequently, the projected estimates in the current study should be interpreted as scenario-based approximations rather than precise predictions. This limitation was already discussed as one of the major limitations of our study in the Discussion section. In response to the reviewer's comment, we have further revised the manuscript to clarify the potential inconsistency arising from the use of SSP-based temperature projections while assuming no changes in population structure or adaptation in the projection of suicide burden. (Lines 374–389, page 15, in main text)

With regard to the reviewer's suggestion that RCP-based temperature projections could be used instead, given that the analysis assumes no changes in population structure or adaptation, we used SSP-based projections because they represent the current standard within CMIP6 climate models, whereas RCP scenarios are associated with earlier CMIP5 simulations. SSP pathways diverge from around 2015 onward and incorporate more recent emissions trajectories, making them the prevailing framework in contemporary climate impact research. (References 24, 25, 27, 31, 32 in main text)

Taken together, we believe that this study lays the groundwork for future global assessments of climate change–related suicide burden based on multi-country, multi-city data. For more realistic projections of the future health burden attributable to climate change, it will be essential to incorporate changes in population structure and potential adaptation processes in addition to SSP-based temperature projections. The authors are currently planning follow-up research to address these aspects and to provide more comprehensive projections in future work.

(Lines 374-389, page 15, in main text)
Several limitations should be acknowledged in this study. To isolate the effects attributable solely to climate change, our projections relied on several simplifying assumptions. First, baseline suicide rates were assumed to remain constant over time, although suicide trends are influenced by evolving socioeconomic conditions, mental health services, and prevention policies. Second, the exposure–response relationship between temperature and suicide was assumed to remain unchanged, implicitly representing a no-adaptation scenario, whereas behavioral, technological, and infrastructural adaptation to heat may modify temperature-related risks. Third, population size and demographic structure were assumed to remain static, ignoring potential changes due to population growth, aging, and migration. These assumptions also create a conceptual inconsistency with the use of SSP-based temperature projections, as SSP pathways inherently incorporate assumptions regarding future socioeconomic development, demographic change, and adaptation, while our projections of temperature-related suicide burden assumed no changes in population structure and no adaptation. Because all these factors may substantially influence future suicide burdens in complex way, the current projected estimates should be interpreted as scenario-based approximations rather than precise predictions. Future studies should therefore incorporate demographic dynamics, temporal change in suicide trends and potential adaptation when projecting temperature-related suicide burden to produce more realistic estimates.

Comments from Reviewer 3

#3. you have incorporated my comments into your manuscript very carefully. I recommend your paper for publication without further revision.

[Response]
We thank the reviewer for the positive evaluation and recommendation for publication. We greatly appreciate the constructive feedback provided throughout the review process.

Comments from Reviewer 4

#4.1. In the Introduction, citing several recently published studies would help better situate the findings within the current body of literature and clarify the manuscript's contribution relative to prior work.

This is particularly important given the broad readership of the journal. Below are examples of relevant recent publications that could be considered.

a. Liang, C., Yuan, J.*, Zhang, R. et al. Projecting the morbidity burden of mental and behavioral disorders associated with increasing humid heat in Shanghai. Nat. Mental Health (2025). https://doi.org/10.1038/s44220-025-00519-y

b. Min, H. et al. Ambient temperature, suicide, and urbanicity: A nationwide time-stratified case-crossover study in South Korea. PLOS ONE 20, e0337945 (2025).

c. Baecker L, et al. (2025). Impacts of extreme heat on mental health: Systematic review and qualitative investigation of the underpinning mechanisms. Journal of Climate Change and Health, 22: 100446. https://doi.org/10.1016/j.joclim.2025.100446

[Response]

We thank the reviewer again for this constructive recommendation. We agree that incorporating recent publications strengthens the contextualization of our study and clarifies its contribution to the rapidly evolving literature on climate change and mental health. Accordingly, we have revised the Introduction to incorporate the recommended studies as follows:

a. Liang et al. (2025) has been added to support the statement "Projections of climate-sensitive health responses are essential for anticipating future public health challenges due to climate change and informing policy. Several studies have predicted the impacts of climate change on human health including morbidity and mortality across various countries[21–29]." (Lines 152-154, page 6, in main text).

b. Min et al. (2025) has been incorporated as supporting evidence for the statement: "Numerous studies have identified a positive association between ambient temperature and suicide, suggesting that higher temperatures increase suicide risk[17–20]." (Lines 141-142, page 6, in main text)

c. Baecker et al. (2025) has been incorporated as supporting evidence for the statement: "High temperatures can exacerbate poor mental health outcomes through insomnia, increased stress levels, and psychological instability, all of which contribute to suicide risk[3–5,14–16]." (Lines 139-141, page 6, in main text)

(Lines 153-154, pages 6, in main text)

Several studies have predicted the impacts of climate change on human health including morbidity and mortality across diverse countries[21-29].

(Lines 141-142, page 6, in main text)

Numerous studies have identified a positive association between ambient temperature and suicide, suggesting that higher temperatures increase suicide risk[17–20].

(Lines 139-141, page 6, in main text)

High temperatures can exacerbate poor mental health outcomes through insomnia, increased stress levels, and psychological instability, all of which contribute to suicide risk[3–5,14–16].

Reference

16. Baecker L, et al. Impacts of extreme heat on mental health: Systematic review and qualitative investigation of the underpinning mechanisms. J. Clim. Chang. Health. 22, 100446 (2025).

19. Min, H. et al. Ambient temperature, suicide, and urbanicity: A nationwide time-stratified case-crossover study in South Korea. PLoS One. 20(12), e0337945 (2025).
24. Liang, C. et al. Projecting the morbidity burden of mental and behavioral disorders associated with increasing humid heat in Shanghai. Nat. Mental Health 3(12), 1532–1544 (2025).

#4.1.2 Some additional limitations should be discussed. First, the analysis focuses on daily mean temperature and does not explicitly account for the potentially distinct effects of extreme heat or cold events. Second, suicide count data rather than rate-based data were used, as information on population size and temporal changes in suicide rates was not available.

[Response]
We agree that these limitations are important considerations to be discussed. We added a paragraph (Lines 399-406, pages 16, in main text) and additional text (Lines 417-423, page 16, in main text) to discuss the limitations of using daily mean temperature as an exposure metric and suicide count data rather than rate-based data as health outcomes, respectively.

(Lines 399-406, pages 16, in main text)
A further limitation arises from representing thermal exposure using only daily mean temperature. Mean temperature smooths intra-day variability and may fail to capture extreme daytime heat or elevated nighttime temperatures, both of which may influence psychological stress, sleep quality, and behavioral responses associated with suicide risk. Because climate change may differentially affect daytime and nighttime temperatures and increase the frequency and persistence of heat extremes, projections based solely on daily mean temperature may underestimate or mischaracterize the future burden of temperature-related suicide. Future studies may benefit from incorporating additional exposure metrics such as daily maximum and minimum temperature, heatwave indicators, or composite thermal indices.

(Lines 417-423, page 16, in main text)
In addition, the projections were based on suicide count data rather than suicide rates. Because counts are not standardized by the population at risk, this approach may limit comparability across regions with different population sizes and may obscure the influence of demographic changes such as population growth or aging. Future studies may benefit from incorporating population-standardized suicide rates and demographic projections to better disentangle climate-related risk changes from demographic influences.
