# Supplementary material for: Heat-related urban mortality attributable to human-induced climate change in Europe from 2000 to 2025
Source: Nat Health. Author manuscript; Available in PMC 2026 Oct 29. (PMC7619643; doi:10.1038/s44220-026-00674-w)
Supplement: Supplementary file 4 [file EMS221703-supplement-Supplementary_information.pdf]

nature mental health

Supplementary information https://doi.org/10.1038/s44220-026-00674-w

Multi-country projections of temperature-related suicide mortality

In the format provided by the authors and unedited

Supplemental information

Table of contents

1. Details on Data Collection

2. Details on Statistical Analysis

Table S1 | Projected total attributable fraction (AF, %) of suicide mortality associated with temperatures by region and decade under three climate change scenarios. Data are presented as mean values with 95% eCIs derived from n = 1,000 Monte Carlo simulations across n = 5 General Circulation Models.

Table S2 | Projected total attributable number (AN) of suicide deaths associated with temperatures by region and decade under three climate change scenarios. Data are presented as mean values with 95% eCIs derived from n = 1,000 Monte Carlo simulations across n = 5 General Circulation Models.

Table S3 | Country-specific root mean square errors (RMSEs) between modeled and observed temperatures before and after calibration under three climate scenarios.

Table S4 | Model selection for the first-stage analysis: selection of the number and placement of knots for the exposure–response association in the distributed lag non-linear model (DLNM), based on n = 426 locations.

Table S5 | Variable selection for the second-stage multivariate mixed-effects meta-regression model used to pool location-specific exposure–response associations across n = 751 locations. Candidate meta-predictors along with random effects at the country/city level were evaluated and selected based on the Akaike information criterion (AIC).

Table S6 | Summary of selected locations where the minimum projected temperature in the 2050s under the high-emission scenario (SSP5-8.5) and the observed median temperature, used to validate the appropriateness of the 50th percentile as the reference temperature. SSP, shared socioeconomic pathway.

Fig. S1 | Map of the average observed temperatures at the 751 locations included in the analysis. Colours represent the mean daily temperature (°C) during the observation period at each location. Map data © EuroGeographics, sourced via the ‘giscoR 0.6.1’ R package (CC BY 4.0).

Fig. S2 | Decadal changes in mean temperature (°C) relative to the 2010s by region and climate scenario (SSP1-2.6, SSP2-4.5, and SSP5-8.5). The historical period spans from 1 January 1971 to 31 December 2014, representing deviations from the 2010s mean temperature. Projected temperatures from 2015 to 2059 are shown by color lines for each scenario, representing changes from the same 2010s. GCM, general circulation model; SSP, shared socioeconomic pathway.

Fig. S3 | Overall cumulative relationship between temperature and suicide by country. The red line represents the pooled relative risk (RR) of suicide mortality across the temperature distribution, estimated from a two-stage meta-regression pooling location-specific exposure–response associations (n = 751 locations across 26 countries). The measure of center and shaded area display the pooled RR estimates and their corresponding 95% CIs. The dotted vertical line indicates the reference temperature

(50th percentile of the country-specific temperature distribution), at which RR = 1. RR, relative risk; CI, confidence interval.

Fig. S4 | Map of projected changes in total attributable fraction (AF, %) of suicide mortality associated with temperatures between the 2010s (2010–2019) and 2050s (2050–2059) under two climate change scenarios: (A) SSP1-2.6 and (B)SSP2-4.5, across 751 locations in 26 countries. Map data © EuroGeographics, sourced via the 'giscoR 0.6.1' R package (CC BY 4.0). AF, attributable fraction; SSP, shared socioeconomic pathway.

Fig. S5 | Regional relative risks of suicide mortality at temperature extremes, projected warming, and change in total attributable fractions of suicide mortality under two climate change scenarios: (A) SSP1-2.6 and (B) SSP2-4.5. The measure of center and error bars display the pooled RR values at the 1st and 99th percentiles of the regional temperature distribution and their corresponding 95% CIs, derived from a two-stage meta-regression (n = 751 locations across 10 regions and 26 countries). Points in panel (b) represent the mean projected temperature change (°C) averaged across n = 5 GCMs. Bars in panel (c) represent the mean difference in total AF averaged across n = 5 GCMs. RR, relative risk; AF, attributable fraction; GCM, general circulation model; SSP, shared socioeconomic pathway; CI, confidence interval.

Fig. S6 | Temperature distribution in the 2050s (2050–2059) under the high-emission scenario (SSP5-8.5) compared to the observed temperature distribution for each of the 26 selected locations. Distributions represent the ensemble mean across n = 5 GCMs. SSP, shared socioeconomic pathway; GCM, general circulation model.

Fig. S7 | Temporal changes in attributable fraction (AF, %) of suicide mortality associated with warm (above reference temperature) and cold (below reference temperature) temperatures for each decade relative to the 2010s by region under three climate change scenarios (SSP1-2.6, SSP2-4.5, and SSP5-8.5). The measure of center and error bars display the mean values and 95% eCIs derived from n = 1,000 Monte Carlo simulations across n = 5 GCMs. Regional estimates were aggregated from locations within each corresponding region (total n = 751 locations across 10 regions).

Fig. S8 | Map of projected changes in attributable fractions (AF, %) of suicide mortality associated with warm temperatures (above the reference temperature) between the 2010s (2010–2019) and 2050s (2050–2059) under three climate change scenarios: (A) SSP1-2.6, (B) SSP2-4.5, and (C) SSP5-8.5, across n = 751 locations in 26 countries. Map data © EuroGeographics, sourced via the 'giscoR 0.6.1' R package (CC BY 4.0). AF, attributable fraction; SSP, shared socioeconomic pathway.

Fig. S9 | Map of projected changes in attributable fractions (AF, %) of suicide mortality associated with cold temperatures (below the reference temperature) between the 2010s (2010–2019) and 2050s (2050–2059) under three climate change scenarios: (A) SSP1-2.6, (B) SSP2-4.5, and (C) SSP5-8.5, across n = 751 locations in 26 countries. Map data © EuroGeographics, sourced via the 'giscoR 0.6.1' R package (CC BY 4.0). AF, attributable fraction; SSP, shared socioeconomic pathway.

Fig. S10 | Temperature–suicide associations and temporal changes in total attributable fractions (AF, %) using the minimum suicide temperature (MinST) as the reference temperature. The upper panels show the overall cumulative exposure–response curves by region with the MinST as the reference, estimated from a two-stage meta-regression (n = 751 locations across 10 regions). The

measure of center and shaded area display the pooled RR estimates and their corresponding 95% CIs. The lower panels show the decadal changes in total AF relative to the 2010s under three climate change scenarios (SSP1-2.6, SSP2-4.5, and SSP5-8.5). Data are presented as mean values (bars) ± 95% eCIs (error bars) derived from n = 1,000 Monte Carlo simulations across n = 5 GCMs. RR, relative risk; AF, attributable fraction; SSP, shared socioeconomic pathway; CI, confidence interval.

Fig. S11 | Sensitivity analysis comparing temporal changes in total attributable fraction (AF, %) of suicide mortality using short-term lag structures of 3-day and 6-day lags, based on data from n = 426 locations. Data are presented as mean values (bars) ± 95% eCIs (error bars) derived from n = 1,000 Monte Carlo simulations across n = 5 GCMs. Gray circles overlaid on each bar show individual GCM-level estimates (n = 5 per bar). AF, attributable fraction; GCM, general circulation model; eCI, empirical confidence interval.

1. Data Collection Details

The data used in this study were obtained from the MCC (Multi-Country Multi-City) Collaborative Research Network (https://mccstudy.lshtm.ac.uk/) under a data-sharing agreement, and therefore, cannot be publicly shared.

Canada (26 cities, 1986-2015) Data were collected from 26 cities over the period from 1 January 1986 to 31 December 2015. Daily suicide counts were sourced from Statistics Canada, while daily mean temperature (°C) data were obtained from Environment Canada.

United States (134 cities, 2001-2006) Data were collected from 135 cities over the period from 1 January 2001 to 31 December 2006. Honolulu was excluded due to no recorded suicide deaths during this period, resulting in a total of 134 cities included in the analysis. Daily suicide counts were sourced from the National Center for Health Statistics, while daily mean temperature (°C) data were obtained from the National Climate Data Center (NCDC) of the National Oceanic and Atmospheric Administration (NOAA).

Costa Rica (1 city, 2000-2017) Data were collected from 1 city (San Jose) over the period from 1 January 2000 to 31 December 2017. Daily suicide counts were sourced from the Instituto Nacional de Estadística y Censo [Open Access], while daily mean temperature (°C) data were obtained from WMO-NOAA (Surface Data Hourly Global, DS3505).

Guatemala (1 city, 2009-2018) Data were collected from 1 city (Guatemala) over the period from 1 January 2009 to 31 December 2018. Daily suicide counts were sourced from the Instituto Nacional de Estadística, Unidad de Estadística de Salud, while daily mean temperature (°C) data were obtained from the Instituto Nacional de Sismología, Vulancología, Meteorología y Hidrología.

Mexico (10 cities, 1998-2014) Data were collected from 10 cities over the period from 1 January 1998 to 31 December 2014. Daily suicide counts were sourced from the National Institute of Statistics, Geography and Informatics, while daily mean temperature (°C) data were obtained from the Servicio Meteorológico Nacional (SMN) and the Instituto Nacional de Ecología y Cambio Climático (INECC).

Brazil (15 cities, 2000-2021) Data were collected from 15 cities from 1 January 2000 to 31 December 2021. Daily suicide counts were sourced from the Ministry of Health, Brazil, while daily mean temperature (°C) data were obtained from the National Institute of Meteorology of Brazil.

Chile (1 city, 2008-2014) Data were collected from 1 city (Santiago) from 1 January 2008 to 31 December 2014. Daily suicide counts were sourced from Departamento de Estadísticas e Información en Salud, while daily mean temperature (°C) data were obtained from Sistema Informático de Calidad del Aire.

Ecuador (2 cities, 2013-2019) Data were collected from 2 cities (Guayaquil and Quito) from 1 January 2013 to 31 December 2019. Daily suicide counts were sourced from Instituto Nacional de Estadística y Censo [Open Access], while daily mean temperature (°C) data were obtained from from WMO-NOAA (Surface Data Hourly Global, DS3505).

Paraguay (1 city, 2004-2019) Data were collected from 1 city (Asuncion) from 1 January 2004 to 31 December 2019. Daily suicide counts were sourced from the Ministerio de Salud Pública y Bienestar

Social, Dirección General de Información Estratégica en Salud, Subsistema de Información de Estadísticas Vitales, while daily mean temperature (°C) data were obtained from the Global Historical Climatology Network (NOAA/WMO).

Estonia (5 cities, 2005-2015) Data were collected from 5 cities from 1 January 2005 to 31 December 2015. Daily suicide counts were sourced from the Estonian Causes of Death Registry, while daily mean temperature (°C) data were calculated as the 24-hour average of hourly measurements obtained from Estonian Environment Agency.

Finland (307 municipalities, 1971-2018) Data were collected from 310 municipalities (Kunta) from 1 January 1971 to 31 December 2018. Sotunga was excluded due to no recorded suicide deaths during this period, and Kökar and Vårdö were excluded as each had only one case, resulting in a total of 307 municipalities included in the analysis. Daily suicide counts were sourced from Statistics Finland, while daily mean temperature (°C) data was obtained from the Finnish Meteorological Institute (FMI).

United Kingdom (10 regions, 2001-2014) Data were collected from 10 regions in England and Wales from 1 January 2001 to 31 December 2014. Daily suicide counts were sourced from the Office of National Statistics, while daily mean temperature (°C) data were obtained from the British Atmospheric Data Centre.

Czech Republic (3 cities, 2003-2019) Data were collected from 3 cities from 1 January 2003 to 31 December 2019. Daily suicide counts were sourced from the Czech Statistical Office and the Institute of Health Information and Statistics, while daily mean temperature (°C) data were obtained from the Czech Hydrometeorological Institute.

Germany (15 cities, 2000-2020). Data were collected from the 15 largest cities from 1 January 2000 to 31 December 2020. The raw data set of daily suicide counts was accessed via a workstation for visiting scientists at the Research Data Centre (RDC) of the Federal Statistical Office and Statistical Offices of the Federal States ([Mortality Statistics (EVAS 23211)], survey years [2000-2020], DOI: 10.21242/23211.2000.00.00.1.1.0 to 10.21242/23211.2020.00.00.1.1.0, own calculations), while daily mean temperature (°C) data were obtained from the German National Meteorological Service (Deutscher Wetterdienst, DWD).

Romania (8 cities, 1999-2016) Data were collected from 8 cities from 1 January 1999 to 31 December 2016. Daily suicide counts were sourced from the National Institute for Statistics (NIS), while daily mean temperature (°C) data were obtained from Ciampino airport were obtained from Romanian National Meteorological Administration (RNMA).

Switzerland (8 cantons, 1978-2016) Data were collected from 8 regions (cantons) from 1 January 1978 to 31 December 2016. Daily suicide counts were sourced from the Federal Office of Statistics, while daily mean temperature (°C) data were obtained from the IDAWEB web database (a service provided by MeteoSwiss, the Swiss Federal Office of Meteorology and Climatology, MeteoSwiss). A single weather station within each canton was selected (Basel-Stadt: Basel/Binningen; Berne: Berne/Zollikofen; Geneva: Genève/Cointrin; Ticino: Lugano; Lucerne: Lucerne; Vaud: Pully; St. Gallen: St. Gallen; Zurich: Zurich/Fluntern).

Italy (1 city, 2006-2015) Data were collected from 1 city (Rome) from 1 January 2006 to 31 December 2015. Daily suicide counts were sourced from Lazio regional mortality registry (ReNCaM), while daily mean temperature (°C) data were obtained from Ciampino airport were obtained from the Italian Air Force Meteorological and Climatological Service (CNMCA).

Spain (50 cities, 1990-2013) Data were collected from 52 provincial capital cities from 1 January 1990 to 31 December 2013. Palencia and Ceuta were excluded due to a lack of temperature data since 1991 and before 2003, respectively, resulting in a total of 50 provincial capital cities included in the analysis. Daily suicide counts were sourced from Spain National Institute of Statistics, while daily mean temperature (°C) data were obtained from Ciampino airport were obtained from Spain National Meteorology Agency.

China (1 city, 2015-2019) Data were collected from 1 city (Suzhou) from 1 January 2015 to 31 December 2019. Daily suicide counts were sourced from Suzhou Center for Disease Control and Prevention, while daily mean temperature (°C) data were obtained from Suzhou Bureau of Meteorology.

Japan (47 prefectures, 1973-2020) Data were collected from 47 prefectures from 1 January 1973 to 31 December 2019. Daily suicide counts were sourced from the Ministry of Health, Labour and Welfare, Japan, while daily mean temperature (°C) data were obtained from the Japan Meteorological Agency

South Korea (36 cities, 1997-2021) Data were collected from 36 cities from 1 January 1997 to 31 December 2019. Daily suicide counts were sourced from the Microdata Integrated Service (MDIS), administered by Statistics Korea in South Korea, while daily mean temperature (°C) data were obtained from the Korea Meteorological Administration.

Philippines (8 cities, 2006-2019) Data were collected from 8 cities from 1 January 2006 to 31 December 2019. Daily suicide counts were sourced from the Philippine Statistics Agency, while daily mean temperature (°C) data were obtained from the Philippine Atmospheric, Geophysical and Astronomical Services Administration.

Taiwan (3 cities, 1994-2014) Data were collected from 3 cities from 1 January 1994 to 31 December 2014. Daily suicide counts were sourced from the Department of Statistics, Ministry of Health and Welfare in Taiwan, while daily mean temperature (°C) data were obtained from the Taiwan Environmental Protection Administration.

Vietnam (1 city, 2010-2013) Data were collected from 1 city (Ho Chi Minh) from 1 January 2010 to 31 December 2013. Daily suicide counts were sourced from national mortality system collected at community namely A6, while daily mean temperature (°C) data were obtained from the Tan Son Nhat airport weather station, Ho Chi Minh City.

Australia (5 cities, 2009-2019) Data were collected from 5 largest cities in Australia (Melbourne, Sydney, Brisbane, Perth, Adelaide) from 1 January 2009 to 31 December 2019. Daily suicide counts were sourced from the Australian Bureau of Statistics, while daily mean temperature (°C) data were obtained from the Australian Bureau of Meteorology.

South Africa (52 districts, 1997-2013) Data were collected from 52 districts from 1 January 1997 to 31 December 2013. Daily suicide counts were sourced from Statistics South Africa, but they played no role in data analysis or interpretation, and there is a high possibility of underreporting of suicide data.

Daily mean temperature (°C) data were obtained from the Agricultural Research Council of South Africa and the National Oceanographic and Atmospheric Association (NOAA) of the United States.

2. Details on Statistical Analysis

2.1 Estimation of the location-specific temperature–suicide association

2.1.1 First stage modeling

To estimate location-specific temperature-suicide association, a conditional Poisson regression model[1], taking overdispersion into account, was applied within a time-stratified case-crossover design[2] to control for temporal confounding factors such as long-term trends, seasonal patterns, and day-of-week effects. Strata were defined based on the three-way interaction of calendar year, month, and day of the week, so that comparisons were made between case days and their matched control days within the same stratum. To capture the nonlinear and delayed effects of temperature on suicide, a distributed lag non-linear model (DLNM)[3] was employed with a bidimensional cross-basis spline function with a maximum lag of 3 days. The conditional Poisson regression was formulated as the following multinomial model[1]:

$$\begin{cases} \left(D_{i,1,s}^{obs}, D_{i,2,s}^{obs}, \dots D_{i,t_s,s}^{obs}\right) \mid D_{i,\cdot,s}^{obs} \sim Multinomial\left(D_{i,\cdot,s}^{obs}, \pi_{i,1,s}, \pi_{i,2,s}, \dots, \pi_{i,t_s,s}\right) \\ D_{i,\cdot,s}^{obs} := \sum_{t=1}^{t_s} D_{i,t,s}^{obs} \\ \pi_{i,t,s} = exp\left(f\left(T_{i,t}^{obs}; \eta_i\right)\right) \;/\; \sum_{t \in s(t)} exp\left(f\left(T_{i,t}^{obs}; \eta_i\right)\right) \end{cases}$$

The observed daily suicide counts $D_{i,t,s}^{obs}$, in location i on day t within each stratum s, follow a multinomial distribution with probability parameters $\pi_{i,t,s}$. These probabilities depend on the bidimensional cross-basis spline function $f(\cdot)$ where $T_{i,t}^{obs}$ is the observed temperature and η_i is the corresponding basis coefficients. The exposure-response association was modeled using a natural cubic spline with a single internal knot positioned at the 50th percentile of the observed temperature distribution for each location.[8] The lag-response association was parameterized using dummy variables to define intervals for lag 0 and lags 1–3, capturing the short-term delayed effects of temperature on suicide mortality.[8] The number of knots and its locations were determined based on quasi Akaike's Information Criterion (QAIC) values in Table S4. As previous projection studies commonly used a 3-day lag, we compared 3- and 6-day lags to determine an appropriate short-term lag. The QAIC values and sensitivity analysis were calculated by fitting the data that excluded four locations in Australia, 14 locations in Germany, and the entire dataset from Finland, which were analyzed remotely.

To facilitate interpretation of the cumulative effect over time, the bidimensional spline function was transformed into a unidimensional one by reducing the lag dimension to represent overall cumulative exposure-response curve.[9] The location-specific overall cumulative exposure-response associations were expressed as relative risk (RR) curves, representing the ratio of the suicide deaths associated with observed range of temperature exposures compared to a reference temperature. The reduced coefficients $\hat{\theta}_i \in \mathbb{R}^{v_x}$ are obtained along with their corresponding variance–covariance matrices S_i, defined as follows[9]:

$$\hat{\theta}_i = \mathrm{M}_{[c]}\hat{\eta}_i, \qquad \mathrm{S}_i = \mathrm{M}_{[c]}\mathrm{Var}(\hat{\eta}_i)\mathrm{M}_{[c]}^{\mathrm{T}}$$

where $\hat{\eta}_i$ is the vector of estimated coefficients from the bidimensional cross-basis spline function, with v_x basis functions for temperature and v_l for lag. The reducing matrix $\mathrm{M}_{[c]} \in \mathbb{R}^{v_x \times (v_x v_l)}$ collapses the lag dimension by summing the coefficients associated with each temperature

basis function across all lag basis functions. Specifically, $\mathrm{M}_{[c]} = 1_{v_l}^{\mathrm{T}}\mathrm{C} \otimes \mathrm{I}_{v_x}$, where C is the matrix of lag basis functions and I_{v_x} is the identity matrix of size v_x. This transformation results in a unidimensional summary of the association $\hat{\theta}_i$ that represents the overall cumulative effect of temperature on suicide mortality across the lag period.

2.1.2 Second stage modeling

The location-specific overall cumulative exposure-response associations $\hat{\theta}_i$ were then pooled using a multivariate mixed-effects meta-regression model.[6,9,10] Random effects were incorporated to account for intercept variability at both country and city levels, assuming a hierarchical structure where cities are nested within countries. Meta-predictors were included as fixed effects, such as region, country-level GDP, and location-specific variables (e.g., average temperature and the range of daily mean temperatures during the observed period). To identify the best-fitting model, various combinations of four fixed-effect variables and random effects at the region/country and country/city levels were evaluated. The model with the lowest I^2 statistic (6.7%) was selected, indicating minimal heterogeneity in the meta-analysis (see Table S5).[11] The meta-regression model is specified as[6,9,10]:

$$\hat{\theta}_i = X_i\beta + Z_i b_i + \epsilon_i$$

where $X_i\beta$ represents the fixed-effect terms, with X_i being the design matrix of meta-predictors and β the corresponding coefficient vector. The term $Z_i b_i$ represents the random intercepts for cities nested within countries, and ϵ_i denotes the residual error capturing the uncertainty from the first-stage estimates. Both b_i and ϵ_i follow zero-mean multivariate normal distributions with variance–covariance matrices Ψ_i and S_i, respectively.

Based on this meta-regression, Best Linear Unbiased Predictions (BLUPs) $\hat{\theta}_i^b$ were estimated for each location to quantify the association between temperature and suicide mortality. These location-specific predictions serve as the adjusted coefficients to represent the temperature-suicide association for projecting the impact of climate change on suicide risk. The BLUPs formula is given by[6]:

$$\hat{\theta}_i^b = X_i\hat{\beta} + Z_i\hat{\Phi}_i Z_i^T \Sigma_i^{-1}(\hat{\theta}_i - X_i\hat{\beta})$$

where the marginal variance of $\hat{\theta}_i$ is defined as $\Sigma_i = \mathrm{S}_i + Z_i\Psi_i Z_i^T$. This modeling approach allows for stable estimation by borrowing information from data-rich locations to support locations with lower observed suicide counts but similar climatic and socioeconomic conditions.[10]

2.2 Projection of the region-specific impact of climate change on suicide mortality

2.2.1 Calculation of future temperature-related suicide mortality

To estimate the impact of climate change on suicide, future temperature-related suicide mortality was calculated for each region under the assumption of no adaptation (i.e., no changes in the exposure-response relationship over time), no demographic changes (i.e., a static population size and age distribution), and no change in suicide rate. The location-specific temperature–suicide associations were extrapolated up to 2059 using the modeled temperature data and then linked with the modeled suicide data to project future temperature-related suicide mortality.

First, we selected a reference temperature to calculate the suicide mortality associated with temperatures. The reference temperature serves as the baseline for suicide risk across all temperatures, representing a

state with no additional temperature-related suicide risk. The attributable number or fraction of suicide mortality at other temperatures is calculated relative to this baseline. Setting the reference temperature within a range likely to exist in the future, we can compare suicide risk at future temperatures with a meaningful and stable baseline, ensuring robust assessment and maintaining calculation reliability. For each location, we defined the reference temperature T_i^{ref} as the 50th percentile of its observed temperature distribution. This choice is based on the evidence that the median of the current temperature distribution is expected to remain within the projected 2050s temperature range even under the most extreme scenario, SSP5-8.5 among all locations (see Table S3, Figure S2). Specifically, for each country, we identified the warmest location based on the projected minimum temperature in the 2050s under SSP5-8.5 and examined where this temperature falls within the observed temperature distribution. Among the selected locations, the projected minimum temperature in Fortaleza, Brazil—the location with the highest percentile of the observed temperature distribution—closely matches the 50th percentile of observed temperatures. In other words, under SSP5-8.5 in the 2050s, the minimum temperature is projected to reach 27.6 °C, which is nearly equal to the observed median (Table S6, Fig. S9).

Using these reference temperatures, the attributable number (AN) and attributable fraction (AF) of suicide mortality were then calculated for each location and for each combination of the three Shared Socioeconomic Pathways (SSPs) and five General Circulation Models (GCMs). [4,5] The AF and AN were computed using the following formulas:

$$\mathrm{AF}_{T,t} = 1 - e^{\left(-f(T_t;\eta)\right)}, \quad \mathrm{AN}_{T,t} = \mathrm{AF}_{T,t} \cdot D_t$$

where D_t represents the number of suicide deaths at time t. These values indicate the number ($\mathrm{AN}_{T,t}$) and fraction ($\mathrm{AF}_{T,t}$) of suicide deaths at time t attributable to temperatures during the lag period, relative to the reference temperature. Conceptually, the AF represents the proportion of suicide burden at a given temperature that is attributable to deviations from the reference temperature, while the AN quantifies the number of suicide deaths due to temperature changes compared with the total number of suicides at that temperature, reflecting how many deaths are estimated to occur (or be prevented) due to temperatures. For instance**,** a positive AF (RR > 1) indicates an increased suicide burden at higher-than-reference temperatures, whereas a negative AF (RR < 1) reflects a relative reduction or protective effect at lower-than-reference temperatures.

Next, we calculated the daily number of attributable suicide deaths $D_{i,t}^{attr}$ for each of the days with modeled temperatures $T_{i,t}^{mod}$ as follows:

$$D_{i,t}^{attr} = D_{i,t}^{mod}\left[1 - e^{-\left(f\left(T_{i,t}^{mod};\hat{\theta}_i^b\right) - f\left(T_i^{ref};\hat{\theta}_i^b\right)\right)}\right]$$

where $D_{i,t}^{mod}$ is modeled suicide deaths and $\hat{\theta}_i^b$ denotes the reduced coefficients from the second stage modeling. As the temperature-suicide association is monotonically increasing, $D_{i,t}^{attr}$ is positive/negative for the days when the modeled temperature $T_{i,t}^{mod}$ is higher/lower than the reference temperature T_i^{ref}. Subsequently, for each location, we aggregated $D_{i,t}^{attr}$ across all days within each decade to calculate location-specific decadal attributable numbers (ANs). These ANs were then pooled

by region and across SSPs. Ensemble averages across GCMs were calculated for the pooled ANs to obtain the projected region-specific decal ANs under each climate scenario.

Finally, total attributable fractions (AFs) are calculated as the ratio of the GCMs-ensemble ANs to the total modeled suicide deaths within the corresponding period in each region.[4-7,12] This fraction provides a measure of temperature-related suicide mortality under climate change.

2.2.2 Quantification of uncertainty.

Monte Carlo simulations at the meta-regression model level were conducted to assess the uncertainty in temperature-related suicide mortality, accounting for the dependency among location-specific predictions due to the shared fixed-effect component in the predictive meta-regression model, as well as the uncertainty in the spatial distribution of random effects.[6] In addition to the uncertainty from the estimated exposure–response functions, uncertainty from climate projections was propagated by incorporating temperature projections from five GCMs under three SSP scenarios. The variability across these climate models and scenarios was integrated into the Monte Carlo simulations, allowing the combined uncertainty from both exposure–response estimation and climate projections to be reflected in the final impact estimates.

A total of 1,000 samples of the estimated meta-regression coefficients were drawn from a multivariate normal distribution, $\beta \sim \mathrm{MN}(\hat{\beta}, V_{\beta})$, to generate fixed-effect prediction for each location $\hat{\theta}_i^f = X_i\hat{\beta}$. To incorporate uncertainty related to the spatialisation of random effects, BLUP residuals were computed as $\hat{\xi}_i = \hat{\theta}_i^b - \hat{\theta}_i^f = Z_i\hat{\Phi}_i Z_i^T \Sigma_i^{-1}(\hat{\theta}_i - X_i\hat{\beta})$ and also simulated from a normal distribution, $\xi_i \sim \mathrm{N}\left(\hat{\xi}_i, V(\hat{\xi}_i)\right)$, under the assumption of independence between fixed and random effects within the meta-analysis framework.[6] Using these samples, location-specific synthetic predictions were computed as $\hat{\theta}_i^b = \hat{\theta}_i^f + \hat{\xi}_i$, which were then used to calculate impact measures and 95% empirical confidence intervals (eCIs). Specifically, aggregation was conducted separately for each Monte Carlo iteration. The impact measures were obtained as point estimates by averaging the aggregated results across Monte Carlo iterations, while the eCIs were derived as the 2.5th and 97.5th percentiles of their empirical distribution, thereby preserving the correlation structure across locations and allowed for robust estimation and projection of temperature-related suicide mortality.

Table S1 | Projected total attributable fraction (AF, %) of suicide mortality associated with temperatures by region and decade under three climate change scenarios. Data are presented as mean values with 95% eCIs derived from n = 1,000 Monte Carlo simulations across n = 5 General Circulation Models.

**Region**	**SSP**	**SSP1-2.6**			**SSP2-4.5**			**SSP5-8.5**		
	**(%)**	**2010s**	**2030s**	**2050s**	**2010s**	**2030s**	**2050s**	**2010s**	**2030s**	**2050s**
North America	Total AF	-0.6 (-2.7, 1.4)	0.1 (-2.2, 2.4)	0.4 (-2.1, 2.9)	-0.6 (-2.8, 1.3)	0.2 (-2.1, 2.4)	0.7 (-1.8, 3.1)	-0.7 (-2.9, 1.3)	0.2 (-2.2, 2.4)	1.3 (-1.6, 4.4)
	Difference from 2010s	-	0.6 (0.2, 1.6)	1.0 (0.3, 2.3)	-	0.8 (0.3, 1.6)	1.3 (0.5, 2.6)	-	0.9 (0.3, 1.8)	2.0 (0.8, 4.1)
Central America	Total AF	0.6 (-1.7, 2.7)	2.8 (-0.3, 6.1)	3.8 (0.6, 7.2)	0.8 (-1.3, 3.0)	3.0 (0.4, 5.7)	5.2 (2.0, 8.9)	0.7 (-1.5, 2.6)	3.3 (0.5, 6.1)	7.2 (3.6, 12.2)
	Difference from 2010s	-	2.1 (1.0, 3.9)	3.2 (1.8, 5.3)	-	2.2 (1.0, 3.8)	4.4 (2.4, 6.7)	-	2.6 (1.3, 4.4)	6.4 (4.0, 10.6)
South America	Total AF	1.0 (-1.0, 2.9)	2.4 (0.1, 4.5)	3.5 (0.7, 6.3)	1.0 (-0.9, 2.7)	2.6 (0.4, 4.7)	4.3 (1.5, 7.5)	1.0 (-0.9, 2.7)	2.8 (0.6, 4.9)	5.6 (2.5, 9.2)
	Difference from 2010s	-	1.4 (0.6, 2.5)	2.4 (1.1, 4.8)	-	1.6 (0.6, 3.2)	3.4 (1.6, 6.2)	-	1.9 (0.8, 3.3)	4.7 (2.5, 7.8)
North Europe	Total AF	0.7 (-1.1, 2.3)	0.8 (-1.3, 2.8)	1.3 (-0.8, 3.3)	0.6 (-1.4, 2.4)	1.2 (-0.9, 3.1)	1.5 (-0.8, 3.7)	0.5 (-1.4, 2.2)	1.2 (-0.8, 3.0)	2.0 (-0.3, 4.7)
	Difference from 2010s	-	0.2 (-0.4, 0.9)	0.6 (0.1, 1.7)	-	0.6 (0.2, 1.5)	0.9 (0.3, 2.2)	-	0.6 (0.2, 1.3)	1.5 (0.4, 3.3)
Central Europe	Total AF	0.3 (-1.7, 2.0)	0.5 (-1.9, 2.6)	1.0 (-1.2, 3.1)	0.2 (-2.0, 2.0)	0.9 (-1.4, 2.9)	1.3 (-1.1, 3.6)	0.3 (-1.8, 2.0)	0.8 (-1.3, 2.8)	1.8 (-0.7, 4.4)
	Difference from 2010s	-	0.2 (-0.2, 1.0)	0.7 (0.2, 1.7)	-	0.7 (0.1, 1.4)	1.1 (0.4, 2.4)	-	0.6 (0.1, 1.2)	1.6 (0.6, 3.3)
South Europe	Total AF	2.0 (-1.3, 5.1)	3.1 (-0.4, 6.4)	3.6 (0.0, 7.1)	2.0 (-1.3, 5.1)	3.1 (-0.4, 6.3)	4.0 (0.3, 7.6)	2.0 (-1.0, 4.9)	3.3 (0.0, 6.5)	5.0 (1.1, 9.1)
	Difference from 2010s	-	1.1 (0.4, 2.1)	1.6 (0.5, 3.3)	-	1.0 (0.3, 2.1)	2.0 (0.9, 4.0)	-	1.3 (0.4, 2.6)	3.0 (1.5, 6.0)
East Asia	Total AF	-4.0 (-7.2, -1.3)	-3.4 (-6.8, -0.6)	-3.1 (-6.5, -0.2)	-4.0 (-7.2, -1.3)	-3.3 (-6.6, -0.5)	-2.8 (-6.2, 0.0)	-4.1 (-7.3, -1.3)	-3.2 (-6.5, -0.4)	-2.0 (-5.3, 0.9)
	Difference from 2010s	-	0.6 (0.0, 1.5)	0.9 (0.2, 1.7)	-	0.7 (0.2, 1.5)	1.2 (0.4, 2.2)	-	0.8 (0.3, 1.9)	2.0 (1.2, 3.5)
South-East Asia	Total AF	-2.1 (-5.2, 0.8)	-0.3 (-3.7, 3.2)	0.4 (-3.2, 4.0)	-2.0 (-5.1, 0.8)	-0.2 (-3.4, 2.6)	1.1 (-2.5, 4.9)	-2.1 (-5.2, 0.8)	0.0 (-3.4, 3.4)	2.8 (-1.1, 8.0)
	Difference from 2010s	-	1.8 (0.6, 3.5)	2.5 (1.0, 4.6)	-	1.8 (0.7, 3.8)	3.1 (1.3, 6.0)	-	2.2 (0.7, 4.1)	5.0 (2.5, 9.2)

Australia	Total AF	-0.8 (-4.4, 2.1)	-0.2 (-3.6, 2.5)	-0.1 (-3.6, 2.7)	-0.8 (-4.2, 2.1)	-0.2 (-3.6, 2.6)	0.4 (-3.1, 3.3)	-0.7 (-4.0, 2.0)	-0.1 (-3.2, 2.4)	0.9 (-2.4, 3.8)
	Difference from 2010s	-	0.6 (-0.1, 1.4)	0.7 (-0.2, 2.0)	-	0.6 (-0.1, 1.7)	1.2 (-0.3, 3.2)	-	0.6 (-0.2, 2.0)	1.7 (-0.4, 4.4)
South Africa	Total AF	-0.2 (-5.6, 4.3)	1.5 (-3.6, 5.9)	2.1 (-3.5, 7.0)	-0.3 (-5.6, 4.2)	1.5 (-3.9, 6.1)	3.4 (-2.4, 8.4)	-0.3 (-5.5, 4.0)	1.6 (-3.4, 5.9)	4.5 (-1.3, 10.1)
	Difference from 2010s	-	1.7 (0.5, 3.3)	2.3 (0.7, 5.3)	-	1.8 (0.6, 3.8)	3.6 (1.4, 6.5)	-	1.9 (0.7, 3.5)	4.8 (1.9, 8.5)

The total attributable fraction (AF) refers to the proportion of suicide mortality associated with other than the reference temperature. The estimates represent decadal averages derived from the GCMs-ensemble. Differences in total AF reflects the temporal change in total AF relative to the 2010s baseline. Values in parentheses indicate the 95% eCIs. GCM, general circulation model; SSP, shared socioeconomic pathway. eCIs, empirical confidence intervals

Total AF, total attributable fractions; Difference from the 2010s, difference in total attributable fraction compared to the 2010s

Table S2 | Projected total attributable number (AN) of suicide deaths associated with temperatures by region and decade under three climate change scenarios. Data are presented as mean values with 95% eCIs derived from n = 1,000 Monte Carlo simulations across n = 5 General Circulation Models.

Region	SSP	SSP1-2.6			SSP2-4.5			SSP5-8.5		
	(%)	2010s	2030s	2050s	2010s	2030s	2050s	2010s	2030s	2050s
North America	Total AN	-895 (-4271, 2152)	130 (-2490, 3727)	628 (-3319, 4548)	-988 (-4358, 2098)	260 (-3359, 3728)	1110 (-2912, 4962)	-1086 (-4524, 1982)	271 (-3549, 3875)	2073 (-2482, 6925)
	Difference from 2010s	-	1025 (255, 2458)	1523 (418, 3582)	-	1248 (437, 2595)	2098 (811, 4092)	-	1356 (472, 2872)	3159 (1258, 6508)
Central America	Total AN	99 (-261, 425)	431.8 (-40, 946)	600 (100, 1130)	128 (-196, 466)	467 (58, 895)	815 (312, 1398)	114 (-237, 404)	516 (75, 947)	1118 (562, 1901)
	Difference from 2010s	-	332 (162, 614)	501 (276, 824)	-	339 (152, 599)	687 (381, 1049)	-	402 (199, 691)	1004 (620, 1657)
South America	Total AN	258 (-261, 727)	614 (25, 1145)	880 (191, 1616)	252 (-239, 694)	662 (104, 1202)	1106 (381, 1920)	243 (-229, 676)	726 (165.6, 1247)	1428 (625, 2339)
	Difference from 2010s	-	356 (149, 650)	622 (272, 1223)	-	410 (152, 826)	854 (396, 1569)	-	483 (199, 844)	1185 (627, 1975)
North Europe	Total AN	314 (-543, 1092)	397 (-640, 1316)	604 (-385, 1565)	269 (-671, 1126)	549 (-438, 1478)	703 (-360, 1767)	261 (-656, 1062)	550 (-400, 1448)	962 (-141, 2223)
	Difference from 2010s	-	84 (-186, 431)	290 (58, 828)	-	280 (76, 692)	434 (146, 1067)	-	288 (87, 642)	700 (203, 1572)
Central Europe	Total AN	100 (-527, 632)	168 (-573, 809)	307 (-378, 958)	61 (-609, 624)	273 (-425, 892)	397 (-326, 1118)	81 (-544, 614)	256 (-392, 863)	570 (-224, 1371)
	Difference from 2010s	-	68 (-69, 301)	207 (67, 521)	-	211 (38, 440)	336 (122, 745)	-	175 (40, 380)	489 (179, 1019)
South Europe	Total AN	218 (-135, 547)	333 (-47, 687)	389 (3, 760)	220 (-135, 553)	330 (-43, 674)	433 (27, 823)	218 (-111, 528)	357 (1, 699)	537 (115, 976)
	Difference from 2010s	-	115 (42, 224)	171 (52, 353)	-	110 (30, 227)	213 (100, 429)	-	139 (44, 284)	319 (156, 645)
East Asia	Total AN	-12456 (-22334, -3925)	-10681 (-21111, -1749)	-9592 (-20224, -544)	-12490 (-22300, -3975)	-10184 (-20315, -1623)	-8704 (-19333, 111)	-12567 (-22583, -3966)	-10055 (-20227, -1263)	-6241 (-16317, 2801)
	Difference from 2010s	-	1775 (-25, 4548)	2864 (736, 5417)	-	2305 (524, 4685)	3786 (1197, 6748)	-	2512 (776, 5814)	6326 (3572, 10907)
	Total ANs	-299 (-741, 115)	-37 (-528, 449)	62 (-448, 571)	-280 (-718, 117)	-25 (-487, 430)	159 (-357, 694)	-304 (-740, 107)	6 (-476, 489)	401 (-161, 1138)

South-East Asia	Difference from 2010s	-	263 (85, 504)	361 (144, 651)	-	255 (102, 540)	440 (190, 849)	-	309 (93, 585)	704 (358, 1305)
Australia	Total AN	-113 (-596, 291)	-34 (-488, 345)	-10 (-488, 375)	-108 (-577, 290)	-27 (-492, 356)	57 (-419, 448)	-99 (-544, 271)	-10 (-439, 328)	128 (-328, 517)
	Difference from 2010s	-	79 (-18, 193)	102 (-23, 274)	-	81 (-18, 237)	164 (-36, 442)	-	89 (-22, 270)	226 (-51, 603)
South Africa	Total AN	-8 (-200, 155)	55 (-128, 212)	76 (-125, 252)	-10 (-201, 149)	55 (-142, 219)	121 (-85, 301)	-11 (-197, 145)	58 (-122, 211)	161 (-46, 362)
	Difference from 2010s	-	62 (17, 120)	84 (26, 192)	-	64 (21, 136)	130 (50, 233)	-	69 (25, 127)	172 (68, 304)

The total attributable number (AN) refers to the projected number of suicide deaths associated with temperatures other than the reference temperature. The estimates represent decadal averages derived from the GCM-ensemble. Differences in total AN reflect temporal changes relative to the 2010s baseline. Values in parentheses indicate the 95% eCIs. GCM, general circulation model; SSP, shared socioeconomic pathway. eCIs, empirical confidence intervals

Total AN, total attributable number of suicide deaths; Difference from the 2010s, difference in total attributable number compared to the 2010s.

Table S3 | Country-specific root mean square errors (RMSEs) between modeled and observed temperatures before and after calibration under three climate scenarios.

RMSE / Country	SSP1-2.6		SSP2-4.5		SSP5-8.5	
	Before	After	Before	After	Before	After
Canada	4.96	4.91	4.95	4.9	4.95	4.91
United States	4.28	4.13	4.28	4.13	4.28	4.13
Costa Rica	2.14	1.05	2.14	1.05	2.14	1.05
Guatemala	1.40	1.30	1.39	1.29	1.38	1.29
Mexico	2.83	2.4	2.83	2.4	2.83	2.4
Brazil	2.08	1.98	2.09	1.99	2.08	1.99
Chile	3.42	2.79	3.42	2.79	3.42	2.79
Ecuador	2.53	1.17	2.54	1.17	2.55	1.2
Paraguay	4.19	4.18	4.23	4.23	4.19	4.18
Estonia	4.53	4.4	4.53	4.4	4.6	4.46
Finland	5.15	5.13	51.4	5.12	5.16	5.13
United Kingdom	3.00	2.87	3.00	2.87	3.00	2.87
Czech Republic	4.21	4.15	4.26	4.2	4.26	4.21
Germany	4.09	4.08	4.11	4.09	4.13	4.11
Romania	4.21	4.14	4.2	4.15	4.21	4.15
Switzerland	4.47	3.65	4.48	3.65	4.48	3.65
Italy	2.81	2.79	2.8	2.79	2.8	2.79
Spain	3.39	2.96	3.39	2.96	3.39	2.96
China	3.14	3.04	3.16	3.02	3.24	3.07
Japan	3.12	2.64	3.12	2.63	3.13	2.64
Korea	3.38	3.1	3.39	3.11	3.39	3.11
Philippines	1.94	1.25	1.94	1.24	1.92	1.23
Taiwan	3.17	2.29	3.17	2.29	3.17	2.29
Vietnam	1.24	1.15	1.24	1.15	1.24	1.15
Australia	2.96	2.92	2.97	2.94	2.97	2.94
South Africa	3.22	2.95	3.22	2.95	3.22	2.95

Country-level RMSE values for modeled daily mean temperatures before and after calibration under three climate scenarios (SSP1–2.6, SSP2–4.5, and SSP5–8.5). Lower RMSE values after calibration indicate improved agreement between modeled and observed temperatures across countries.

Table S4 | Model selection for the first-stage analysis: selection of the number and placement of knots for the exposure–response association in the distributed lag non-linear model (DLNM), based on n = 426 locations.

Country	natural cubic b-spline (knots) + strata (1 breaks)											
knots	**50**	**75**	**25, 75**	**10, 25, 50**	**10, 25, 75**	**10, 25, 90**	**10, 50, 75**	**10, 50, 90**	**10, 75, 90**	**25, 50, 75**	**25, 50, 90**	**25, 75, 90**
**Canada**	173233	173231	173280	173319	173314	173314	173320	173321	173333	173325	173327	173333
**United States**	272945	272950	273175	273475	273463	273461	273455	273447	273416	273457	273449	273419
**Costa Rica**	12004	12004	12005	12004	12003	12003	12003	12004	12005	12003	12004	12006
**Guatemala**	679	679	681	683	684	684	684	684	685	684	685	684
**Mexico**	355548	355546	355633	355720	355712	355712	355697	355699	355700	355692	355694	355697
**Brazil**	74160	74159	74189	74191	74194	74194	74198	74197	74195	74203	74201	74198
**Chile**	3491	3490	3489	3493	3493	3492	3491	3489	3488	3491	3489	3488
**Ecuador**	5624	5625	5625	5628	5627	5627	5627	5626	5625	5627	5626	5626
**Paraguay**	53259	53257	53279	53309	53305	53305	53304	53305	53311	53304	53305	53310
**Estonia**	4935	4935	4947	4956	4956	4955	4959	4958	4957	4962	4960	4957
**United Kingdom**	121480	121482	121496	121519	121518	121516	121520	121517	121511	121519	121515	121510
**Czech Republic**	15943	15942	15942	15953	15952	15951	15951	15950	15948	15950	15949	15948
**Germany**	11535	11535	11539	11541	11541	11542	11541	11542	11543	11540	11541	11543
**Romania**	11473	11472	11489	11500	11498	11496	11497	11498	11503	11498	11500	11506
**Switzerland**	124193	124193	124208	124233	124235	124237	124237	124238	124236	124235	124236	124234
**Italy**	5252	5251	5250	5251	5253	5254	5253	5253	5252	5251	5251	5252
**Spain**	96765	96765	96884	96961	96961	96958	96967	96963	96962	96971	96966	96963
**China**	7802	7802	7805	7808	7808	7809	7809	7809	7809	7809	7809	7809
**Japan**	2010928	2010939	2011002	2011069	2011069	2011064	2011029	2011024	2011034	2011019	2011015	2011034
**South Korea**	25446	25447	25549	25627	25635	25632	25642	25637	25637	25640	25635	25642
**Philippines**	2428	2428	2421	2423	2423	2423	2422	2422	2421	2422	2422	2421
**Taiwan**	51194	51194	51198	51204	51204	51204	51202	51203	51203	51202	51203	51203
**Vietnam**	1677	1677	1677	1674	1676	1678	1679	1680	1682	1681	1681	1682
**Australia**	7641	7641	7645	7648	7648	7648	7648	7648	7647	7648	7648	7647
**South Africa**	22896	22896	22912	22934	22934	22934	22934	22934	22932	22934	22935	22933
**Total**	3472531	3472541	3473319	3474122	3474107	3474092	3474068	3474047	3474035	3474069	3474046	3474046

The values represent the sum of quasi Akaike's Information Criterion (QAIC) values for each country. For the first-stage model selection, 426 locations were used, excluding 307 locations in Finland, 14 locations in Germany, and 4 locations in Australia that were remotely analyzed. The model selection was determined based on the total sum of QAIC values across all countries, ensuring the optimal balance between model complexity and goodness of fit.

Table S5 | Variable selection for the second-stage multivariate mixed-effects meta-regression model used to pool location-specific exposure–response associations across n = 751 locations. Candidate meta-predictors along with random effects at the country/city level were evaluated and selected based on the Akaike information criterion (AIC).

Model	Predictor	[a]Test for predictor	AIC	BIC	[b]Q-test	[c]I^2
Intercept only	(intercept)	-	1775.1	1798.6	0.001	14.4%
1 predictor	region	<0.001	1846.6	1955.2	0.026	8.9%
	gdp	<0.001	1883.1	1916.3	0.001	13.3%
	avg temp	0.257	1907.8	1941.0	0.000	17.9%
	range temp	0.246	1907.7	1940.9	0.000	17.6%
3 predictors	region	<0.001	1843.2	1970.6	0.056	7.3%
	avg temp	0.313				
	range temp	0.030				
4 predictors	region	0.006	1842.5	1979.2	0.074	6.7%
	gdp	0.086				
	avg temp	0.440				
	range temp	0.303				
3 predictors + region/country	region	< 0.001	1847.2	1988.7	0.056	7.3%
	avg temp	0.190				
	range temp	0.255				
3 predictors + country/city	region	< 0.001	1841.0	1982.5	0.056	7.3%
	avg temp	0.108				
	range temp	0.157				
4 predictors + region/country	region	0.024	1846.6	1997.4	0.074	6.7%
	gdp	0.057				
	avg temp	0.217				
	range temp	0.335				
4 predictor + country/city	region	< 0.001	1840.9	1991.794	0.074	6.7%
	gdp	0.155				
	avg temp	0.149				
	range temp	0.268				

Based on the overall assessment of these statistical tests, the model incorporating four predictors along with random effects at the country/city level was determined to be the optimal choice. A total of 751 locations are used to select a two-stage model.

a. Test for Predictor Means: p-value from the multivariate Wald test, which evaluates the significance of the meta-predictors included in the model.
b. Q-test: p-value for the Cochran Q-test, which assesses the presence of heterogeneity among the included studies.

c. I^2: the proportion of total variation in effect estimates attributable to heterogeneity rather than chance, indicating the extent to which the observed variability is due to differences between studies rather than within-study sampling error.

Table S6 | Summary of selected locations where the minimum projected temperature in the 2050s under the high-emission scenario (SSP5-8.5) and the observed median temperature, used to validate the appropriateness of the 50th percentile as the reference temperature. SSP, shared socioeconomic pathway.

Region	Country	Location	Minimum temperature in 2050s	Percentile of observed temperature
North America	Canada	Victoria	3.8	7.7%
	United States	Miami, FL	15.1	2.2%
Central America	Costa Rica	SanJose	22.4	43.0%
	Guatemala	Guatemala	16.0	2.9%
	Mexico	Guadalajara	17.0	8.2%
South America	Brazil	Fortaleza	27.6	49.9%
	Chile	Santiago	5.9	3.0%
	Ecuador	Guayaquil	25.3	32.7%
	Paraguay	Asuncion	11.2	2.3%
North Europe	Estonia	Tallinn	-9.1	4.2%
	Finland	Vieremä	1.6	46.8%
	United Kingdom	South West	2.3	4.6%
Central Europe	Czech Republic	Brno	-5.0	3.3%
	Germany	Dusseldorf	-2.4	2.0%
	Romania	Constanta	-2.7	3.6%
	Switzerland	Ticino	1.9	4.8%
South Europe	Italy	Rome	4.6	3.3%
	Spain	Tenerife	17.9	10.7%
East Asia	China	Suzhou	3.1	4.2%
	Japan	Okinawa	15.4	6.2%
	South Korea	Seogyupo	5.4	5.3%
Southeast Asia	Philippines	Manila	26.7	11.7%
	Taiwan	Kaohsiung	17.5	3.7%
	Vietnam	Ho Chi Minh	27.2	17.0%
Australia	Australia	Brisbane	14.4	7.4%
South Africa	South Africa	uMkhanyakude	18.0	10.2%

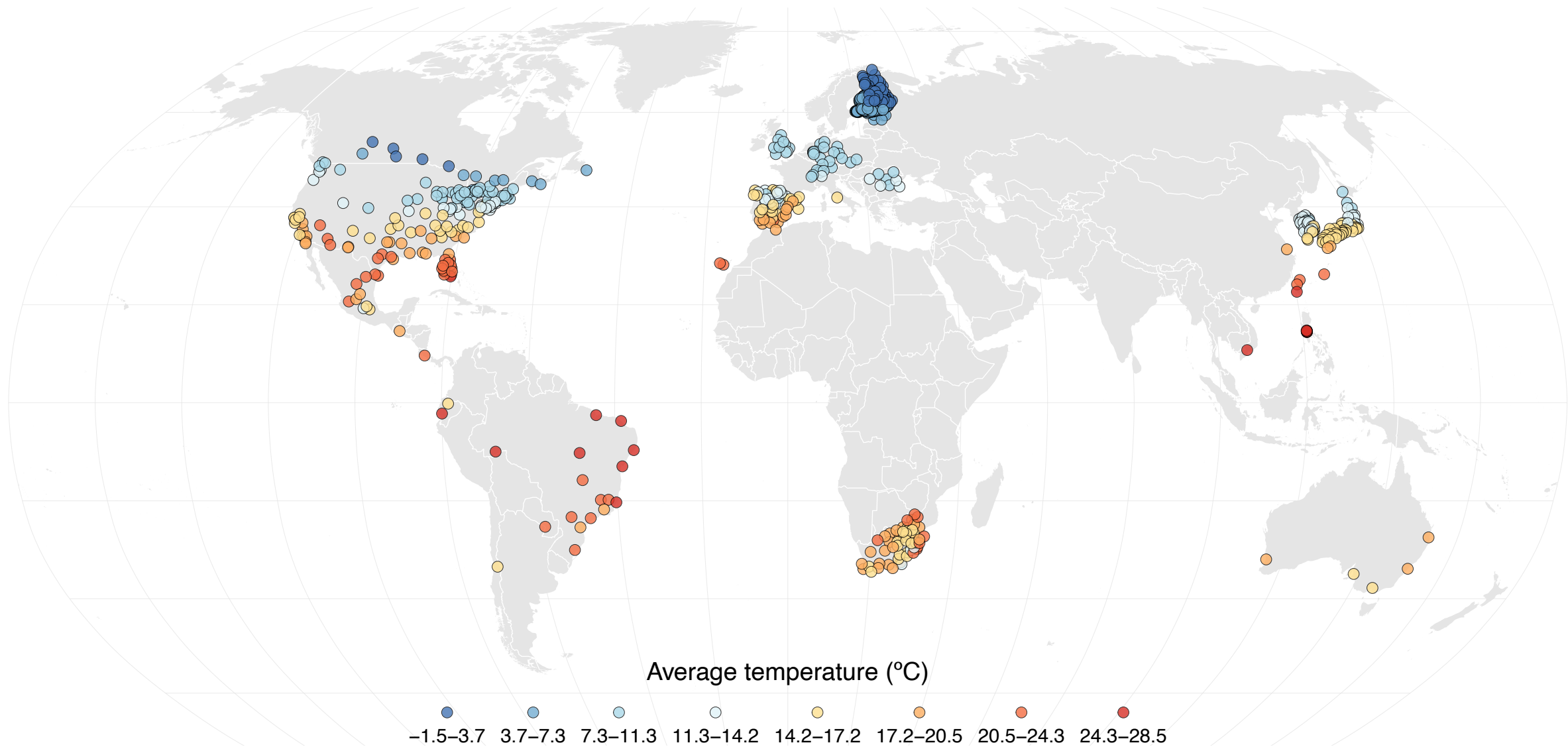

Fig. S1 | Map of the average observed temperatures at the 751 locations included in the analysis. Colours represent the mean daily temperature (°C) during the observation period at each location. Map data © EuroGeographics, sourced via the 'giscoR 0.6.1' R package (CC BY 4.0). The colors represent various ranges of average daily temperature calculated during the observation period for each country, as shown in Table 1.

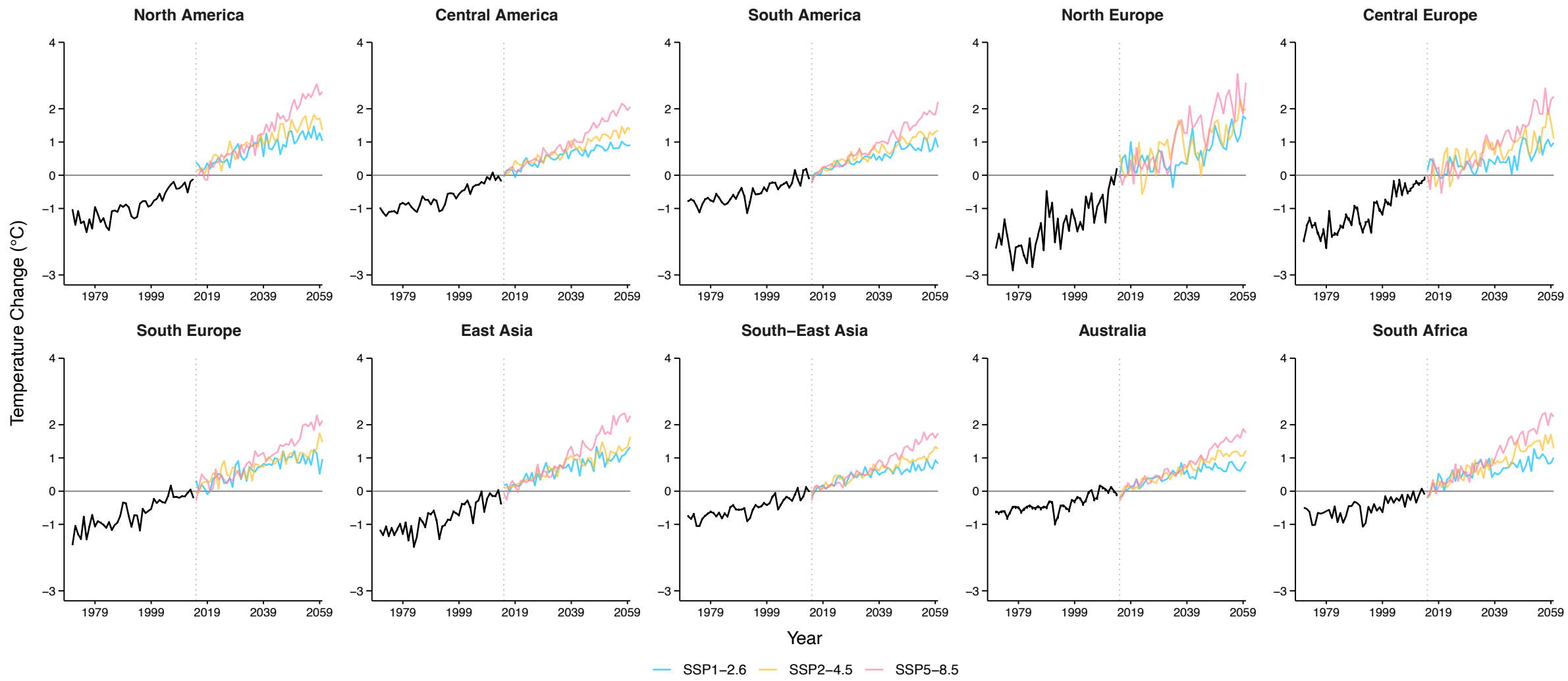

Fig. S2 | Decadal changes in mean temperature (°C) relative to the 2010s by region and climate scenario (SSP1-2.6, SSP2-4.5, and SSP5-8.5). The historical period spans from 1 January 1971 to 31 December 2014, representing deviations from the 2010s mean temperature. Projected temperatures from 2015 to 2059 are shown by color lines for each scenario, representing changes from the same 2010s. GCM, general circulation model; SSP, shared socioeconomic pathway.

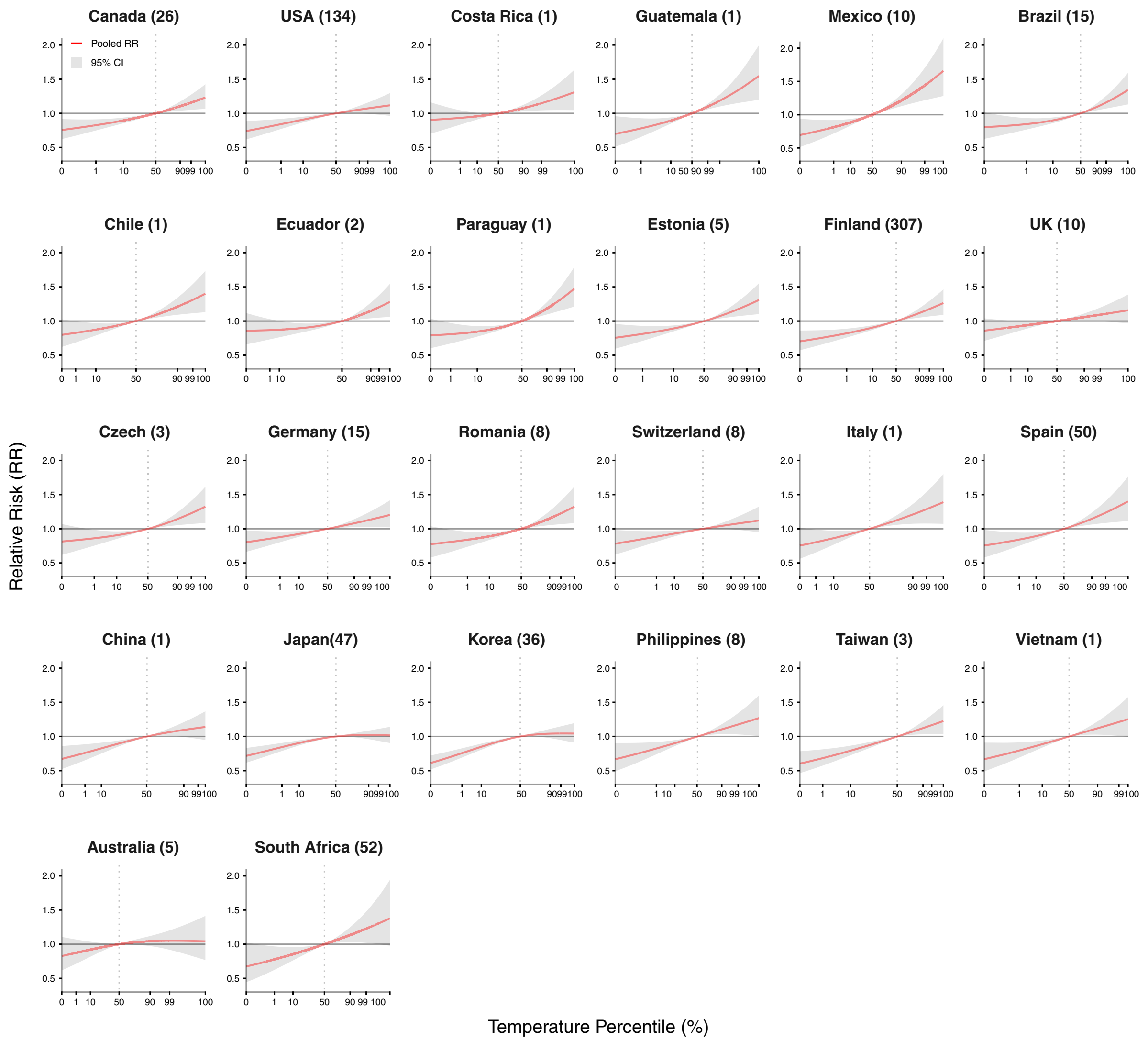

Fig. S3 | Overall cumulative relationship between temperature and suicide by country. The red line represents the pooled relative risk (RR) of suicide mortality across the temperature distribution, estimated from a two-stage meta-regression pooling location-specific exposure–response associations (n = 751 locations across 26 countries). The measure of center and shaded area display the pooled RR estimates and their corresponding 95% CIs. The dotted vertical line indicates the reference temperature (50th percentile of the country-specific temperature distribution), at which RR = 1. RR, relative risk; CI, confidence interval.

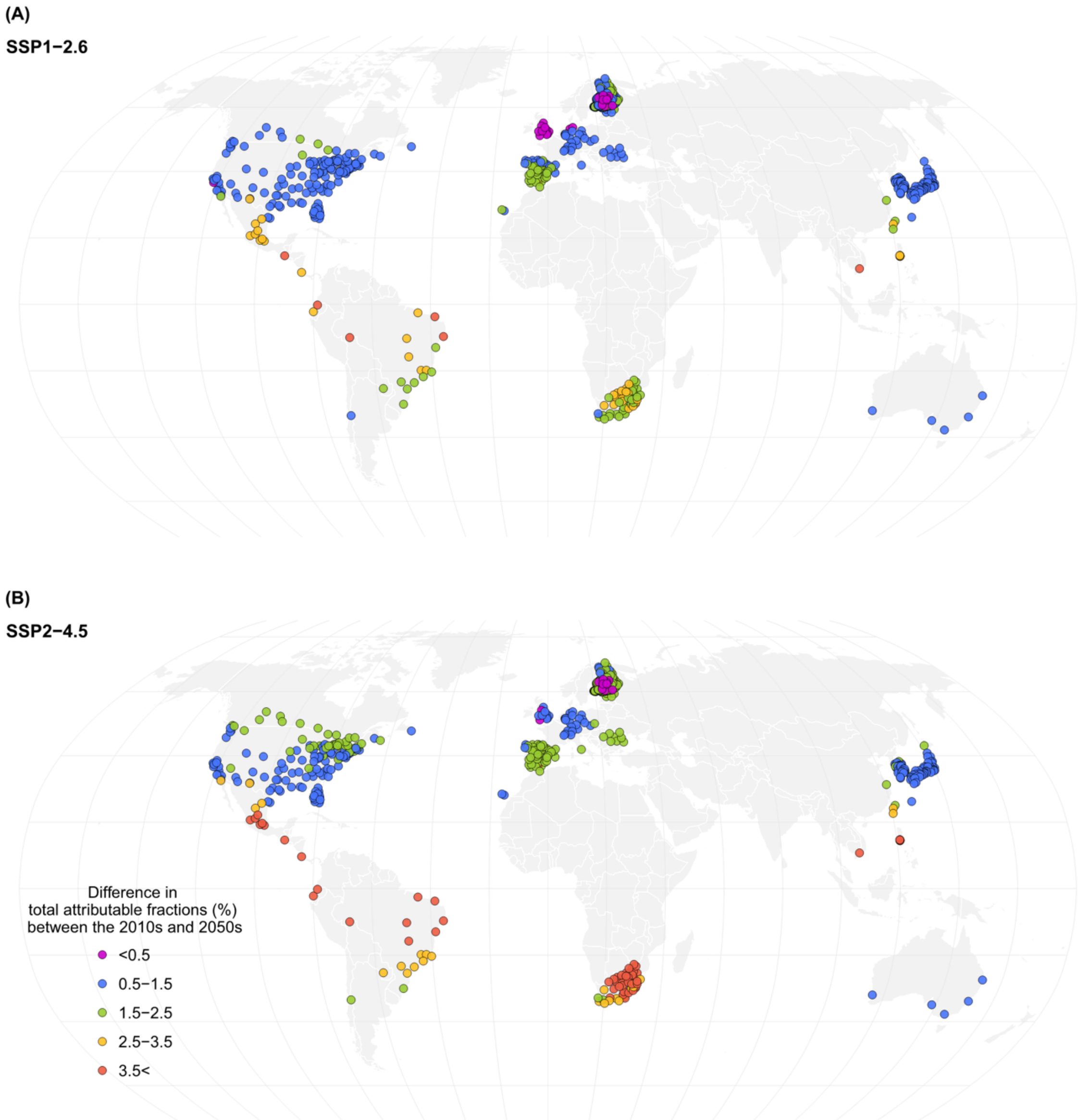

Fig. S4 | Map of projected changes in total attributable fraction (AF, %) of suicide mortality associated with temperatures between the 2010s (2010–2019) and 2050s (2050–2059) under two climate change scenarios: (A) SSP1-2.6 and (B)SSP2-4.5, across 751 locations in 26 countries. Map data © EuroGeographics, sourced via the ‘giscoR 0.6.1’ R package (CC BY 4.0). AF, attributable fraction; SSP, shared socioeconomic pathway.

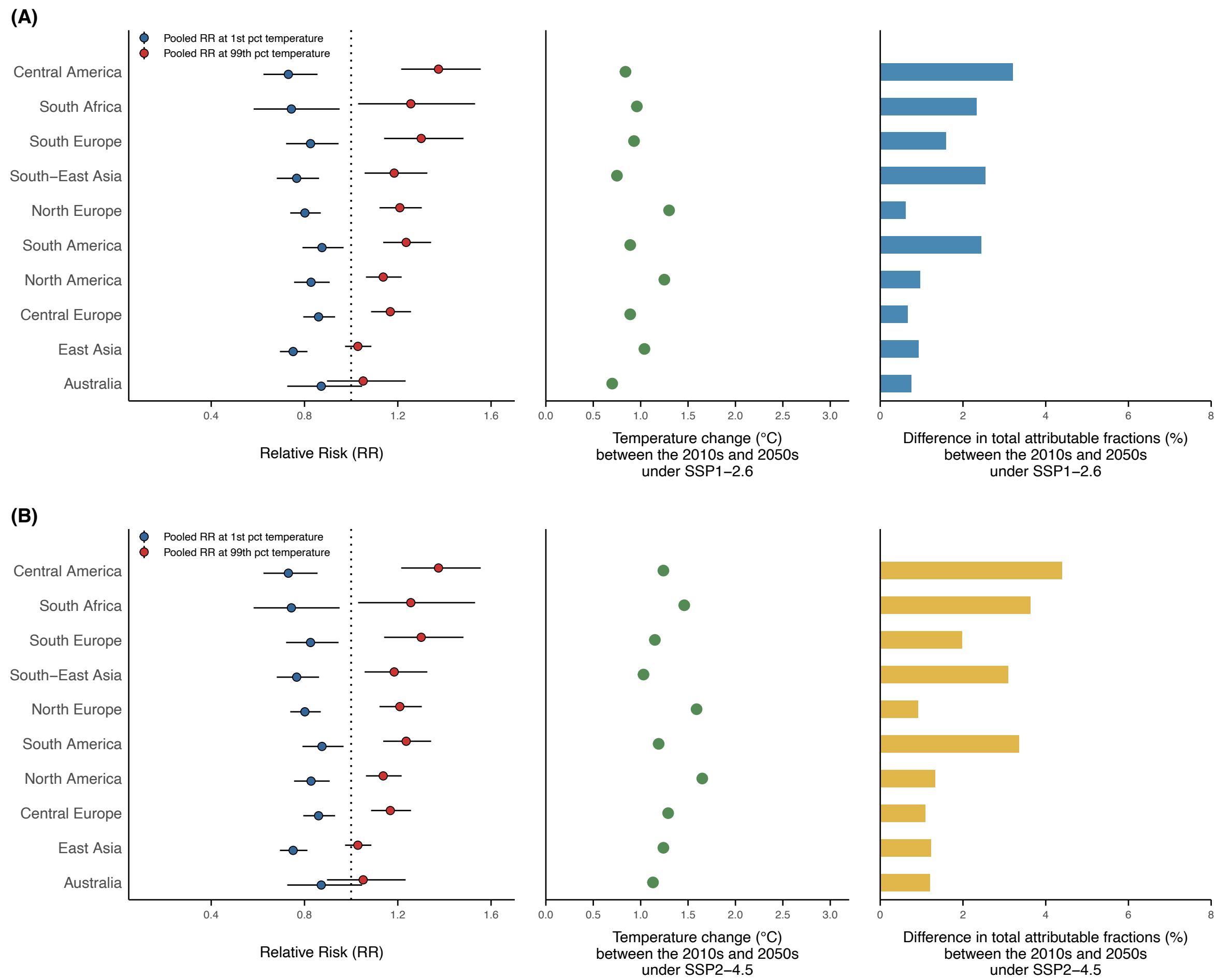

Fig. S5 | Regional relative risks of suicide mortality at temperature extremes, projected warming, and change in total attributable fractions of suicide mortality under two climate change scenarios: (A) SSP1-2.6 and (B) SSP2-4.5. The measure of center and error bars display the pooled RR values at the 1st and 99th percentiles of the regional temperature distribution and their corresponding 95% CIs, derived from a two-stage meta-regression (n = 751 locations across 10 regions and 26 countries). Points in panel (b) represent the mean projected temperature change (°C) averaged across n = 5 GCMs. Bars in panel (c) represent the mean difference in total AF averaged across n = 5 GCMs. RR, relative risk; AF, attributable fraction; GCM, general circulation model; SSP, shared socioeconomic pathway; CI, confidence interval.

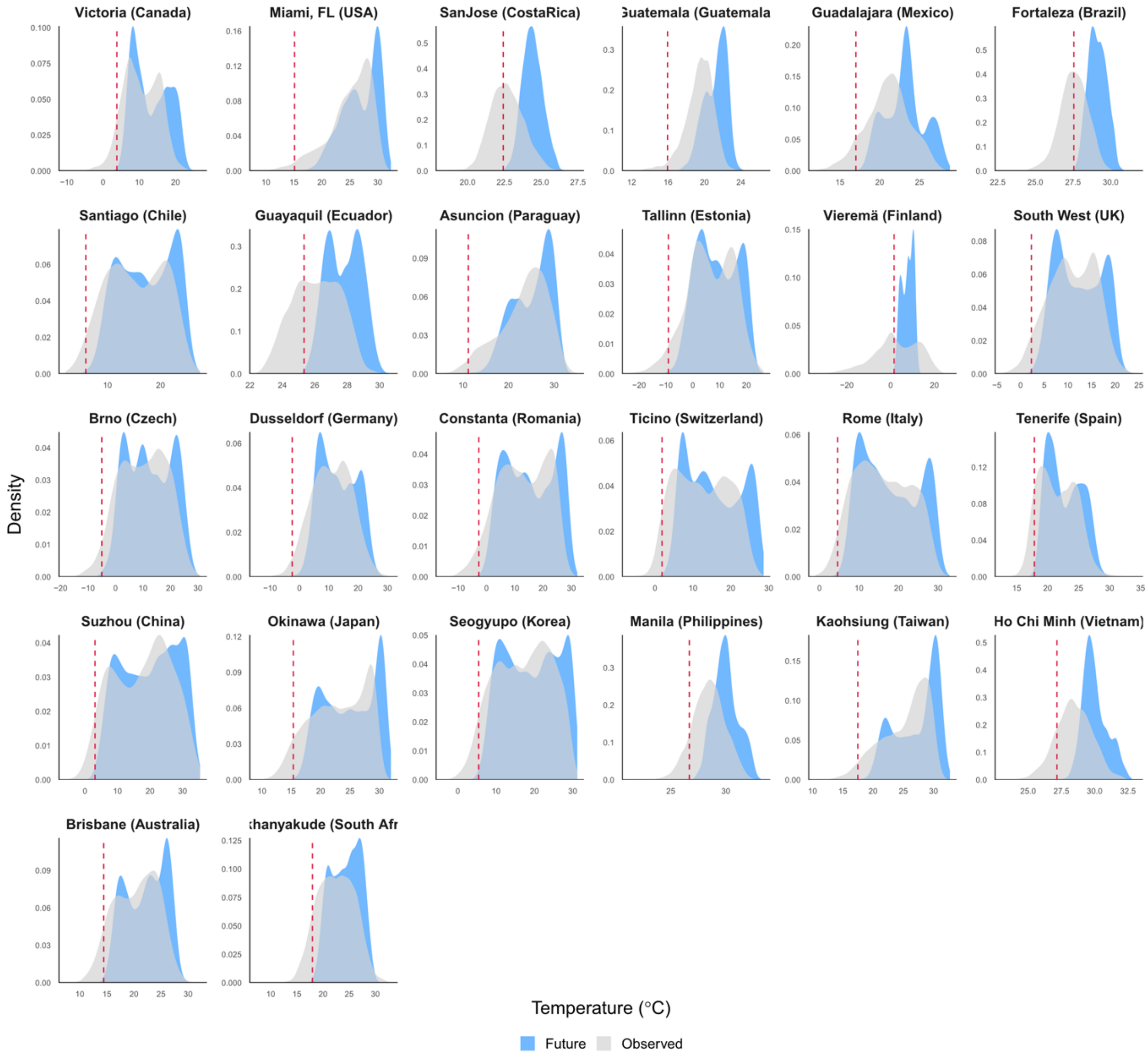

Fig. S6 | Temperature distribution in the 2050s (2050–2059) under the high-emission scenario (SSP5-8.5) compared to the observed temperature distribution for each of the 26 selected locations. Distributions represent the ensemble mean across n = 5 GCMs. SSP, shared socioeconomic pathway; GCM, general circulation model.

In Fortaleza, Brazil, the lowest projected temperature under SSP5-8.5 in the 2050s is 27.6°C, which corresponds to the 50th percentile of the observed temperature distribution (in Table S3).

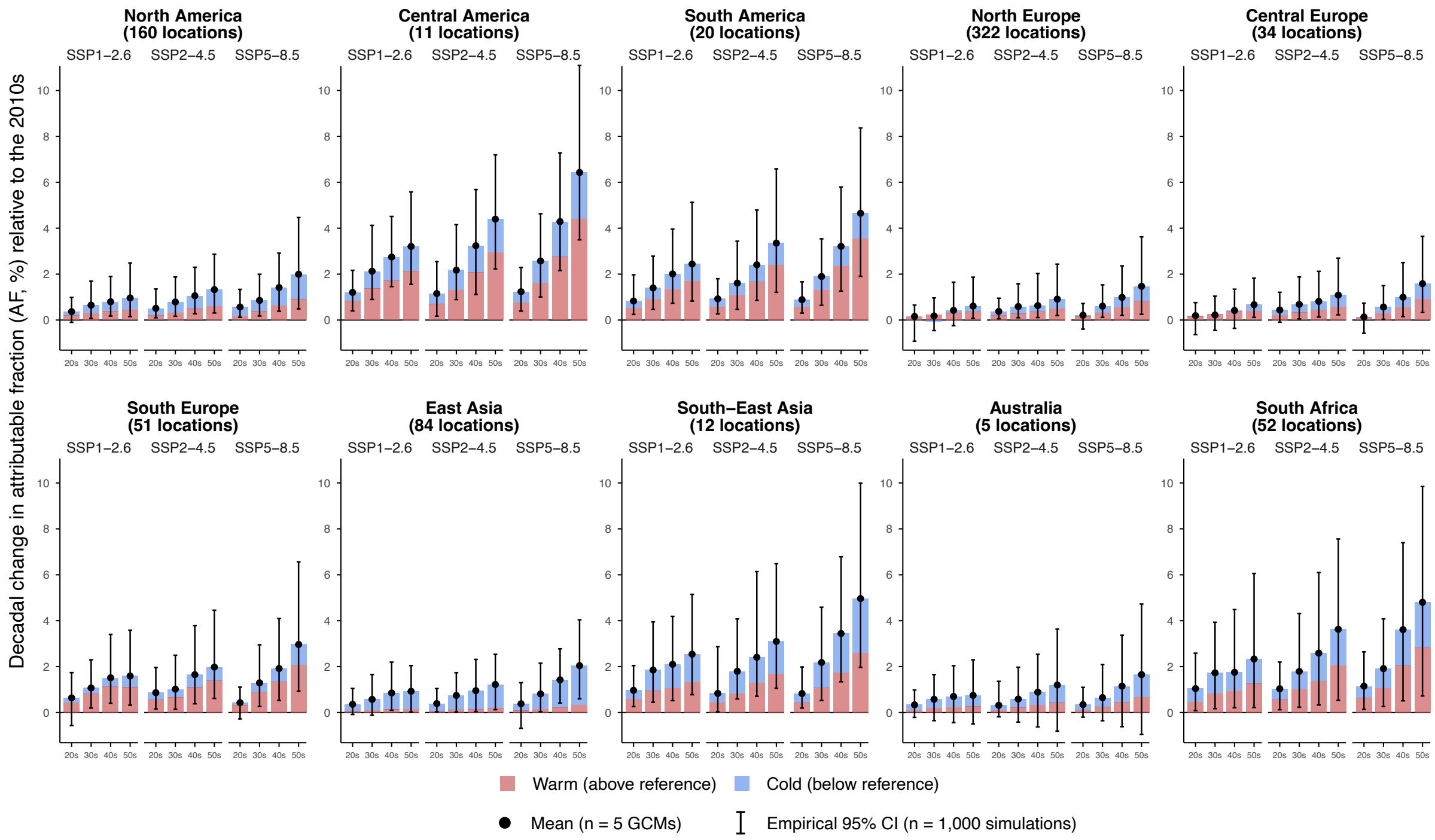

Fig. S7 | Temporal changes in attributable fraction (AF, %) of suicide mortality associated with warm (above reference temperature) and cold (below reference temperature) temperatures for each decade relative to the 2010s by region under three climate change scenarios (SSP1-2.6, SSP2-4.5, and SSP5-8.5). The measure of center and error bars display the mean values and 95% eCIs derived from n = 1,000 Monte Carlo simulations across n = 5 GCMs. Regional estimates were aggregated from locations within each corresponding region (total n = 751 locations across 10 regions).

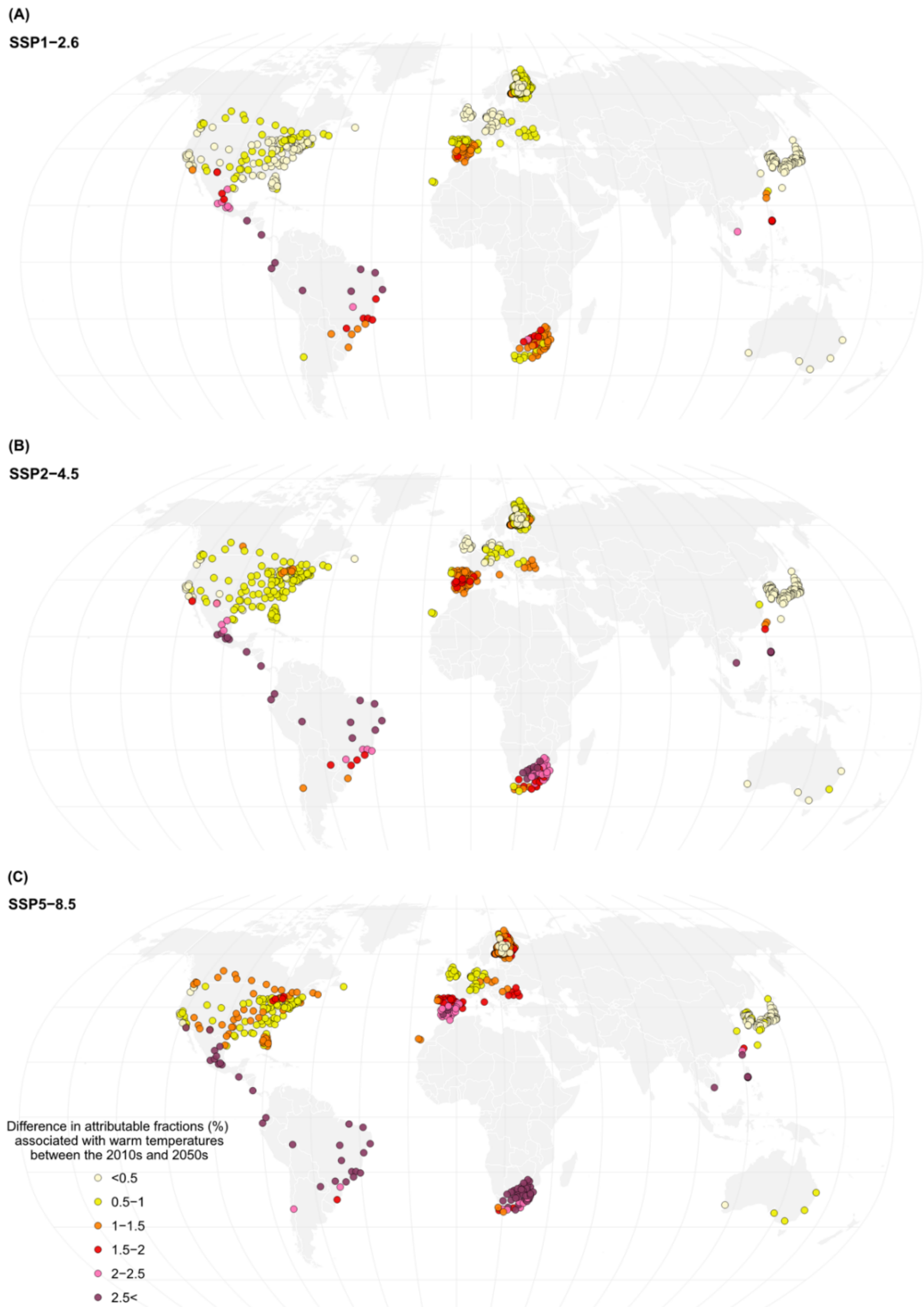

Fig. S8 | Map of projected changes in attributable fractions (AF, %) of suicide mortality associated with warm temperatures (above the reference temperature) between the 2010s (2010–2019) and 2050s (2050–2059) under three climate change scenarios: (A) SSP1-2.6, (B) SSP2-4.5, and (C) SSP5-8.5, across n = 751 locations in 26 countries. Map data © EuroGeographics, sourced via the 'giscoR 0.6.1' R package (CC BY 4.0). AF, attributable fraction; SSP, shared socioeconomic pathway.

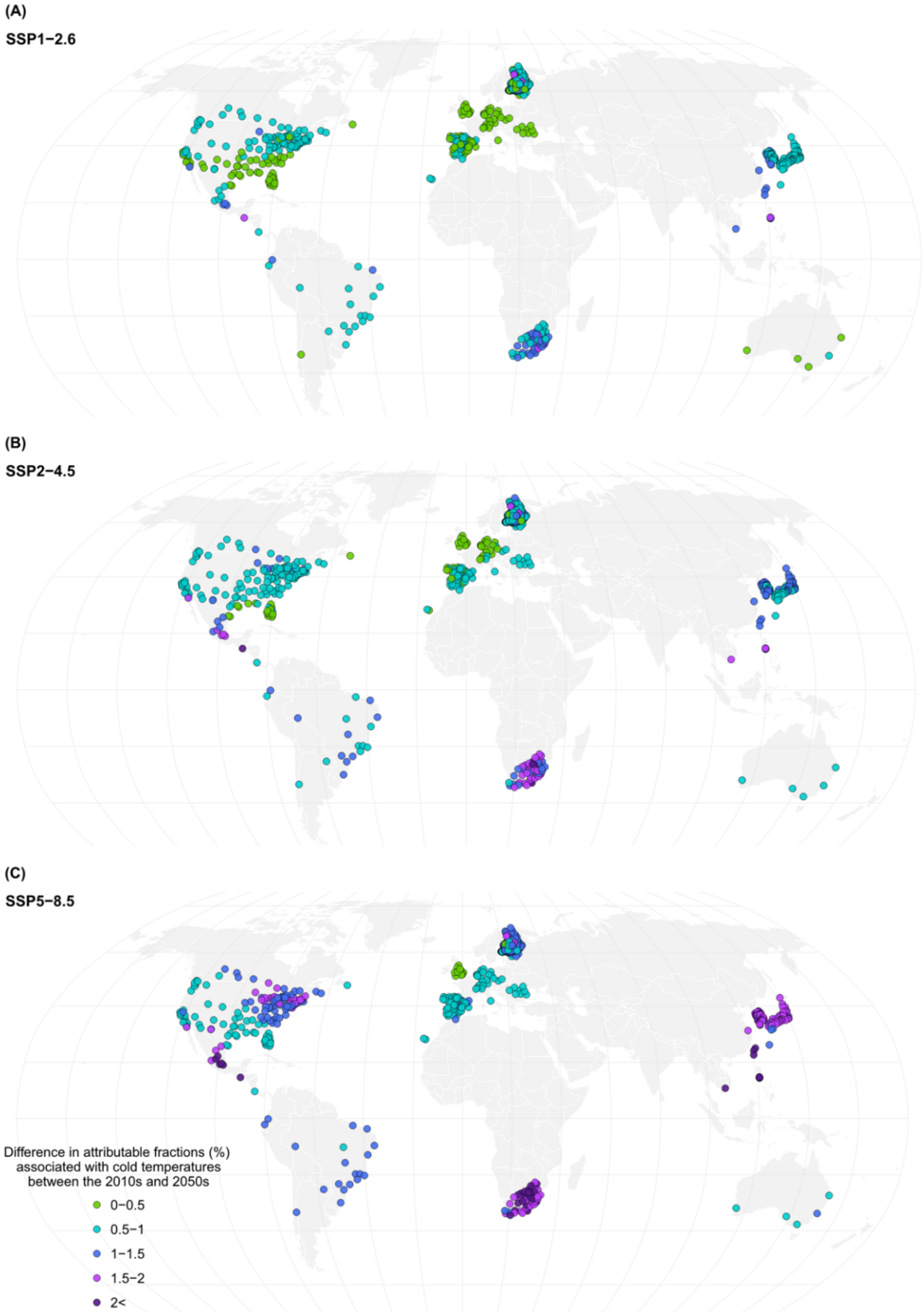

Fig. S9 | Map of projected changes in attributable fractions (AF, %) of suicide mortality associated with cold temperatures (below the reference temperature) between the 2010s (2010–2019) and 2050s (2050–2059) under three climate change scenarios: (A) SSP1-2.6, (B) SSP2-4.5, and (C) SSP5-8.5, across n = 751 locations in 26 countries. Map data © EuroGeographics, sourced via the 'giscoR 0.6.1' R package (CC BY 4.0). AF, attributable fraction; SSP, shared socioeconomic pathway.

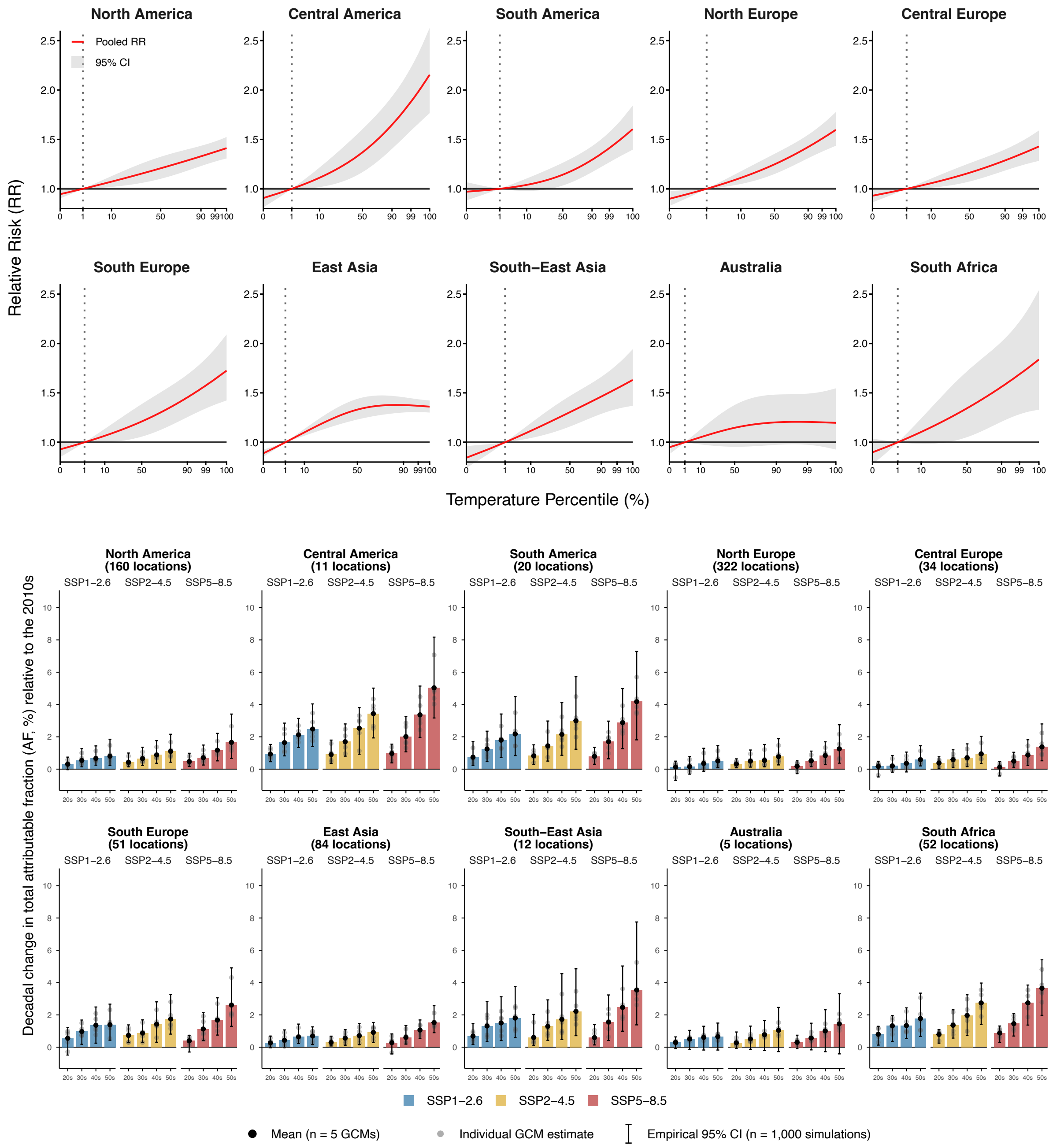

Fig. S10 | Temperature–suicide associations and temporal changes in total attributable fractions (AF, %) using the minimum suicide temperature (MinST) as the reference temperature. The upper panels show the overall cumulative exposure–response curves by region with the MinST as the reference, estimated from a two-stage meta-regression (n = 751 locations across 10 regions). The measure of center and shaded area display the pooled RR estimates and their corresponding 95% CIs. The lower panels show the decadal changes in total AF relative to the 2010s under three climate change scenarios (SSP1-2.6, SSP2-4.5, and SSP5-8.5). Data are presented as mean values (bars) ± 95% eCIs (error bars) derived from n = 1,000 Monte Carlo simulations across n = 5 GCMs. RR, relative risk; AF, attributable fraction; SSP, shared socioeconomic pathway; CI, confidence interval.

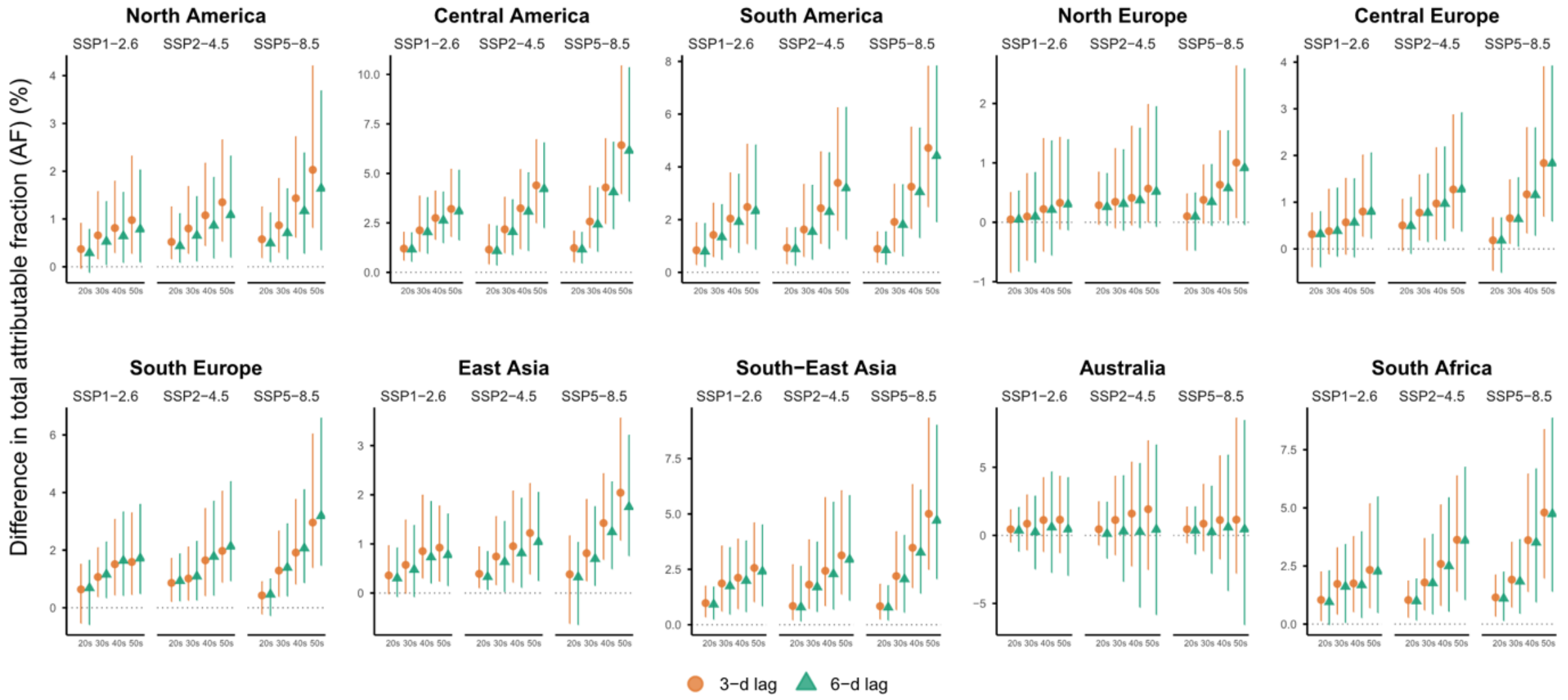

Fig. S11 | Sensitivity analysis comparing temporal changes in total attributable fraction (AF, %) of suicide mortality using short-term lag structures of 3-day and 6-day lags, based on data from n = 426 locations. Data are presented as mean values (bars) ± 95% eCIs (error bars) derived from n = 1,000 Monte Carlo simulations across n = 5 GCMs. Gray circles overlaid on each bar show individual GCM-level estimates (n = 5 per bar). AF, attributable fraction; GCM, general circulation model; eCI, empirical confidence interval.

References

1. Armstrong, B. G., Gasparrini, A. & Tobias, A. Conditional Poisson models: A flexible alternative to conditional logistic case cross-over analysis. *BMC Med Res Methodol* 14(1), 122 (2014)..
2. Lu, Y., Symons, J. M., Geyh, A. S. & Zeger, S. L. An approach to checking case-crossover analyses based on equivalence with time-series methods. *Epidemiology* 19(2), 169–175 (2008).
3. Gasparrini, A., Armstrong, B. & Kenward, M. G. Distributed lag non-linear models. *Stat Med* 29(21), 2224–2234 (2010).
4. Gasparrini, A. et al. Projections of temperature-related excess mortality under climate change scenarios. *Lancet* 1(9), e360–e367 (2017).
5. Vicedo-Cabrera, A. M., Sera, F. & Gasparrini, A. Hands-on tutorial on a modeling framework for projections of climate change impacts on health. *Epidemiology* 30(3), 321–329 (2019).
6. Masselot, P. & Gasparrini, A. Modelling extensions for multi-location studies in environmental epidemiology. *Stat Methods Med Res* 34(3), 615–629 (2025).
7. Thawonmas, R., Hashizume. M. & Kim, Y. Projections of temperature-related suicide under climate change scenarios in Japan. *Environ Health Perspect* 131(11), 117012 (2023).
8. Kim, Y. et al. Suicide and ambient temperature: A multi-country multi-city study. *Environ Health Perspect* 127(11), 117007 (2019).
9. Gasparrini, A. & Armstrong, B. Reducing and meta-analyzing estimates from distributed lag non-linear models. *BMC Med Res Methodol* 13(1), 1 (2013).
10. Sera, F., Armstrong, B., Blangiardo, M. & Gasparrini, A. An extended mixed-effects framework for meta-analysis. *Stat Med* 38(29), 5429–5444 (2019).
11. Higgins, J. P. & Thompson, S. G. Quantifying heterogeneity in a meta-analysis. *Stat Med* 21(11), 1539–1558 (2002).
12. Gasparrini, A. & Leone, M. Attributable risk from distributed lag models. *BMC Med Res Methodol* 14(1), 55 (2014).
